# Late-stage peptide labeling with near-infrared fluorogenic nitrobenzodiazoles by manganese-catalyzed C–H activation†

**DOI:** 10.1039/d3sc01868g

**Published:** 2023-05-04

**Authors:** Tsuyoshi Oyama, Lorena Mendive-Tapia, Verity Cowell, Adelina Kopp, Marc Vendrell, Lutz Ackermann

**Affiliations:** a Institut für Organische und Biomolekulare Chemie, Georg-August-Universität Göttingen Tammanstraße 2 37077 Göttingen Germany lutz.ackermann@chemie.uni-goettingen.de; b Centre for Inflammation Research, The University of Edinburgh EH16 4TJ Edinburgh UK marc.vendrell@ed.ac.uk; c German Center for Cardiovascular Research (DZHK) Potsdamer Straße 58 10785 Berlin Germany

## Abstract

Late-stage diversification of structurally complex amino acids and peptides provides tremendous potential for drug discovery and molecular imaging. Specifically, labeling peptides with fluorescent tags is one of the most important methods for visualizing their mode of operation. Despite major recent advances in the field, direct molecular peptide labeling by C–H activation is largely limited to dyes with relatively short emission wavelengths, leading to high background signals and poor signal-to-noise ratios. In sharp contrast, here we report on the fluorescent labeling of peptides catalyzed by non-toxic manganese(i) *via* C(sp^2^)–H alkenylation in chemo- and site-selective manners, providing modular access to novel near-infrared (NIR) nitrobenzodiazole-based peptide fluorogenic probes.

## Abstract 2

This manganese-catalyzed late-stage NBD peptide labeling featured excellent site- and diastereo-*E*-selectivity, enabling large Stokes shifts, fluorogenic NIR emission, and application for real-time imaging of live bacteria under wash free conditions.

## Graphical abstract

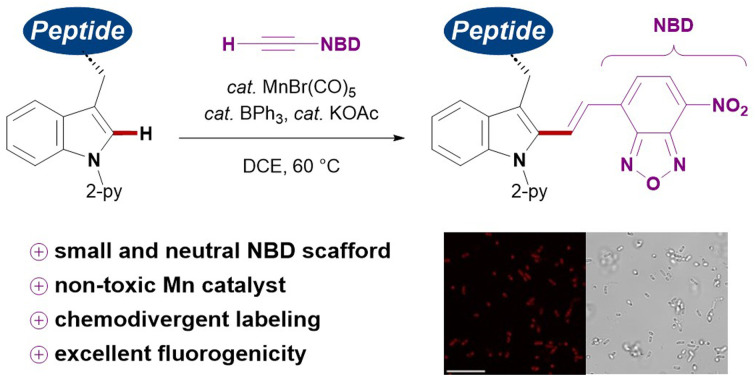

## Introduction

**Scheme 1:**
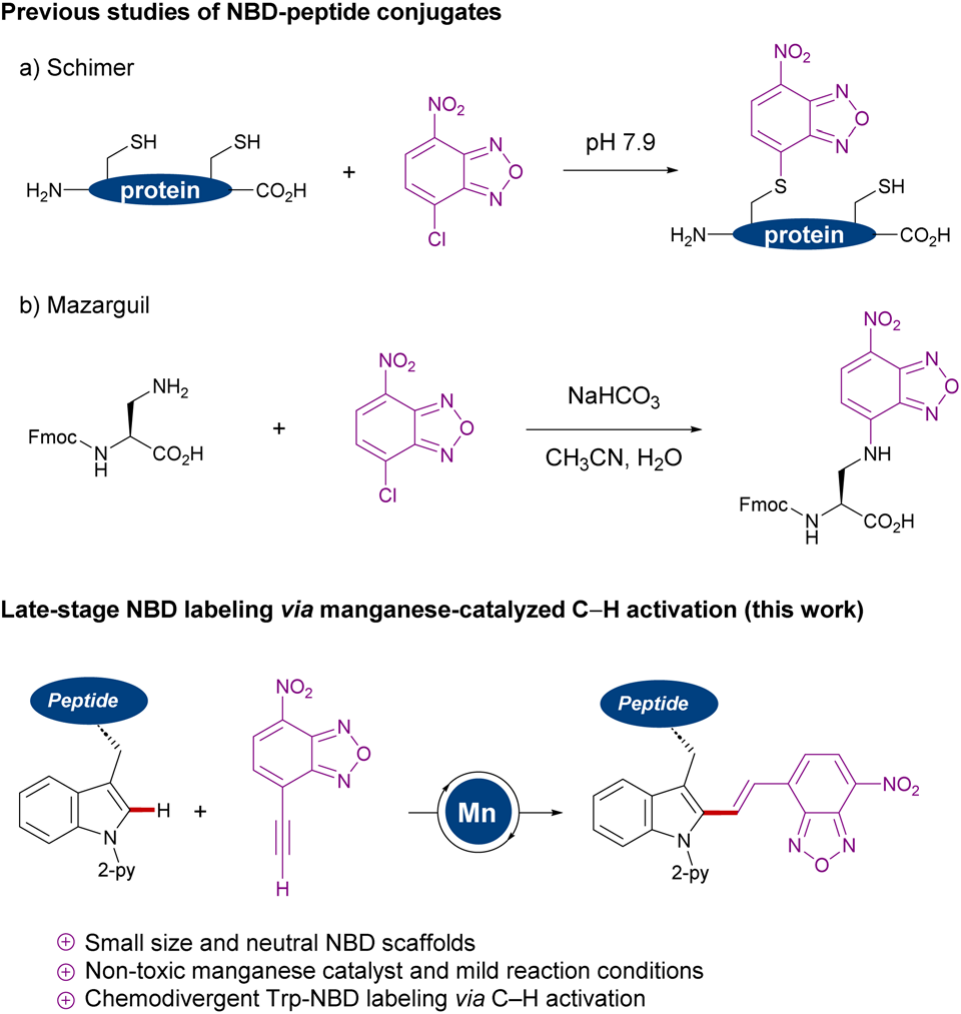
Nucleophilic NBD labeling (a and b) *versus* manganese(i)-catalyzed NBD labeling of tryptophans.

Among the vast array of pharmaceutical compounds, peptides have emerged as powerful candidates for drug development.^1^ Since the first medical use of insulin in 1921,^1^ the field of peptide-based drugs has progressively grown, currently accounting for 5% of the global pharmaceutical market and with approvals steadily increasing. Many therapeutic peptides operate by binding to cell membrane components with high affinity, triggering specific intracellular effects.^2^ In particular, antimicrobial peptides are emerging as a promising alternative to combat antimicrobial drug-resistance.^3^ In this regard, fluorogenic labeling of peptides which enables real-time imaging studies is of key importance to analyze their functional mechanism and to develop new therapeutics.^4^ While a variety of chemical motifs have been employed in the development of fluorescent probes, those emitting in the near-infrared (NIR) window (650–900 nm) are attracting particular attention, since they have the advantages of low autofluorescence of biomolecules as well as low toxicities due to light irradiation at relatively long excitation wavelengths.^5^ Nitrobenzodiazole (NBD) fluorescent dyes have been identified as powerful labeling tags because of their small size, neutral character, cell permeability and large Stokes shifts.^6^ In addition, Vendrell and Lee recently reported NBD derivatives with NIR fluorescent properties.^7^ Examples of NBD labeling peptide and protein were reported, including the modification of nucleophilic cysteine or 3-amino-alanine residues (Scheme 1a and b).^8^ Despite remarkable progress, the introduction of NBD is hence largely limited to the nucleophilic region, such as *N*-terminal groups, thiols or phenols using classical condensations or S_N_Ar type reactions.^8^

During the past decade, transition-metal-catalyzed C–H activation^9^ has surfaced as a transformative platform for the direct modification of peptides,^10^ with major contributions from Lavilla and Albericio,^11^ Chen,^12^ Wang,^13^ Shi,^14^ Yu,^15^ and Ackermann,^16^ among others.^17^ While significant progress has thereby been realized, molecular peptide labeling with NIR dyes has thus proven elusive. In sharp contrast, we now have identified a strategy to stitch tryptophan with NBD fluorophores *via* earth-abundant and non-toxic manganese(i)-catalyzed C–H activation,^18^ and applied this approach for real-time imaging by using a model peptide targeting bacterial cell membranes. This strategy has the power to facilitate structure–activity relationship studies of fluorescent probes with NBD, since there is no report of direct conjugation to tryptophan residues.

## Result and discussion

We initiated our studies by proving difficult reaction conditions with tryptophan derivative 1 and 4-ethynyl-7-nitrobenzoxadiazole (2) (Table 1). After optimization of reaction parameters (see details in the ESI Table S1†), we obtained the desired C_2_ functionalized product 3 in 95% yield in the presence of MnBr(CO)_5_, KOAc, and BPh_3_ (entry 1). Subsequently, we investigated the efficiency of other catalysts, such as ReBr(CO)_5_, which delivered product 3 only in moderate yield (entry 2). Likewise, significantly decreased catalytic activity was observed with Mn_2_(CO)_10_ (entry 3). It is noteworthy that Pd(OAc)_2_, [RuCl_2_(*p*-cymene)]_2_, [RhCp*Cl_2_]_2_, and Co(OAc)_2_ showed no catalytic efficiencies (entries 4–7), indicating the challenges associated with a chemo-selective NBD labeling. Interestingly, the manganese(i)-catalyzed C–H activation proceeded even at physiological temperature of 37 °C, delivering the desired product 3 in 78% yield (entry 8). Control experiments showed that manganese(i) catalyst, KOAc, and BPh_3_ were essential for the reaction (entries 9–11).

**Table 1:** Optimization of the reaction conditions^a^

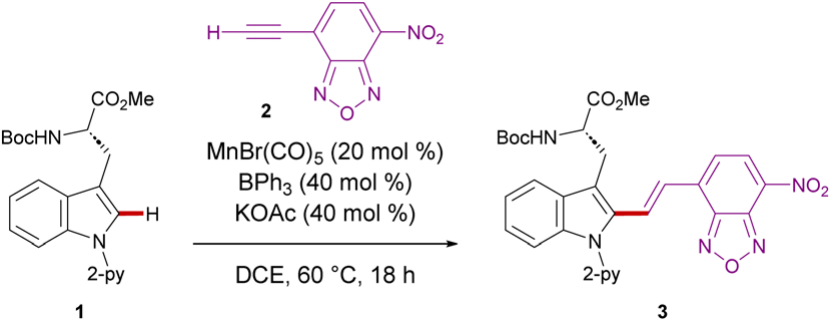		
Entry	Deviation from the standard conditions	Yield^c^ (%)
1	None^b^	95
2	ReBr(CO)_5_ in place of MnBr(CO)_5_	60
3	Mn_2_(CO)_10_ in place of MnBr(CO)_5_	Trace
4	Pd(OAc)_2_ in place of MnBr(CO)_5_	—
5	[RuCl_2_(*p*-cymene)]_2_ in place of MnBr(CO)_5_	—
6	[RhCp*Cl_2_]_2_ in place of MnBr(CO)_5_	—
7	Co(OAc)_2_ in place of MnBr(CO)_5_	—
8	At 37 °C	78
9	Without MnBr(CO)_5_	—
10	Without BPh_3_	4
11	Without KOAc	Trace

a DCE: 1,2-dichloroethane.

b Standard conditions: 1 (0.10 mmol), 2 (0.15 mmol), MnBr(CO)_5_ (20 mol%), BPh_3_ (40 mol%), KOAc (40 mol%), DCE (1.0 mL), 60 °C, 18 h.

c Isolated yields.

**Scheme 2:**
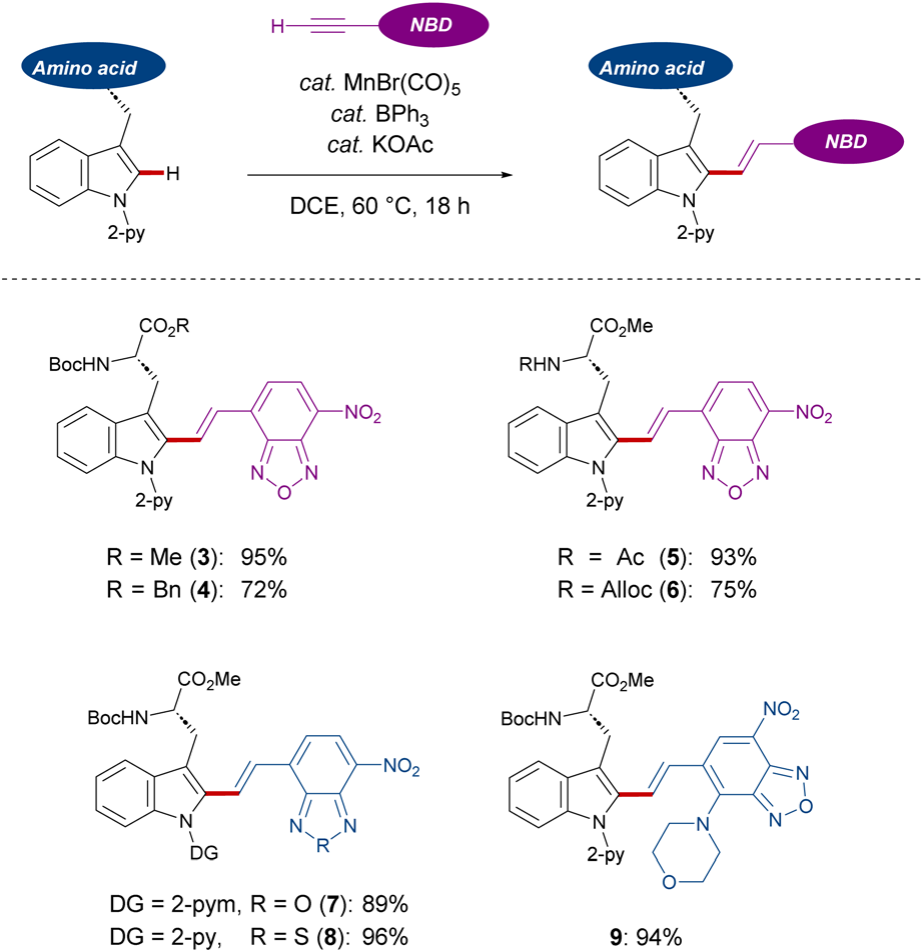
NBD labeling of tryptophans.

With the optimized reaction conditions for NBD-based fluorescence labeling in hand, we explored the generality of the Mn(i) catalysis (Scheme 2). The Mn(i) catalysis tolerated various tryptophans with Ac and Alloc groups at the *N*-terminus, generating the desired products 5 and 6 in good yields with complete diastereo-*E*-selectivities. It must be noted that benzyl esters at the C-terminus of tryptophan was compatible with our catalytic reaction conditions, offering the potential for further derivatization into peptides (4). In addition, NBD-alkynes bearing a different chalcogen or electron-donating group could also be converted using our strategy (8 and 9).

**Scheme 3:**
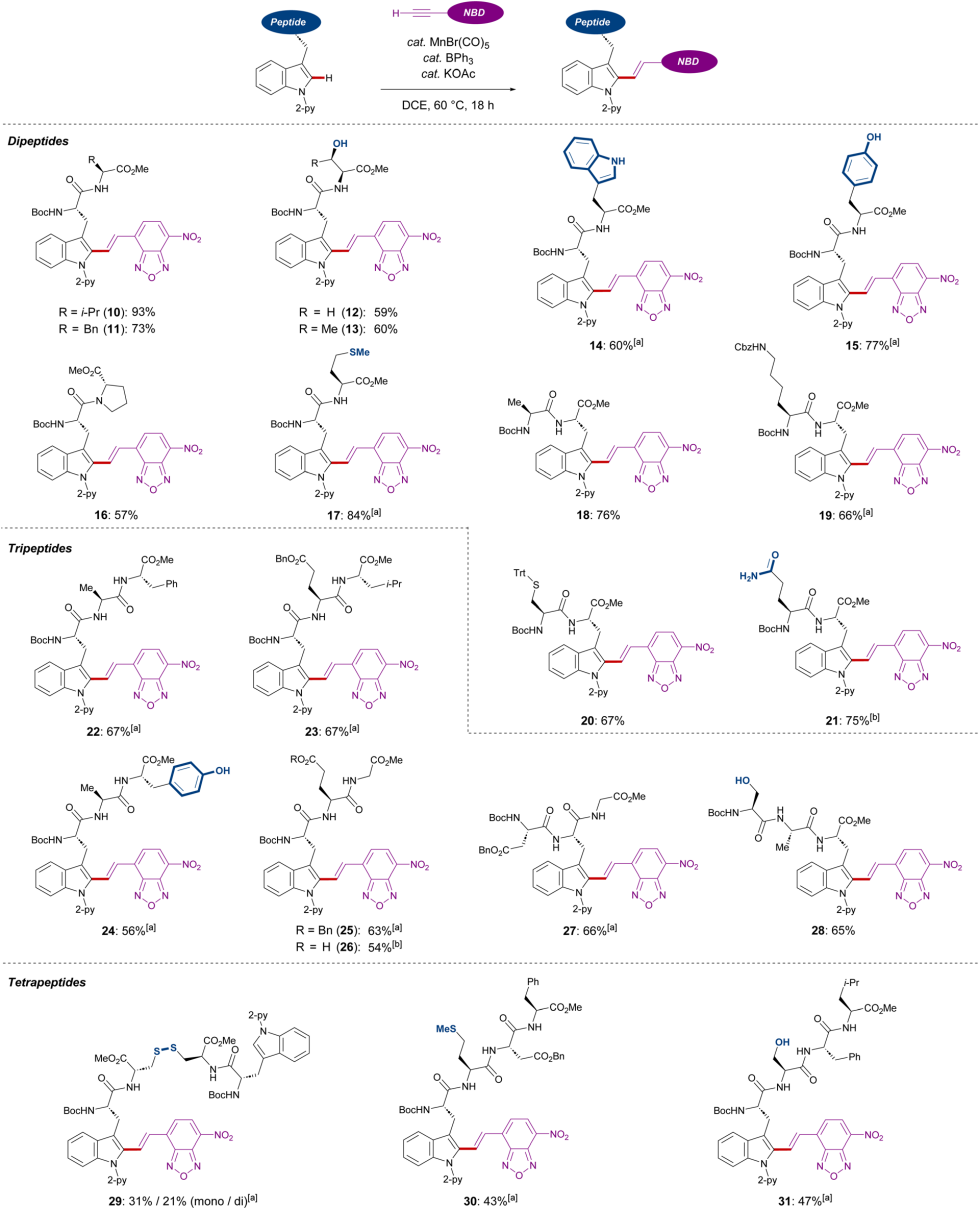
Robustness of C(sp^2^)–H alkenylation. [a] 0.050 mmol of peptide, 40 mol% of MnBr(CO)_5_, 80 mol% of KOAc and BPh_3_, 0.10 mmol of 2, and 1.0 mL of DCE were used. [b] 0.050 mmol of peptide, 0.050 mmol of MnBr(CO)_5_, 0.10 mmol of KOAc and BPh_3_, 0.15 mmol of 2, and 1.0 mL of DCE were used. Reaction time: 6 h.

Subsequently, we investigated the late-stage diversification of dipeptides. An outstanding chemo-selectivity was demonstrated by the complete tolerance of sensitive functional groups on the amino acid side chains. For instance, successful transformation of dipeptides with alkyl side chains were observed without epimerization (10, 11, 16, and 18). Furthermore, the presence of unprotected primary and secondary alcohols or phenols did not interfere with the reaction, furnishing the desired products 12, 13, and 15 in good yields. Oxidation-sensitive groups found in methionine and protected cysteine were also tolerated (17 and 20) (Scheme 3).

Interestingly, dipeptides containing an unmodified indole moiety delivered the desired alkenylated product with excellent site selectivity (14). Lastly, good tolerance of substituted amines and unprotected amides showed the robustness of these reaction conditions (19 and 21). Triggered by the versatility of our C–H alkenylation regime, we expanded to more challenging tri- and tetra-peptides which were converted in a site-, regio-, chemo-, and diastereo-*E*-selective manner (22–31). The robustness of our Mn(i) catalysis was also highlighted with the successful transformation of aspartic or glutamic acid-containing substrates (23, 25–27, and 30), which are known to cyclize into 5- or 6-membered amides.^19^ In addition, the side formation of dehydro-alanine derivatives was not observed, which can be formed in serine-containing substrates *via* β-elimination (28 and 31). It must be noted that a tetrapeptide containing a disulfide bond was also amenable to our Mn(i)-catalyzed labeling process (29).

**Fig. 1:**
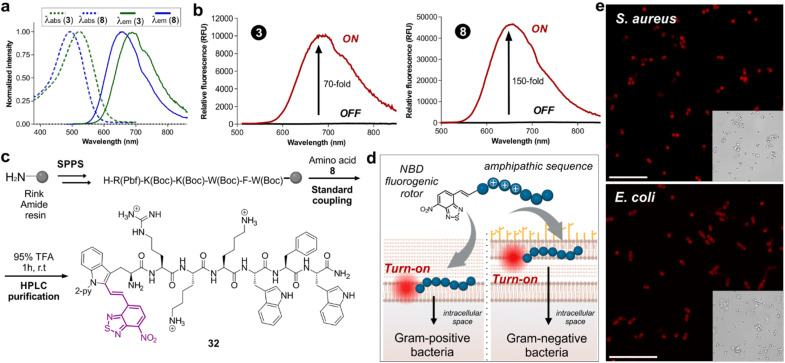
(a) Absorbance (dashed lines) and emission (solid lines) spectra of the amino acids 3 and 8. (b) Fluorescence emission spectra of the amino acids 3 and 8 (10 μM) in water (black line) and in dioxane (red line). *λ*_exc_: 480 nm (3) and 450 nm (8). Relative fluorescence quantum yields were determined using fluorescein as a standard: 0.7% for compound 3, 4.7% for compound 8. (c) Synthetic scheme for the preparation of bacterial-binding peptide 32 with amino acid 8. (d) Schematic illustration of the fluorescence activation of peptide 32 in both Gram-positive and Gram-negative bacteria with the environmentally-sensitive amino acid 8 as the fluorophore. (e) Fluorescence and brightfield microscopy images of Gram-positive bacteria (top panel, *S. aureus*) and Gram-negative bacteria (bottom panel, *E. coli*) after labelling with compound 32 (25 μM). Scale bar: 10 μm.

Next, we examined the fluorescence properties of the oxygen and sulfur NBD-labeled tryptophan derivatives 3 and 8, revealing absorption maxima wavelengths at 522 nm and 496 nm, and emission maxima wavelengths in the NIR-I window (688 nm and 658 nm respectively, Fig. 1a). Notably, both labeled amino acids exhibited very large Stokes shifts over 150 nm. Another important consideration is that NBD is relatively small compared to previously reported NIR-emitting scaffolds, helping to potentially retain the biological activity of the native peptides. Considering the potential molecular rotor features of our NBD-labeled tryptophan analogues, we also compared the turn-on effect of the amino acids 3 and 8 (Fig. 1b). As anticipated, both amino acids exhibited remarkable turn-on fluorescence in dioxane compared to H_2_O, with the S-containing compound 8 showing over 150-fold fluorescence increase in hydrophobic environments. Encouraged by the excellent fluorogenicity of the amino acid 8, we decided to use it for solid-phase peptide synthesis (SPPS) of the bacterial-targeting sequence 32 (Fig. 1c). This sequence is based on previous reports on cationic amphipathic peptides possessing both hydrophobic and hydrophilic regions, known to enable binding to the microbial cell envelope.^20^ Compound 8 proved compatible with standard SPPS coupling agents (*e.g.*, Pyoxim) and cleavage conditions (*e.g.*, trifluoroacetic acid, TFA), and we could successfully synthesize the labeled peptide 32 (Fig. 1c) in 4% yield and with high purities over 95%. Next, we exploited the turn-on features of peptide 32 for wash-free imaging of bacterial cells (Fig. 1d). First, we tested different concentrations of peptide 32 for imaging Gram-positive (*S. aureus*) and Gram-negative (*E. coli*) bacteria under fluorescence microscopy (Fig. S1†). Importantly, the environmental sensitivity of the NBD moiety allowed direct visualization of both bacterial cells without any additional steps or washing procedures (Fig. S2†). After optimization of the labeling protocol, we employed peptide 32 in confocal fluorescence microscopy for real-time and wash-free imaging of *S. aureus* and *E. coli* bacterial cultures with high signal-to-noise ratios (Fig. 1e). Altogether, these results confirm that the NBD moiety did not alter the bacterial-targeting ability of the peptide sequence and showcase the utility of this fluorescence labeling method for NIR wash-free imaging in live cells.

## Conclusions

In summary, we have developed an unprecedent late-stage labeling strategy of structurally complex peptides with NIR-emitting dyes *via* C(sp^2^)–H activation. Thereby, the assembly of fluorescent amino acids and peptides was achieved with excellent site-, chemo-, and diastereo-*E*-selectivity using an earth-abundant manganese(i) pre-catalyst of low toxicity. Furthermore, we identified that the NIR-labeled peptide 32 can be used for imaging of live bacteria and under wash-free conditions. Overall, this strategy features excellent properties of NBD labeled amino acids and peptides, such as large Stokes shifts, NIR emission wavelengths, and excellent fluorogenicity for real time imaging in live cells.

## Data availability

Data available from the authors upon reasonable request.

## Author contributions

T. O., and L. A. conceived the project. T. O. and A. K. performed the synthetic experiments. L. M.-T. and V. C. performed imaging experiments. T. O., L. M.-T., M. V., and L. A. wrote the manuscript with contributions from all authors.

## Conflicts of interest

There are no conflicts to declare.

## Supplementary Material

SC-014-D3SC01868G-s001
