# Supplementary material for: Late-stage peptide labeling with near-infrared fluorogenic nitrobenzodiazoles by manganese-catalyzed C–H activation
Source: Chem Sci. 2023 May 4;14(21):5728–33. doi: 10.1039/d3sc01868g (PMC10231426; doi:10.1039/d3sc01868g)
Supplement: SC-014-D3SC01868G-s001 [file SC-014-D3SC01868G-s001.pdf]

Electronic Supplementary Material (ESI) for Chemical Science.
This journal is © The Royal Society of Chemistry 2023

Late-Stage Peptide Labeling with Near-Infrared Fluorogenic Nitrobenzodiazoles by Manganese-Catalyzed C–H Activation

T. Oyama‡, L. Mendive-Tapia‡, V. Cowell, A. Kopp, M. Vendrell* and L. Ackermann*

Table of Contents

**General Remarks**	**S3**
**General Procedure**	**S3**
**Optimization Studies**	**S4**
**Fluorescence and Brightfield microscopy Images**	**S5**
**Experimental Procedures**	**S6**
**Computational Studies**	**S26**
**Supplementary References**	**S32**
**^{1}H NMR and ^{13}C NMR Spectra**	**S33**

General Remarks

Catalytic reactions were performed under a N_2 atmosphere using pre-dried glassware and standard Schlenk techniques. 1,2-dichloroethane (DCE) was dried over CaH_2 and fleshly distilled under N_2. Otherwise stated, peptides were synthesized under standard solution phase protocols (EDCI/HOBt) according to previously described methods.[1] Other chemicals were obtained from commercial sources and were used without further purification. Yields refer to isolated compounds, estimated to be >95% pure as determined by ^{1}H NMR. **Flash chromatography**: Merck silica gel 60 (40–63 µm). **NMR**: Spectra were recorded on a Varian Mercury Vx 300, Varian VNMRS 300, Varian Inova 500, Varian Inova 600, Bruker Avance III 400, Bruker Avance III HD 400 and a Bruker Avance III HD 500 instrument in the solvent indicated; chemical shifts (δ) are provided in ppm. **IR**: All spectra were recorded on a Bruker FT-IR Alpha device. **MS**: HPLC-MS analysis was recorded on HPLC Agilent 1200 System comprising a Kinetex C18 column (5 µm, 100 Å, 150 x 4.6 mm) and a MS detector configured with an electrospray ionization source (6110 quadrupole LC/MS). ESI-MS was recorded on Bruker Daltonic micrOTOF. High resolution mass spectrometry (HR-MS) was recorded on micrOTOF, Bruker Daltonic. MALDI analysis was recorded on Bruker UltrafleXtreme MALDI TOF-TOF. Purification of compound **32** was conducted in a semi-Preparative Agilent HPLC consisting of a 1220 Infinity II autosampler and a 1260 Infinity II detector. **Melting points (M.p.)**: All compounds were measured on Stuart™ melting point apparatus SMP3, and the values are uncorrected. **Spectroscopy measurements**: Spectral properties were recorded in 96-well plates on a BioTek Cytation 3 spectrophotometer. Compounds were dissolved at the indicated concentrations and spectra were recorded at room temperature. Quantum yields were referenced to fluorescein in basic EtOH.[2] Values were obtained as means from three independent experiments.

General Procedure of Manganese-Catalyzed C–H alkenylation of Amino acids and Peptides

General Procedure A: A suspension of amino acid or peptide (0.10 mmol, 1.0 equiv), NBD-alkyne (0.15 mmol, 1.5 equiv), $MnBr(CO)_5$ (5.5 mg, 20 mol %), BPh_3 (9.7 mg, 40 mol %) and KOAc (3.9 mg, 40 mol %) in 1,2-dichloroethane (1.0 mL) was stirred at 60 °C for 18 h under N_2 atmosphere. After cooling to ambient temperature, the mixture was concentrated *in vacuo*. Purification by column chromatography on silica gel afforded the desired products.

General Procedure B: A suspension of peptide (0.050 mmol, 1.0 equiv), NBD-alkyne (0.15 mmol, 3.0 equiv), $MnBr(CO)_5$ (13.7 mg, 100 mol %), BPh_3 (24.2 mg, 200 mol %) and KOAc (9.9 mg, 200 mol %) in 1,2-dichloroethane (1.0 mL) was stirred at 60 °C for 6 h under N_2 atmosphere. After cooling to ambient temperature, the mixture was concentrated *in vacuo*. Purification by column chromatography on silica gel afforded the desired products.

General Procedure C: A suspension of peptide (0.050 mmol, 1.0 equiv), NBD-alkyne (0.10 mmol, 2.0 equiv), $MnBr(CO)_5$ (5.5 mg, 40 mol %), BPh_3 (9.7 mg, 80 mol %) and KOAc (3.9 mg, 80 mol %) in 1,2-dichloroethane (1.0 mL) was stirred at 60 °C for 18 h under N_2 atmosphere. After cooling to ambient temperature, the mixture was concentrated *in vacuo*. Purification by column chromatography on silica gel afforded the desired products.

Optimization studies

Table S1. Optimization of the reaction conditions.

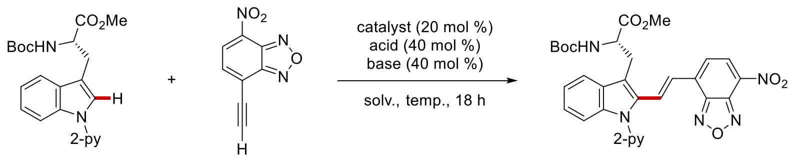

1 (1.0 equiv)   **2** (1.5 equiv)   **3**

entry	catalyst	acid	base	solvent	temperature (°C)	yield (%) [a]
1	$MnBr(CO)_5$	1-Ad-CO_2H	–	1,4-dioxane	100	17
2	$MnBr(CO)_5$	Ph-CO_2H	DIEPA	Et_2O	80	17 [b]
3	$MnBr(CO)_5$	Ph-CO_2H	–	1,4-dioxane	80	15 [b]
4	$MnBr(CO)_5$	Ph-CO_2H	KOAc	1,4-dioxane	80	10 [b]
5	$MnBr(CO)_5$	BPh_3	KOAc	1,4-dioxane	80	15 [b]
6	$MnBr(CO)_5$	BPh_3	KOAc	DCE	80	86
7	$MnBr(CO)_5$	BPh_3	KOAc	DCE	60	95
8	$MnBr(CO)_5$	BPh_3	KOAc	DCE	60	87 [c]
9	$MnBr(CO)_5$	BPh_3	KOAc	DCE	37	78
10	$MnBr(CO)_5$	BPh_3	–	DCE	60	trace
11	$MnBr(CO)_5$	–	KOAc	DCE	60	4 [b]
12	–	BPh_3	KOAc	DCE	60	–
13	$MnBr(CO)_5$	BPh_3	NaOAc	DCE	60	21
14	$MnBr(CO)_5$	BPh_3	KOAc	DCE/DMF (4/1)	60	trace
15	$MnBr(CO)_5$	BPh_3	KOAc	DCE/*i*-PrOH (4/1)	60	12 [b]
16	$MnBr(CO)_5$	BPh_3	KOAc	DCE/toluene (4/1)	60	97 [b]
17	$MnBr(CO)_5$	BPh_3	KOAc	DCE/H_2O (4/1)	60	15 [b]
18	$Mn_2(CO)_{10}$	BPh_3	KOAc	DCE	60	trace
19	$ReBr(CO)_5$	BPh_3	KOAc	DCE	60	60
20	$Pd(OAc)_2$	BPh_3	KOAc	DCE	60	–
21	$[RuCl_2$(*p*-cymene)$]_2$ [d]	BPh_3	KOAc	DCE	60	–
22	$[RhCp^*Cl_2]_2$	BPh_3	KOAc	DCE	60	–
23	$Co(OAc)_2$	BPh_3	KOAc	DCE	60	–

Reaction conditions: 0.10 mmol of **1**, 0.15 mmol of **2**, 20 mol % of catalyst, 40 mol % of base, 40 mol % of acid and 1.0 mL of solvent were used. [a] Isolated yields. [b] Determined by ^1H NMR (Ph_3CH was used as internal standard). [c] 10 mol % of catalyst, 20 mol % of acid and base were used. [d] 10 mol % of catalyst was used.

Fluorescence and Brightfield microscopy Images

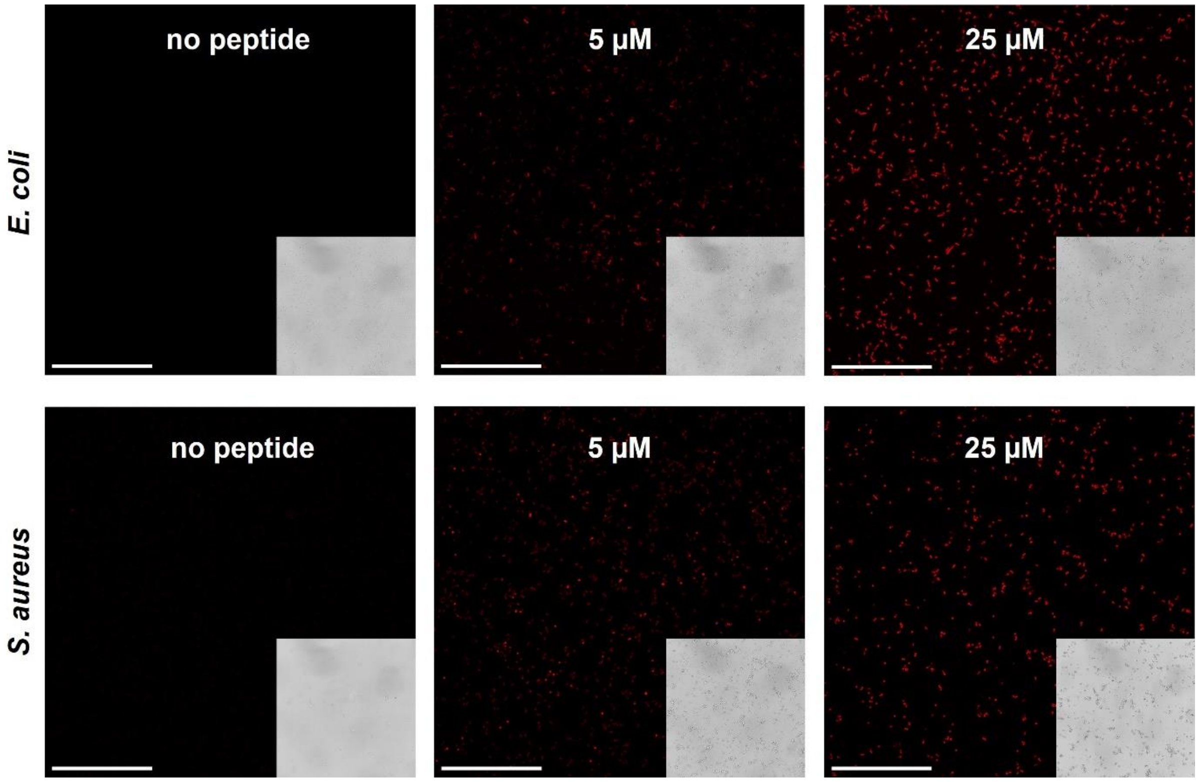

Figure S1. Representative fluorescence and brightfield microscopy images of Gram-negative bacteria (top panel, *E. coli*) and Gram-positive bacteria (bottom panel, *S. aureus*) after staining with different concentrations of peptide **32** (red). Scale bar 50 µm.

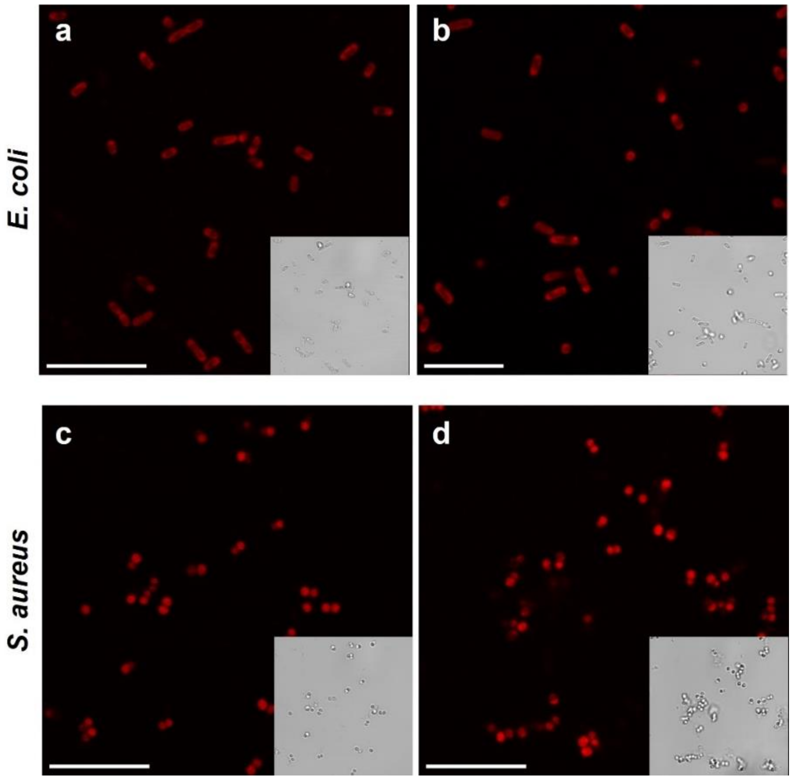

Figure S2. Fluorescence and brightfield microscopy images of Gram-negative bacteria (top panel, *E. coli*) and Gram-positive bacteria (bottom panel, *S. aureus*) after labeling with compound **32** (25 µM) with addition of one washing step (b and d panels) or without any washing steps (a and c panels). Scale bar 10 µm.

Experimental Procedures

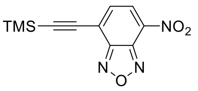

4-nitro-7-((trimethylsilyl)ethynyl)benzo[*c*][1,2,5]oxadiazole (S-1)

To the mixture of 4-chloro-7-nitrobenzo[*c*][1,2,5]oxadiazole (1.0 g, 5.0 mmol), $PdCl_2(PPh_3)_2$ (105 mg, 3.0 mol %), and CuI (48 mg, 5.0 mol %) were added THF (50 mL), Et_3N (3.5 mL, 5.0 equiv), and trimethylsilylacetylene (0.69 mL, 1.0 equiv) at room temperature. After stirring for 1 h at this temperature, the reaction mixture was filtered through Celite pad and washed with EtOAc. The filtrate was concentrated in vacuo and the residue was purified by flash column chromatography (silica gel, *n*-hexane/DCM = 3/1) to afford TMS-NBD-alkyne **S-1** (857 mg, 66%) as a yellow solid.

M.p.: 108–110 °C.

^{1}H NMR (300 MHz, $CDCl_3$): δ 8.46 (d, *J* = 7.6 Hz, 1H), 7.67 (d, *J* = 7.6 Hz, 1H), 0.34 (s, 9H).

^{13}C NMR (75 MHz, $CDCl_3$): δ 150.9 (C_q), 142.6 (C_q), 136.0 (C_q), 132.8 (CH), 130.2 (CH), 121.2 (C_q), 112.3 (C_q), 97.2 (C_q), –0.41(CH_3, overlapped, 3C).

IR (ATR): 2962, 1537, 1336, 1251, 846, 816, 734 cm^{-1}.

MS (ESI) *m/z* (relative intensity): 284 $[M+Na]^+$ (100).

HR-MS (ESI) *m/z* calcd for $C_{11}H_{11}N_3O_3Si$ $[M+Na]^+$: 284.0462, found: 284.0459.

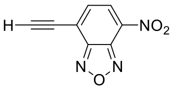

4-ethynyl-7-nitrobenzo[*c*][1,2,5]oxadiazole (2)

To the mixture of **S-1** (784 mg, 3.00 mmol) in MeOH (30 mL) was added KF (174 mg, 1.0 equiv) at 0 °C. After stirring for 30 minutes at this temperature, the reaction mixture was quenched by adding saturated aqueous NH_4Cl. The mixture was extracted with Et_2O (x2). The combined organic extracts were washed with brine, dried over Na_2SO_4, and concentrated in vacuo. The residue was purified by flash column chromatography (silica gel, *n*-hexane/EtOAc = 3/1) to afford NBD-alkyne **2** (323 mg, 57%) as a brown solid.

M.p.: 118–119 °C.

^{1}H NMR (300 MHz, $CDCl_3$): δ 8.49 (d, *J* = 7.6 Hz, 1H), 7.76 (d, *J* = 7.6 Hz, 1H), 3.97 (s, 1H).

^{13}C NMR (75 MHz, $CDCl_3$): δ 151.0 (C_q), 142.5 (C_q), 136.6 (C_q), 133.7 (CH), 130.0 (CH), 120.0 (C_q), 91.4 (CH), 76.7 (C_q).

IR (ATR): 3261, 3098, 2109, 1527, 1445, 1374, 1343, 1068, 995, 893, 868, 814, 734, 682 cm^{-1}.

MS (ESI) *m/z* (relative intensity): 188 $[M+H]^+$ (50), 220 $[M+MeOH]^+$ (100).

HR-MS (ESI) *m/z* calcd for $C_8H_3N_3O_3$ $[M+H]^+$: 188.0102, found: 188.0105.

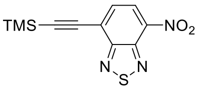

4-nitro-7-((trimethylsilyl)ethynyl)benzo[*c*][1,2,5]thiadiazole (S-2)

To the mixture of 4-bromo-7-nitrobenzo[*c*][1,2,5]thiadiazole (260 mg, 1.0 mmol), $PdCl_2(PPh_3)_2$ (21 mg, 3.0 mol %), and CuI (9.5 mg, 5.0 mol %) were added THF (10 mL), Et_3N (0.70 mL, 5.0 equiv), and trimethylsilylacetylene (0.14 mL, 1.0 equiv) at room temperature. After stirring for 1 h at 60 °C, the reaction mixture was filtered through Celite pad and washed with EtOAc. The filtrate was concentrated in vacuo and the residue was purified by flash column chromatography (silica gel, *n*-hexane/DCM = 3/1) to afford TMS-NBD(S)-alkyne **S-2** (238 mg, 86%) as a yellow solid.

M.p.: 126–128 °C.

^{1}H NMR (300 MHz, $CDCl_3$): δ 8.53 (d, *J* = 7.9 Hz, 1H), 7.85 (d, *J* = 7.9 Hz, 1H), 0.34 (s, 9H).

^{13}C NMR (75 MHz, $CDCl_3$): δ 156.0 (C_q), 146.3 (C_q), 139.1 (C_q), 131.3 (CH), 127.3 (CH), 124.2 (C_q), 108.9 (C_q), 99.0 (C_q), –0.22 (CH_3, overlapped, 3C).

IR (ATR): 2959, 2258, 1525, 1330, 1250, 908, 843, 820, 731 cm^{-1}.

MS (ESI) *m/z* (relative intensity): 300 $[M+Na]^+$ (100).

HR-MS (ESI) *m/z* calcd for $C_{11}H_{11}N_3O_2SSi$ $[M+Na]^+$: 300.0233, found: 300.0232.

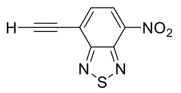

4-ethynyl-7-nitrobenzo[*c*][1,2,5]thiadiazole (S-3)

To the mixture of **S-2** (238 mg, 0.858 mmol) in MeOH (8.6 mL) was added KF (50 mg, 1.0 equiv) at 0 °C. After stirring for 30 minutes at room temperature, the reaction mixture was quenched by adding saturated aqueous NH_4Cl. The mixture was extracted with Et_2O (x2). The combined organic extracts were washed with brine, dried over Na_2SO_4, and concentrated in vacuo. The residue was purified by flash column chromatography (silica gel, *n*-hexane/EtOAc = 2/1) to afford NBD(S)-alkyne **S-3** (151 mg, 86%) as a brown solid.

M.p.: 90–95 °C.

^{1}H NMR (400 MHz, $CDCl_3$): δ 8.56 (d, *J* = 7.8 Hz, 1H), 7.93 (d, *J* = 7.8 Hz, 1H), 3.89 (s, 1H).

^{13}C NMR (101 MHz, $CDCl_3$): δ 156.3 (C_q), 146.3 (C_q), 139.7 (C_q), 131.8 (CH), 127.1 (CH), 123.1 (C_q), 88.9 (CH), 78.3 (C_q).

IR (ATR): 3279, 2111, 1530, 1509, 1388, 1348, 1336, 1300, 1273, 1049, 869, 822, 674 cm^{-1}.

MS (ESI) *m/z* (relative intensity): 204 $[M+H]^+$ (100).

HR-MS (ESI) *m/z* calcd for $C_8H_3N_3O_2S$ $[M+H]^+$: 203.9873, found: 203.9887.

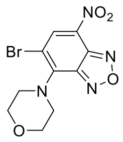

5-bromo-4-morpholino-7-nitrobenzo[*c*][1,2,5]oxadiazole (S-4)

To the mixture of 4-morpholino-7-nitrobenzo[c][1,2,5]oxadiazole (446 mg, 1.78 mmol) in CH_3CN (9.0 mL) was added *N*-bromosuccinimide (380 mg, 1.2 equiv) at room temperature. After stirring for 4 h at 60 °C, the reaction mixture was quenched by adding aqueous $Na_2S_2O_8$. The mixture was extracted with DCM (x3). The combined organic extracts were dried over Na_2SO_4 and concentrated in vacuo. The residue was purified by recrystallization with DCM to afford NBD-Br **S-4** (346 mg, 59%) as a red solid.

M.p.: 155–157 °C.

^{1}H NMR (400 MHz, $CDCl_3$): δ 8.59 (s, 1H), 3.94 (s, 8H).

^{13}C NMR (101 MHz, $CDCl_3$): δ 147.7 (C_q), 144.5 (C_q), 143.6 (C_q), 139.7 (CH), 127.3 (C_q), 103.7 (C_q), 67.2 (CH_2, overlapped, 2C), 52.4 (CH_2, overlapped, 2C).

IR (ATR): 2904, 2858, 1715, 1610, 1510, 1437, 1341, 1296, 1258, 1177, 1109, 1053, 1000, 892, 818 cm^{-1}.

MS (ESI) *m/z* (relative intensity): 301 $[M+H]^+$ (99) (^{79}Br), 303 $[M+H]^+$ (100) (^{81}Br).

HR-MS (ESI) *m/z* calcd for $C_{10}H_9N_4O_4{}^{79}Br$ $[M+H]^+$: 300.9578, found: 300.9577.

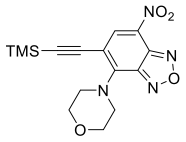

4-morpholino-7-nitro-5-((trimethylsilyl)ethynyl)benzo[*c*][1,2,5]oxadiazole (S-5)

To the mixture of **S-4** (66 mg, 0.20 mmol), $PdCl_2(PPh_3)_2$ (7.0 mg, 5.0 mol %), and CuI (3.8 mg, 10 mol %) were added THF (2.0 mL), Et_3N (0.14 mL, 5.0 equiv), and trimethylsilylacetylene (33 μL, 1.2 equiv) at room temperature. After stirring for 4 h at 60 °C, the reaction mixture was filtered through Celite pad and washed with EtOAc. The filtrate was concentrated in vacuo and the residue was purified by flash column chromatography (silica gel, *n*-hexane/EtOAc = 3/1) to afford TMS-NBD-alkyne **S-5** (45.4 mg, 66%) as a red solid.

M.p.: 147–149 °C.

^{1}H NMR (300 MHz, $CDCl_3$): δ 8.47 (s, 1H), 4.27 (dd, *J* = 4.9, 4.5 Hz, 4H), 3.93 (dd, *J* = 4.9, 4.5 Hz, 4H), 0.27 (s, 9H).

^{13}C NMR (75 MHz, $CDCl_3$): δ 146.7 (C_q), 146.6 (C_q), 143.8 (C_q), 140.5 (CH), 125.3 (C_q), 104.4 (C_q), 101.9 (C_q), 101.4 (C_q), 67.4 (CH_2, overlapped, 2C), 52.6 (CH_2, overlapped, 2C), –0.21 (CH_3, overlapped, 3C).

IR (ATR): 2961, 2859, 2149, 1544, 1524, 1342, 1274, 1115, 1034, 881, 843 cm^{-1}.

MS (ESI) *m/z* (relative intensity): 347 $[M+H]^+$ (5), 369 $[M+Na]^+$ (100).

HR-MS (ESI) *m/z* calcd for $C_{15}H_{18}N_4O_4Si$ $[M+Na]^+$: 369.0990, found: 369.0982.

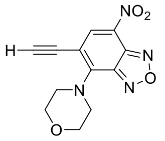

5-ethynyl-4-morpholino-7-nitrobenzo[*c*][1,2,5]oxadiazole (S-6)

To the mixture of **S-5** (83 mg, 0.24 mmol) in MeOH (2.4 mL) was added KF (15 mg, 1.0 equiv) at 0 °C. After stirring for 1 h at room temperature, the reaction mixture was quenched by adding saturated aqueous NH_4Cl. The mixture was extracted with Et_2O (x2). The combined organic extracts were washed with brine, dried over Na_2SO_4, and concentrated in vacuo. The residue was purified by flash column chromatography (silica gel, *n*-hexane/DCM/EtOAc = 2/1/1) to afford NBD-alkyne **S-6** (48.5 mg, 74%) as a red solid.

M.p.: 190–193 °C.

^{1}H NMR (300 MHz, $CDCl_3$): δ 8.54 (s, 1H), 4.36 – 4.23 (m, 4H), 4.04 – 3.90 (m, 4H), 3.62 (s, 1H).

^{13}C NMR (101 MHz, Acetone-d_6): δ 148.7 (C_q), 148.1 (C_q), 145.0 (C_q), 141.2 (CH), 125.7 (C_q), 100.9 (C_q), 87.7 (CH), 81.4 (C_q), 67.8 (CH_2, overlapped, 2C), 53.6 (CH_2, overlapped, 2C).

IR (ATR): 3241, 3027, 2190, 1611, 1550, 1524, 1508, 1355, 1325, 1298, 1264, 1115, 1035, 1002, 874 cm^{-1}.

MS (ESI) *m/z* (relative intensity): 297 $[M+Na]^+$ (90), 329 $[M+MeOH+Na]^+$ (100).

HR-MS (ESI) *m/z* calcd for $C_{12}H_{10}N_4O_4$ $[M+Na]^+$: 297.0594, found: 297.0592.

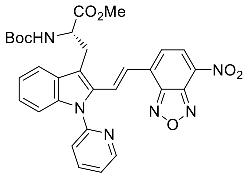

methyl (*S*,*E*)-2-((*tert*-butoxycarbonyl)amino)-3-(2-(2-(7-nitrobenzo[*c*][1,2,5]oxadiazol-4-yl)vinyl)-1-(pyridin-2-yl)-1*H*-indol-3-yl)propanoate (3)

The general procedure **A** was followed using methyl N_α-(*tert*-butoxycarbonyl)-1-(pyridin-2-yl)-*L*-tryptophanate (**1a**) (39.5 mg, 0.10 mmol), NBD-alkyne **2** (28.4 mg, 0.15 mmol), $MnBr(CO)_5$ (5.5 mg, 20 mol %), BPh_3 (9.7 mg, 40 mol %) and KOAc (3.9 mg, 40 mol %) in DCE (1.0 mL). Purification by column chromatography on silica gel (*n*-hexane/EtOAc = 3/2) yielded **3** (55.5 mg, 95%) as a purple solid.

M.p.: 185–187 °C.

^{1}H NMR (300 MHz, $CDCl_3$): δ 8.75 (dd, *J* = 5.3, 1.7 Hz, 1H), 8.43 (d, *J* = 16.5 Hz, 1H), 8.38 (d, *J* = 8.1 Hz, 1H), 7.97 (td, *J* = 7.7, 2.0 Hz, 1H), 7.59 (d, *J* = 7.8 Hz, 1H), 7.53 – 7.35 (m, 4H), 7.27 (t, *J* = 7.0 Hz, 1H), 7.20 (t, *J* = 7.1 Hz, 1H), 7.04 (d, *J* = 16.5 Hz, 1H), 5.36 (d, *J* = 8.3 Hz, 1H), 4.73 (q, *J* = 7.8 Hz, 1H), 3.60 (dd, *J* = 14.5, 5.7 Hz, 1H), 3.54 (dd, *J* = 14.5, 8.0 Hz, 1H), 3.49 (s, 3H), 1.39 (s, 9H).

^{13}C NMR (75 MHz, $CDCl_3$): δ 172.6 (C_q), 155.1 (C_q), 151.4 (C_q), 150.1 (CH), 148.5 (C_q), 143.5 (C_q), 139.0 (C_q), 138.9 (CH), 136.3 (C_q), 133.8 (C_q), 131.6 (CH), 129.9 (CH), 129.0 (C_q), 126.6 (CH), 125.8 (CH), 124.3 (CH), 123.1 (CH), 122.4 (CH), 121.9 (CH), 119.8 (CH), 118.0 (C_q), 111.2 (CH), 80.2 (C_q), 54.4 (CH), 52.6 (CH_3), 29.7 (CH_2), 28.4 (CH_3, overlapped, 3C). (One aromatic C_q is missing due to overlap.)

IR (ATR): 3383, 2976, 1743, 1707, 1515, 1437, 1317, 1158, 997, 744 cm^{-1}.

MS (ESI) *m/z* (relative intensity): 607 $[M+Na]^+$ (100).

HR-MS (ESI) *m/z* calcd for $C_{30}H_{28}N_6O_7$ $[M+Na]^+$: 607.1912, found: 607.1905.

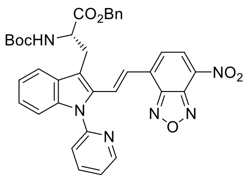

benzyl (*S,E*)-2-((*tert*-butoxycarbonyl)amino)-3-(2-(2-(7-nitrobenzo[*c*][1,2,5]oxadiazol-4-yl)vinyl)-1-(pyridin-2-yl)-1*H*-indol-3-yl)propanoate (4)

The general procedure **A** was followed using benzyl N_α-(*tert*-butoxycarbonyl)-1-(pyridin-2-yl)-*L*-tryptophanate (**1b**) (47.2 mg, 0.10 mmol), NBD-alkyne **2** (28.4 mg, 0.15 mmol), $MnBr(CO)_5$ (5.5 mg, 20 mol %), BPh_3 (9.7 mg, 40 mol %) and KOAc (3.9 mg, 40 mol %) in DCE (1.0 mL). Purification by column chromatography on silica gel (*n*-hexane/EtOAc = 2/1) yielded **4** (47.6 mg, 72%) as a purple solid.

M.p.: 115–117 °C.

^{1}H NMR (300 MHz, $CDCl_3$): δ 8.75 (dd, *J* = 5.0, 1.8 Hz, 1H), 8.40 (d, *J* = 16.5 Hz, 1H), 8.32 (d, *J* = 7.8 Hz, 1H), 7.94 (td, *J* = 7.7, 2.0 Hz, 1H), 7.62 (d, *J* = 7.8 Hz, 1H), 7.46 (dd, *J* = 7.5, 4.9 Hz, 1H), 7.40 (m, 2H), 7.33 (d, *J* = 8.0 Hz, 1H), 7.30 – 7.18 (m, 5H), 7.13 (d, *J* = 16.5 Hz, 1H), 6.94 (brd, *J* = 6.2 Hz, 2H), 5.40 (d, *J* = 8.2 Hz, 1H), 5.03 (d, *J* = 12.2 Hz, 1H), 4.81 – 4.76 (m, 1H), 4.73 (d, *J* = 12.2 Hz, 1H), 3.63 (dd, *J* = 14.4, 6.2 Hz, 1H), 3.53 (dd, *J* = 14.2, 8.1 Hz, 1H), 1.39 (s, 9H).

^{13}C NMR (75 MHz, $CDCl_3$): δ 172.2 (C_q), 155.1 (C_q), 151.2 (C_q), 150.1 (CH), 148.5 (C_q), 143.4 (C_q), 138.0 (CH), 136.4 (C_q), 134.8 (C_q), 133.7 (C_q), 131.6 (CH), 130.1 (CH), 129.1 (C_q), 128.6 (CH), 128.4 (C_q), 128.3(CH), 126.4 (CH), 125.7 (CH), 124.4 (CH), 123.1 (CH), 122.3 (CH), 121.9 (CH), 119.7 (CH), 117.5 (C_q), 111.2 (CH), 80.3 (C_q), 67.8 (CH_2), 54.4 (CH), 29.9 (CH_2), 28.4 (CH_3, overlapped, 3C). (Three aromatic CH and one aromatic C_q are missing due to overlap.)

IR (ATR): 3433, 2977, 1702, 1513, 1437, 1312, 1155, 732 cm^{-1}.

MS (ESI) *m/z* (relative intensity): 661 $[M+H]^+$ (25), 683 $[M+Na]^+$ (100).

HR-MS (ESI) *m/z* calcd for $C_{36}H_{32}N_6O_7$ $[M+H]^+$: 661.2405, found: 661.2398.

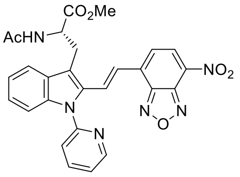

methyl (*S,E*)-2-acetamido-3-(2-(2-(7-nitrobenzo[*c*][1,2,5]oxadiazol-4-yl)vinyl)-1-(pyridin-2-yl)-1*H*-indol-3-yl)propanoate (5)

The general procedure **A** was followed using methyl N_α-acetyl-1-(pyridin-2-yl)-*L*-tryptophanate (**1c**) (33.7 mg, 0.10 mmol), NBD-alkyne **2** (28.4 mg, 0.15 mmol), $MnBr(CO)_5$ (5.5 mg, 20 mol %), BPh_3 (9.7 mg, 40 mol %) and KOAc (3.9 mg, 40 mol %) in DCE (1.0 mL). Purification by column chromatography on silica gel (*n*-hexane/EtOAc = 1/1 → EtOAc) yielded **5** (49.0 mg, 93%) as a purple solid.

M.p.: 216–218 °C.

^{1}H NMR (300 MHz, $CDCl_3$): δ 8.76 (d, *J* = 4.0 Hz, 1H), 8.45 (d, *J* = 7.9 Hz, 1H), 8.41 (d, *J* = 16.7 Hz, 1H), 7.98 (ddd, *J* = 7.7, 7.7, 2.0 Hz, 1H), 7.60 (d, *J* = 7.9 Hz, 1H), 7.58 (d, *J* = 8.6 Hz, 1H), 7.51 – 7.36 (m, 3H), 7.32 – 7.18 (m, 2H), 7.15 (d, *J* = 16.7 Hz, 1H), 6.31 (d, *J* = 7.7 Hz, 1H), 4.99 (ddd, *J* = 8.5, 7.7, 5.1 Hz, 1H), 3.69 (dd, *J* = 14.4, 5.1 Hz, 1H), 3.54 (dd, *J* = 14.4, 8.5 Hz, 1H), 3.46 (s, 3H), 1.98 (s, 3H).

^{13}C NMR (75 MHz, $CDCl_3$): δ 172.4 (C_q), 169.9 (C_q), 151.1 (C_q), 150.2 (CH), 148.2 (C_q), 143.5 (C_q), 138.9 (CH), 138.9 (C_q), 136.1 (C_q), 133.9 (C_q), 133.9 (C_q), 131.7 (CH), 129.7 (CH), 129.0 (C_q), 126.8 (CH), 125.7 (CH), 124.3 (CH), 123.2 (CH), 122.4 (CH), 121.8 (CH), 119.5 (CH), 117.3 (C_q), 111.2 (CH), 53.1 (CH), 52.7 (CH_3), 29.1 (CH_2), 23.4 (CH_3).

IR (ATR): 3055, 1743, 1617, 1438, 1319, 1088, 746 cm^{-1}.

MS (ESI) *m/z* (relative intensity): 527 $[M+H]^+$ (5), 549 $[M+Na]^+$ (100).

HR-MS (ESI) *m/z* calcd for $C_{27}H_{22}N_6O_6$ $[M+Na]^+$: 549.1493, found: 549.1477.

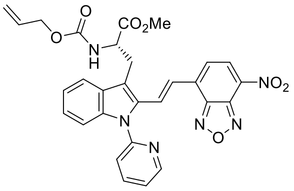

methyl (*S,E*)-2-(((allyloxy)carbonyl)amino)-3-(2-(2-(7-nitrobenzo[*c*][1,2,5]oxadiazol-4-yl)vinyl)-1-(pyridin-2-yl)-1*H*-indol-3-yl)propanoate (6)

The general procedure **A** was followed using methyl N_α-((allyloxy)carbonyl)-1-(pyridin-2-yl)-*L*-tryptophanate (**1d**) (37.9 mg, 0.10 mmol), NBD-alkyne **2** (28.4 mg, 0.15 mmol), $MnBr(CO)_5$ (5.5 mg, 20 mol %), BPh_3 (9.7 mg, 40 mol %) and KOAc (3.9 mg, 40 mol %) in DCE

(1.0 mL). Purification by column chromatography on silica gel (*n*-hexane/DCM/MeOH = 90/10/1) yielded **6** (42.6 mg, 75%) as a purple solid.

M.p.: 187–189 °C.

^{1}H NMR (300 MHz, $CDCl_3$): δ 8.76 (dd, *J* = 5.0, 1.9 Hz, 1H), 8.48 – 8.35 (m, 2H), 7.97 (td, *J* = 7.7, 2.0 Hz, 1H), 7.60 (d, *J* = 7.8 Hz, 1H), 7.50 – 7.35 (m, 4H), 7.28 (t, *J* = 7.1 Hz, 1H), 7.21 (t, *J* = 7.6 Hz, 1H), 6.96 (d, *J* = 16.5 Hz, 1H), 5.79 (ddt, *J* = 16.3, 10.7, 5.6 Hz, 1H), 5.55 (d, *J* = 8.2 Hz, 1H), 5.20 (d, *J* = 16.3 Hz, 1H), 5.12 (dd, J = 10.7 Hz, 1H), 4.80 (q, *J* = 7.6 Hz, 1H), 4.53 (dd, *J* = 13.6, 5.6 Hz, 1H), 4.45 (dd, J = 13.6, 5.6 Hz, 1H), 3.66 (dd, *J* = 14.5, 5.6 Hz, 1H), 3.56 (dd, *J* = 14.5, 7.7 Hz, 1H), 3.51 (s, 3H).

^{13}C NMR (75 MHz, $CDCl_3$): δ 172.2 (C_q), 155.6 (C_q), 151.4 (C_q), 150.2 (CH), 148.5 (C_q), 143.5 (C_q), 139.2 (C_q), 138.9 (CH), 136.2 (C_q), 133.9 (C_q), 132.5 (CH), 131.6 (CH), 129.9 (CH), 128.9 (C_q), 126.6 (CH), 125.9 (CH), 124.3 (CH), 123.2 (CH), 122.4 (CH), 121.9 (CH), 119.7 (CH), 117.9 (CH_2), 117.7 (C_q), 111.2 (CH), 66.0 (CH_2), 54.7 (CH), 52.8 (CH_3), 29.4 (CH_2). (One aromatic C_q is missing due to overlap.)

IR (ATR): 3313, 2924, 1746, 1712, 1518, 1320, 1154 cm^{-1}.

MS (ESI) *m/z* (relative intensity): 569 $[M+H]^+$ (100).

HR-MS (ESI) *m/z* calcd for $C_{29}H_{24}N_6O_7$ $[M+H]^+$: 569.1779, found: 569.1770.

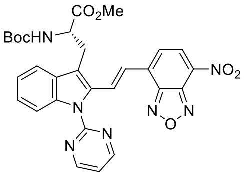

methyl (*S*,*E*)-2-((*tert*-butoxycarbonyl)amino)-3-(2-(2-(7-nitrobenzo[*c*][1,2,5]oxadiazol-4-yl)vinyl)-1-(pyrimidin-2-yl)-1*H*-indol-3-yl)propanoate (7)

The general procedure **A** was followed using methyl N_α-(*tert*-butoxycarbonyl)-1-(pyrimidin-2-yl)-*L*-tryptophanate (**1e**) (39.6 mg, 0.10 mmol), NBD-alkyne **2** (28.4 mg, 0.15 mmol), $MnBr(CO)_5$ (5.5 mg, 20 mol %), BPh_3 (9.7 mg, 40 mol %) and KOAc (3.9 mg, 40 mol %) in DCE (1.0 mL). Purification by column chromatography on silica gel (DCM/EtOAc = 4/1) yielded **7** (52.1 mg, 89%) as a red solid.

M.p.: 219–221 °C.

^{1}H NMR (300 MHz, $CDCl_3$): δ 8.90 – 8.77 (m, 3H), 8.48 (d, *J* = 7.7 Hz, 1H), 8.30 (d, *J* = 8.3 Hz, 1H), 7.76 (d, *J* = 7.8 Hz, 1H), 7.57 (d, *J* = 7.8 Hz, 1H), 7.46 (d, *J* = 16.6 Hz, 1H), 7.36 (t, *J* = 7.9 Hz, 1H), 7.33 – 7.23 (m, 2H), 5.40 (d, *J* = 8.3 Hz, 1H), 4.73 (q, *J* = 7.7 Hz, 1H), 3.59 (dd, *J* = 14.4, 5.6 Hz, 1H), 3.45 (dd, *J* = 14.4, 8.9 Hz, 1H), 3.43 (s, 3H), 1.43 (s, 9H).

^{13}C NMR (75 MHz, $CDCl_3$): δ 172.8 (C_q), 158.6 (CH), 157.9 (C_q), 155.1 (C_q), 148.7 (C_q), 143.6 (C_q), 137.4 (C_q), 136.8 (C_q), 134.1 (C_q), 133.0 (CH), 131.7 (CH), 130.0 (C_q), 127.5 (CH), 126.0 (CH), 124.4 (CH), 122.8 (CH), 119.4 (CH), 118.3 (C_q), 118.0 (CH), 114.2 (CH), 80.4 (C_q), 54.3 (CH), 52.6 (CH_3), 30.3 (CH_2), 28.5 (CH_3, overlapped, 3C). (One aromatic C_q and one aromatic CH are missing due to overlap.)

IR (ATR): 3407, 2976, 1741, 1708, 1519, 1422, 1322, 1160 cm^{-1}.

MS (ESI) *m/z* (relative intensity): 608 $[M+Na]^+$ (100).

HR-MS (ESI) *m/z* calcd for $C_{29}H_{27}N_7O_7$ $[M+Na]^+$: 608.1864, found: 608.1859.

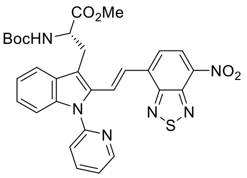

methyl (*S*,*E*)-2-((*tert*-butoxycarbonyl)amino)-3-(2-(2-(7-nitrobenzo[*c*][1,2,5]thiadiazol-4-yl)vinyl)-1-(pyridin-2-yl)-1*H*-indol-3-yl)propanoate (8)

The general procedure **A** was followed using methyl N_α-(*tert*-butoxycarbonyl)-1-(pyridin-2-yl)-*L*-tryptophanate (**1a**) (39.5 mg, 0.10 mmol), NBD-alkyne **S-3** (30.8 mg, 0.15 mmol), $MnBr(CO)_5$ (5.5 mg, 20 mol %), BPh_3 (9.7 mg, 40 mol %) and KOAc (3.9 mg, 40 mol %) in DCE (1.0 mL). Purification by column chromatography on silica gel (*n*-hexane/DCM/EtOAc = 1/1/1) yielded **8** (58.0 mg, 96%) as a purple solid.

M.p.: 95–97 °C.

^{1}H NMR (300 MHz, $CDCl_3$): δ 8.75 (dd, *J* = 5.6, 1.8 Hz, 1H), 8.54 (d, *J* = 8.1 Hz, 1H), 8.39 (d, *J* = 16.6 Hz, 1H), 7.93 (td, *J* = 7.7, 2.0 Hz, 1H), 7.71 (d, *J* = 8.1 Hz, 1H), 7.63 (d, *J* = 7.7 Hz, 1H), 7.48 – 7.36 (m, 3H), 7.29 – 7.14 (m, 2H), 7.00 (d, *J* = 16.6 Hz, 1H), 5.30 (d, *J* = 8.3 Hz, 1H), 4.76 (q, *J* = 7.1 Hz, 1H), 3.65 – 3.52 (m, 2H), 3.52 (s, 3H), 1.35 (s, 9H).

^{13}C NMR (75 MHz, $CDCl_3$): δ 172.6 (C_q), 155.1 (C_q), 154.0 (C_q), 151.8 (C_q), 150.0 (CH), 147.3 (C_q), 139.2 (C_q), 138.8 (CH), 137.9 (C_q), 137.2 (C_q), 134.2 (C_q), 128.9 (C_q), 128.5 (CH), 126.7 (CH), 125.3 (CH), 125.0 (CH), 124.0 (CH), 122.9 (CH), 122.5 (CH), 121.7 (CH), 119.6 (CH), 117.4 (C_q), 111.1 (CH), 80.1 (C_q), 54.5 (CH), 52.6 (CH_2), 29.1 (CH_3), 28.4 (CH_3, overlapped, 3C).

IR (ATR): 3382, 2977, 1742, 1705, 1512, 1313, 1165, 735 cm^{-1}.

MS (ESI) *m/z* (relative intensity): 601 $[M+H]^+$ (10), 623 $[M+Na]^+$ (100).

HR-MS (ESI) *m/z* calcd for $C_{30}H_{28}N_6O_6S$ $[M+H]^+$: 601.1864, found: 601.1865.

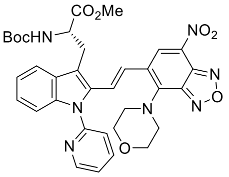

methyl (*S*,*E*)-2-((*tert*-butoxycarbonyl)amino)-3-(2-(2-(4-morpholino-7-nitrobenzo[*c*][1,2,5]oxadiazol-5-yl)vinyl)-1-(pyridin-2-yl)-1*H*-indol-3-yl)propanoate (9)

The general procedure **A** was followed using methyl N_α-(*tert*-butoxycarbonyl)-1-(pyridin-2-yl)-*L*-tryptophanate (**1a**) (39.5 mg, 0.10 mmol), NBD-alkyne **S-6** (41.1 mg, 0.15 mmol), $MnBr(CO)_5$ (5.5 mg, 20 mol %), BPh_3 (9.7 mg, 40 mol %) and KOAc (3.9 mg, 40 mol %) in DCE (1.0 mL). Purification by column chromatography on silica gel (*n*-hexane/DCM/EtOAc = 1/1/1) yielded **9** (63.2 mg, 94%) as a red solid.

M.p.: 204–206 °C.

^{1}H NMR (300 MHz, $CDCl_3$): δ 8.79 – 8.73 (m, 1H), 8.72 (s, 1H), 7.91 (td, *J* = 7.7, 2.0 Hz, 1H), 7.60 (d, *J* = 7.4 Hz, 1H), 7.45 – 7.31 (m, 3H), 7.30 – 7.14 (m, 3H), 6.45 (d, *J* = 16.5 Hz, 1H), 5.19 (d, *J* = 8.2 Hz, 1H), 4.68 (d, *J* = 7.6 Hz, 1H), 3.83 – 3.76 (m, 4H), 3.74 – 3.71 (m, 4H), 3.59 (s, 3H), 3.50 (brd, *J* = 6.3 Hz, 2H), 1.34 (s, 9H).

^{13}C NMR (75 MHz, $CDCl_3$): δ 172.6 (C_q), 155.1 (C_q), 152.1 (C_q), 150.0 (CH), 147.7 (C_q), 143.8 (C_q), 142.5 (C_q), 138.9 (CH), 138.7 (C_q), 134.5 (CH), 133.8 (C_q), 129.0 (C_q), 127.5 (C_q), 126.4 (CH), 124.7 (CH), 122.8 (CH), 122.3 (CH), 121.7 (CH), 121.4 (C_q), 120.1 (CH), 119.4 (CH), 115.1 (C_q), 110.9 (CH), 80.1 (C_q), 67.3 (CH_2, overlapped, 2C), 54.5 (CH), 53.0 (CH_2, overlapped, 2C), 52.6 (CH_3), 28.6 (CH_2), 28.3 (CH_3, overlapped, 3C).

IR (ATR): 3377, 2976, 1742, 1708, 1510, 1437, 1345, 1269, 1166, 733 cm^{-1}.

MS (ESI) *m/z* (relative intensity): 670 $[M+H]^+$ (5), 692 $[M+Na]^+$ (100).

HR-MS (ESI) *m/z* calcd for $C_{34}H_{35}N_7O_8$ $[M+Na]^+$: 692.2439, found: 692.2431.

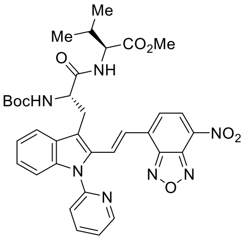

methyl ((*S*)-2-((*tert*-butoxycarbonyl)amino)-3-(2-((*E*)-2-(7-nitrobenzo[*c*][1,2,5]oxadiazol-4-yl)vinyl)-1-(pyridin-2-yl)-1*H*-indol-3-yl)propanoyl)-*L*-valinate (10)

The general procedure **A** was followed using methyl N_α-(*tert*-butoxycarbonyl)-1-(pyridin-2-yl)-*L*-tryptophyl-*L*-valinate (**1f**) (49.5 mg, 0.10 mmol), NBD-alkyne **2** (28.4 mg, 0.15 mmol), $MnBr(CO)_5$ (5.5 mg, 20 mol %), BPh_3 (9.7 mg, 40 mol %) and KOAc (3.9 mg, 40 mol %) in DCE (1.0 mL). Purification by column chromatography on silica gel (*n*-hexane/EtOAc = 3/2) yielded **10** (63.6 mg, 93%) as a purple solid.

M.p.: 130–132 °C.

^{1}H NMR (300 MHz, $CDCl_3$): δ 8.76 (dd, *J* = 5.2, 1.8 Hz, 1H), 8.39 (d, *J* = 16.7 Hz, 1H), 8.34 (d, *J* = 7.9 Hz, 1H), 8.00 (td, *J* = 7.7, 2.0 Hz, 1H), 7.70 (d, *J* = 7.9 Hz, 1H), 7.57 – 7.42 (m, 4H), 7.32 (d, *J* = 8.2 Hz, 1H), 7.20 (t, *J* = 7.9 Hz, 1H), 7.12 (t, *J* = 7.4 Hz, 1H), 5.99 (d, *J* = 8.1 Hz, 1H), 5.65 (d, *J* = 7.7 Hz, 1H), 4.45 (ddd, *J* = 9.9, 7.7, 5.3 Hz, 1H), 4.19 (dd, *J* = 8.2, 4.6 Hz, 1H), 3.58 (dd, *J* = 14.1, 5.2 Hz, 1H), 3.41 (dd, *J* = 14.1, 10.2 Hz, 1H), 3.32 (s, 3H), 2.03 – 1.86 (m, 1H), 1.48 (s, 9H), 0.78 (d, *J* = 6.8 Hz, 3H), 0.73 (d, *J* = 6.9 Hz, 3H).

^{13}C NMR (75 MHz, $CDCl_3$): δ 170.8 (C_q), 155.3 (C_q), 151.0 (C_q), 150.1 (CH), 148.4 (C_q), 143.4 (C_q), 138.8 (CH), 138.5 (C_q), 136.5 (C_q), 133.6 (C_q), 133.5 (C_q), 131.7 (CH), 130.2 (CH), 129.1 (C_q), 127.2 (CH), 125.4 (CH), 124.5 (CH), 123.1 (CH), 122.5 (CH), 121.8 (CH), 119.5 (CH), 116.5 (C_q), 111.0 (CH), 80.3 (C_q), 57.3 (CH), 55.6 (CH), 52.1 (CH_3), 32.0 (CH), 30.1 (CH_2), 28.5 (CH_3, overlapped, 3C), 18.7 (CH_3), 17.9 (CH_3). (One aromatic C_q is missing due to overlap.)

IR (ATR): 3370, 1970, 1743, 1667, 1513, 1436, 1317, 1155, 745 cm^{-1}.

MS (ESI) *m/z* (relative intensity): 694 $[M+H]^+$ (20), 706 $[M+Na]^+$ (100).

HR-MS (ESI) *m/z* calcd for $C_{35}H_{37}N_7O_8$ $[M+Na]^+$: 706.2596, found: 706.2590.

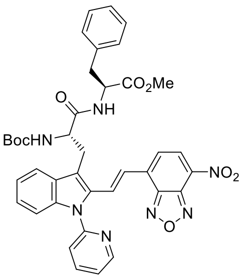

methyl ((*S*)-2-((*tert*-butoxycarbonyl)amino)-3-(2-((*E*)-2-(7-nitrobenzo[*c*][1,2,5]oxadiazol-4-yl)vinyl)-1-(pyridin-2-yl)-1*H*-indol-3-yl)propanoyl)-*L*-phenylalaninate (11)

The general procedure **A** was followed using methyl N_α-(*tert*-butoxycarbonyl)-1-(pyridin-2-yl)-*L*-tryptophyl-*L*-phenylalaninate (**1g**) (54.3 mg, 0.10 mmol), NBD-alkyne **2** (28.4 mg, 0.15 mmol), $MnBr(CO)_5$ (5.5 mg, 20 mol %), BPh_3 (9.7 mg, 40 mol %) and KOAc (3.9 mg, 40 mol %) in DCE (1.0 mL). Purification by column chromatography on silica gel (*n*-hexane/EtOAc = 3/2) yielded **11** (53.4 mg, 73%) as a purple solid.

M.p.: 204–206 °C.

^{1}H NMR (300 MHz, $CDCl_3$): δ 8.75 (dd, *J* = 5.0, 1.9 Hz, 1H), 8.41 (d, *J* = 15.8 Hz, 1H), 8.39 (d, *J* = 7.9 Hz, 1H), 7.97 (td, *J* = 7.7, 2.0 Hz, 1H), 7.73 (d, *J* = 7.9 Hz, 1H), 7.59 (d, *J* = 7.8 Hz, 1H), 7.53 – 7.36 (m, 3H), 7.33 (d, *J* = 8.1 Hz, 1H), 7.22 (t, *J* = 7.5 Hz, 1H), 7.18 – 7.12 (m, 4H), 6.89 (dd, *J* = 6.8, 2.7 Hz, 2H), 5.89 (d, *J* = 7.0 Hz, 1H), 5.62 (d, *J* = 7.6 Hz, 1H), 4.47 – 4.39 (m, 2H), 3.61 (dd, *J* = 14.1, 4.9 Hz, 1H), 3.42 (dd, *J* = 14.1, 10.1 Hz, 1H), 3.35 (s, 3H), 2.99 (dd, *J* = 13.8, 5.9 Hz, 1H), 2.90 (dd, *J* = 13.8, 5.2 Hz, 1H), 1.49 (s, 9H).

^{13}C NMR (75 MHz, $CDCl_3$): δ 170.4 (C_q), 155.3 (C_q), 151.0 (C_q), 150.1 (CH), 148.5 (C_q), 143.5 (C_q), 138.8 (CH), 136.5 (C_q), 135.6 (C_q), 133.8 (C_q), 133.6 (C_q), 131.7 (CH), 130.1 (CH), 129.3 (CH, overlapped, 2C), 129.1 (C_q), 128.5 (CH, overlapped, 2C), 127.2 (CH), 125.4 (CH), 124.6 (CH), 123.1 (CH), 122.4 (CH), 121.9 (CH), 119.6 (CH), 116.7 (CH), 111.0 (CH), 80.3 (C_q), 55.4 (CH), 53.7 (CH), 52.2 (CH_3), 38.2 (CH_2), 30.1 (CH_2), 28.5 (CH_3, overlapped, 3C). (Three aromatic C_q are missing due to overlap.)

IR (ATR): 3368, 2977, 1743, 1671, 1514, 1437, 1317, 1166, 733 cm^{-1}.

MS (ESI) *m/z* (relative intensity): 732 $[M+H]^+$ (20), 754 $[M+Na]^+$ (100).

HR-MS (ESI) *m/z* calcd for $C_{39}H_{37}N_7O_8$ $[M+H]^+$: 732.2776, found: 732.2778.

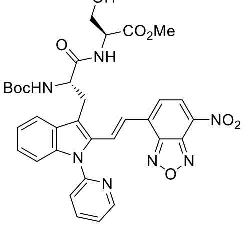

methyl ((*S*)-2-((*tert*-butoxycarbonyl)amino)-3-(2-((*E*)-2-(7-nitrobenzo[*c*][1,2,5]oxadiazol-4-yl)vinyl)-1-(pyridin-2-yl)-1*H*-indol-3-yl)propanoyl)-*L*-serinate (12)

The general procedure **A** was followed using methyl *N*$_\alpha$-(*tert*-butoxycarbonyl)-1-(pyridin-2-yl)-*L*-tryptophyl-*L*-serinate (**1h**) (48.3 mg, 0.10 mmol), NBD-alkyne **2** (28.4 mg, 0.15 mmol), $MnBr(CO)_5$ (5.5 mg, 20 mol %), BPh_3 (9.7 mg, 40 mol %) and KOAc (3.9 mg, 40 mol %) in DCE (1.0 mL). Purification by column chromatography on silica gel (*n*-hexane/EtOAc = 1/2) yielded **12** (39.6 mg, 59%) as a purple solid.

M.p.: 189–191 °C.

^{1}H NMR (300 MHz, $CDCl_3$): δ 8.69 (dd, *J* = 4.9, 1.3 Hz, 1H), 8.43 (d, *J* = 7.9 Hz, 1H), 8.35 (d, *J* = 16.6 Hz, 1H), 8.06 (td, *J* = 7.7, 1.9 Hz, 1H), 7.70 (d, *J* = 8.1 Hz, 1H), 7.67 – 7.58 (m, 2H), 7.50 (ddd, *J* = 7.5, 4.9, 1.0 Hz, 1H), 7.35 – 7.17 (m, 3H), 7.10 (d, *J* = 16.6 Hz, 1H), 6.57 (s, 1H), 5.50 (d, *J* = 7.7 Hz, 1H), 4.59 (td, *J* = 8.0, 4.9 Hz, 1H), 4.32 (s, 1H), 3.88 – 3.66 (m, 3H), 3.53 (s, 3H), 3.46 (dd, *J* = 14.5, 8.5 Hz, 1H), 3.38 (s, 1H), 1.46 (s, 9H).

^{13}C NMR (75 MHz, $CDCl_3$): δ 171.2 (C_q), 169.7 (C_q), 155.4 (C_q), 150.8 (C_q), 150.0 (CH), 148.5 (C_q), 143.2 (C_q), 139.3 (CH), 138.8 (C_q), 135.9 (C_q), 133.7 (C_q), 133.5 (C_q), 131.6 (CH), 129.2 (CH), 128.9 (C_q), 126.2 (CH), 125.6 (CH), 123.7 (CH), 123.4 (CH), 122.5 (CH), 121.9 (CH), 119.5 (CH), 117.6 (C_q), 110.7 (CH), 80.5 (C_q), 62.6 (CH_2), 55.5 (CH), 55.0 (CH), 52.5 (CH_3), 29.0 (CH_2), 28.4 (CH_3, overlapped, 3C).

IR (ATR): 3435, 3270, 2978, 1738, 1697, 1665, 1515, 1435, 1311, 1152, 908, 728 cm^{-1}.

MS (ESI) *m/z* (relative intensity): 672 $[M+H]^+$ (15), 694 $[M+Na]^+$ (100).

HR-MS (ESI) *m/z* calcd for $C_{33}H_{33}N_7O_9$ $[M+H]^+$: 672.2413, found: 672.2420.

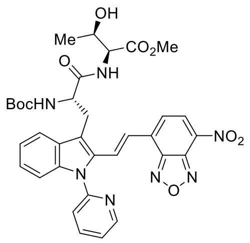

methyl ((*S*)-2-((*tert*-butoxycarbonyl)amino)-3-(2-((*E*)-2-(7-nitrobenzo[*c*][1,2,5]oxadiazol-4-yl)vinyl)-1-(pyridin-2-yl)-1*H*-indol-3-yl)propanoyl)-*L*-threoninate (13)

The general procedure **A** was followed using methyl *N*$_\alpha$-(*tert*-butoxycarbonyl)-1-(pyridin-2-yl)-*L*-tryptophyl-*L*-threoninate (**1i**) (49.7 mg, 0.10 mmol), NBD-alkyne **2** (28.4 mg, 0.15 mmol), $MnBr(CO)_5$ (5.5 mg, 20 mol %), BPh_3 (9.7 mg, 40 mol %) and KOAc (3.9 mg, 40 mol %) in DCE (1.0 mL). Purification by column chromatography on silica gel (*n*-hexane/EtOAc = 1/2) yielded **13** (41.2 mg, 60%) as a purple solid.

M.p.: 135–137 °C.

^{1}H NMR (300 MHz, $CDCl_3$): δ 8.71 (d, *J* = 4.9 Hz, 1H), 8.35 (d, *J* = 7.9 Hz, 1H), 8.35 (d, *J* = 16.5 Hz, 1H), 8.00 (t, *J* = 7.7 Hz, 1H), 7.66 – 7.55 (m, 3H), 7.52 – 7.42 (m, 1H), 7.34 – 7.18 (m, 3H), 7.14 (t, *J* = 7.4 Hz, 1H), 6.45 (d, 1H), 5.63 (d, *J* = 7.6 Hz, 1H), 4.54 (q, *J* = 7.6 Hz, 1H), 4.38 – 4.25 (m, 1H), 4.03 (s, 1H), 3.59 (dd, *J* = 14.3, 5.4 Hz, 1H), 3.46 (dd, *J* = 14.3, 9.5 Hz, 1H), 3.39 (s, 3H), 2.76 (s, 1H), 1.45 (s, 9H), 1.07 (d, *J* = 6.5 Hz, 3H).

^{13}C NMR (75 MHz, $CDCl_3$): δ 171.6 (C_q), 170.1 (C_q), 155.5 (C_q), 151.1(C_q), 150.1 (CH), 148.5 (C_q), 143.4 (C_q), 139.1 (CH), 138.8 (C_q), 136.3 (C_q), 133.74 (C_q), 133.66 (C_q), 131.7 (CH), 129.8 (CH), 129.0 (C_q), 126.8 (CH), 125.6 (CH), 124.3 (CH), 123.3 (CH), 122.6 (CH), 121.9 (CH), 119.8 (CH), 117.3 (C_q), 110.9 (CH), 80.4 (C_q), 68.5 (CH), 57.8 (CH), 55.5 (CH), 52.5 (CH_3), 29.6 (CH_2), 28.5 (CH_3, overlapped, 3C), 20.0 (CH_3).

IR (ATR): 3332, 2978, 1742, 1663, 1511, 1435, 1308, 1153, 1086, 996, 908, 730 cm^{-1}.

MS (ESI) *m/z* (relative intensity): 708 $[M+Na]^+$ (100).

HR-MS (ESI) *m/z* calcd for $C_{34}H_{35}N_7O_9$ $[M+Na]^+$: 708.2388, found: 708.2375.

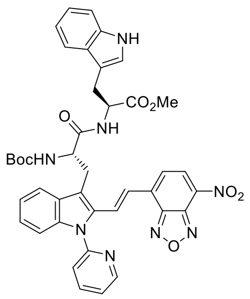

methyl ((*S*)-2-((*tert*-butoxycarbonyl)amino)-3-(2-((*E*)-2-(7-nitrobenzo[*c*][1,2,5]oxadiazol-4-yl)vinyl)-1-(pyridin-2-yl)-1*H*-indol-3-yl)propanoyl)-*L*-tryptophanate (14)

The general procedure **C** was followed using methyl N_{α}-(*tert*-butoxycarbonyl)-1-(pyridin-2-yl)-*L*-tryptophyl-*L*-tryptophanate (**1j**) (29.1 mg, 0.050 mmol), NBD-alkyne **2** (18.9 mg, 0.10 mmol), $MnBr(CO)_5$ (5.5 mg, 40 mol %), BPh_3 (9.7 mg, 80 mol %) and KOAc (3.9 mg, 80 mol %) in DCE (1.0 mL). Purification by column chromatography on silica gel (DCM/EtOAc = 2/1) yielded **14** (23.0 mg, 60%) as a purple solid.

M.p.: 140–142 °C.

^{1}H NMR (300 MHz, $CDCl_3$): δ 8.83 – 8.74 (m, 1H), 8.56 (s, 1H), 8.30 (d, *J* = 16.1 Hz, 1H), 8.30 (d, *J* = 8.0 Hz, 1H), 7.96 (td, *J* = 7.7, 1.9 Hz, 1H), 7.66 (d, *J* = 7.7 Hz, 1H), 7.58 – 7.46 (m, 2H), 7.43 (d, *J* = 8.0 Hz, 1H), 7.32 (t, *J* = 7.0 Hz, 2H), 7.28 – 7.04 (m, 4H), 7.00 (t, *J* = 7.3 Hz, 1H), 6.93 (t *J* = 7.5 Hz, 1H), 6.60 (d, *J* = 2.4 Hz, 1H), 6.03 (d, *J* = 7.1 Hz, 1H), 5.50 (d, *J* = 7.8 Hz, 1H), 4.54 – 4.46 (m, 2H), 3.63 (d, *J* = 14.2 Hz, 1H), 3.51 – 3.26 (m, 4H), 3.16 (dd, *J* = 14.8, 5.4 Hz, 1H), 3.05 (dd, *J* = 14.8, 5.5 Hz, 1H), 1.46 (s, 9H).

^{13}C NMR (75 MHz, $CDCl_3$): δ 171.1 (C_q), 170.5 (C_q), 155.3 (C_q), 151.1 (C_q), 150.0 (CH), 148.5 (C_q), 143.3 (C_q), 139.1 (CH), 138.8 (C_q), 136.2 (C_q), 136.0 (C_q), 133.7 (C_q), 133.7 (C_q), 131.6 (CH), 129.4 (CH), 129.1 (C_q), 127.4 (C_q), 126.5 (CH), 125.6 (CH), 124.3 (CH), 123.4 (CH), 123.0 (CH), 122.7 (CH), 122.0 (CH), 122.0 (CH), 120.0 (CH), 119.5 (CH), 118.4 (CH), 117.0 (C_q), 111.3 (CH), 110.9 (CH), 109.5 (C_q), 80.3 (C_q), 55.2 (CH), 53.3 (CH), 52.3 (CH_3), 29.4 (CH_2), 28.5 (CH_3, overlapped, 3C), 27.7 (CH_2).

IR (ATR): 3405, 2977, 1741, 1703, 1671, 1516, 1438, 1319, 741 cm^{-1}.

MS (ESI) *m/z* (relative intensity): 771 $[M+H]^+$ (20), 793 $[M+Na]^+$ (100).

HR-MS (ESI) *m/z* calcd for $C_{41}H_{38}N_8O_8$ $[M+H]^+$: 771.2885, found: 771.2892.

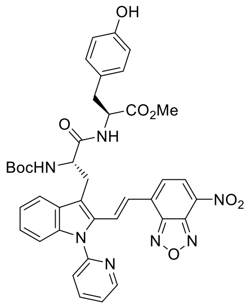

methyl ((*S*)-2-((*tert*-butoxycarbonyl)amino)-3-(2-((*E*)-2-(7-nitrobenzo[*c*][1,2,5]oxadiazol-4-yl)vinyl)-1-(pyridin-2-yl)-1*H*-indol-3-yl)propanoyl)-*L*-tyrosinate (15)

The general procedure **C** was followed using methyl N_{α}-(*tert*-butoxycarbonyl)-1-(pyridin-2-yl)-*L*-tryptophyl-*L*-tyrosinate (**1k**) (27.9 mg, 0.050 mmol), NBD-alkyne **2** (18.9 mg, 0.10 mmol), $MnBr(CO)_5$ (5.5 mg, 40 mol %), BPh_3 (9.7 mg, 80 mol %) and KOAc (3.9 mg, 80 mol %) in DCE (1.0 mL). Purification by column chromatography on silica gel (*n*-hexane/EtOAc = 3/2) yielded **15** (28.7 mg, 77%) as a purple solid.

M.p.: 142–144 °C.

^{1}H NMR (300 MHz, $CDCl_3$): δ 8.71 (dd, *J* = 5.1, 1.8 Hz, 1H), 8.28 (d, *J* = 16.5 Hz, 1H), 8.28 (d, *J* = 8.0 Hz, 1H), 7.98 (td, *J* = 7.7, 1.9 Hz, 1H), 7.60 – 7.55 (m, 2H), 7.51 – 7.45 (m, 2H), 7.29 – 7.10 (m, 4H), 6.78 (s, 1H), 6.55 (d, *J* = 8.0 Hz, 2H), 6.37 (d, *J* = 8.0 Hz, 2H), 6.19 (d, *J* = 7.3 Hz, 1H), 5.58 (d, *J* = 7.9 Hz, 1H), 4.51 – 4.40 (m, 2H), 3.56 (brd, J = 13.2 Hz, 1H), 3.37 (dd, *J* = 14.2, 9.9 Hz, 1H), 3.33 (s, 3H), 2.84 (dd, *J* = 14.1, 5.6 Hz, 1H), 2.71 (dd, *J* = 14.0, 5.3 Hz, 1H), 1.45 (s, 9H).

^{13}C NMR (75 MHz, $CDCl_3$): δ 170.6 (C_q), 155.4 (C_q), 155.2 (C_q), 151.0 (C_q), 149.9 (CH), 148.5 (C_q), 143.3 (C_q), 139.2 (CH), 138.7 (C_q), 136.3 (C_q), 133.7 (C_q), 133.6 (C_q), 131.7 (CH), 130.2 (CH, overlapped, 2C), 129.8 (CH), 129.1 (C_q), 126.8 (CH), 125.4 (CH), 124.5

(CH), 123.3 (CH), 122.6 (CH), 121.9 (CH), 119.7 (CH), 117.0 (C_q), 115.5 (CH, overlapped, 2C), 110.8 (CH), 80.4 (C_q), 55.2 (CH), 53.6 (CH), 52.3 (CH_3), 37.1 (CH_2), 29.7 (CH_2), 28.5 (CH_3, overlapped, 3C). (Two aromatic C_q are missing due to overlap.)
IR (ATR): 3373, 2931, 1742, 1668, 1514, 1437, 1316, 1157, 733 cm^{-1}.
MS (ESI) *m/z* (relative intensity): 770 $[M+Na]^+$ (100).
HR-MS (ESI) *m/z* calcd for $C_{39}H_{37}N_7O_9$ $[M+Na]^+$: 770.2545, found: 770.2536.

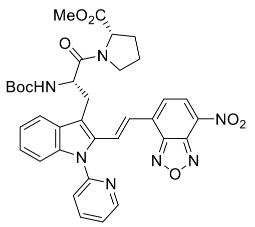

methyl ((*S*)-2-((*tert*-butoxycarbonyl)amino)-3-(2-((*E*)-2-(7-nitrobenzo[*c*][1,2,5]oxadiazol-4-yl)vinyl)-1-(pyridin-2-yl)-1*H*-indol-3-yl)propanoyl)-*L*-prolinate (16)

The general procedure **A** was followed using methyl N_α-(*tert*-butoxycarbonyl)-1-(pyridin-2-yl)-*L*-tryptophyl-*L*-prolinate (**1l**) (49.3 mg, 0.10 mmol), NBD-alkyne **2** (28.4 mg, 0.15 mmol), $MnBr(CO)_5$ (5.5 mg, 20 mol %), BPh_3 (9.7 mg, 40 mol %) and KOAc (3.9 mg, 40 mol %) in DCE (1.0 mL). Purification by column chromatography on silica gel (*n*-hexane/EtOAc = 1/1) yielded **16** (38.9 mg, 57%, 1:1 mixture of rotamers) as a purple solid.
M.p.: 181–183 °C.
^{1}H NMR (300 MHz, $CDCl_3$, 1:1 mixture of rotamers): δ 8.74 (dd, *J* = 5.0, 2.7 Hz, 1H), 8.48 – 8.31 (m, 2H), 8.00 (t, *J* = 7.4 Hz, 0.5H), 7.90 (dd, *J* = 8.0, 5.7 Hz, 1H), 7.81 (d, *J* = 7.6 Hz, 0.5H), 7.69 – 7.58 (m, 1H), 7.51 – 7.36 (m, 3H), 7.35 – 7.13 (m, 2.5H), 6.81 (d, *J* = 16.4 Hz, 0.5H), 5.77 (d, *J* = 8.1 Hz, 0.5H), 5.47 (d, *J* = 9.0 Hz, 0.5H), 4.99 (q, *J* = 7.9 Hz, 0.5H), 4.80 – 4.67 (m, 0.5H), 4.52 (dd, *J* = 8.7, 3.6 Hz, 0.5H), 3.74 – 3.30 (m, 7H), 3.07 (dt, *J* = 11.8, 7.7 Hz, 0.5H), 2.15 (q, *J* = 8.2 Hz, 0.5H), 2.00 – 1.82 (m, 1.5H), 1.66 (dq, *J* = 11.0, 3.4 Hz, 0.5H), 1.53 (s, 4.5H), 1.27 (s, 4.5H), 1.20 – 0.91 (m, 1.5H).
^{13}C NMR (75 MHz, $CDCl_3$, 1:1 mixture of rotamers): δ 172.3 (C_q), 172.0 (C_q), 170.7 (C_q), 155.2 (C_q), 155.1 (C_q), 151.9 (C_q), 150.8 (C_q), 150.3 (CH), 149.9 (CH), 148.5 (C_q), 143.5 (C_q), 143.5 (C_q), 139.5 (C_q), 138.9 (CH), 138.8 (CH), 137.9 (C_q), 136.6 (C_q), 136.3 (C_q), 134.1 (C_q), 133.9 (C_q), 133.9 (C_q), 133.7 (C_q), 131.8 (CH), 131.6 (CH), 130.0 (CH), 129.2 (C_q), 128.8 (C_q), 127.8 (CH), 126.3 (CH), 125.8 (CH), 125.7 (CH), 125.3 (CH), 124.3 (CH), 123.1 (CH), 122.9 (CH), 122.5 (CH), 122.0 (CH), 121.9 (CH), 121.8 (CH), 120.3 (CH), 120.1 (CH), 118.9 (C_q), 116.0 (C_q), 111.2 (CH), 111.1 (CH), 79.9 (C_q), 79.8 (C_q), 59.0 (CH), 59.0 (CH), 52.8 (CH_3), 52.6 (CH_3), 52.4 (CH), 52.2 (CH), 47.4 (CH_2), 46.4 (CH_2), 31.5 (CH_2), 30.0 (CH_2), 29.4 (CH_2), 29.2 (CH_2), 28.6 (CH_3), 28.3 (CH_3), 24.8 (CH_2), 22.2(CH_2).
IR (ATR): 3057, 2977, 1743, 1703, 1642, 1588, 1515, 1468, 1435, 1367, 1316, 1227, 1165, 1087, 997, 734 cm^{-1}.
MS (ESI) *m/z* (relative intensity): 704 $[M+Na]^+$ (100).
HR-MS (ESI) *m/z* calcd for $C_{35}H_{35}N_7O_8$ $[M+Na]^+$: 704.2439, found: 704.2446.

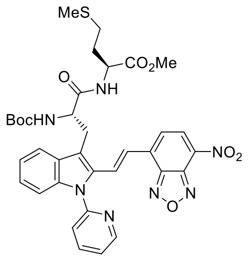

methyl ((*S*)-2-((*tert*-butoxycarbonyl)amino)-3-(2-((*E*)-2-(7-nitrobenzo[*c*][1,2,5]oxadiazol-4-yl)vinyl)-1-(pyridin-2-yl)-1*H*-indol-3-yl)propanoyl)-*L*-methioninate (17)

The general procedure **C** was followed using methyl N_α-(*tert*-butoxycarbonyl)-1-(pyridin-2-yl)-*L*-tryptophyl-*L*-methioninate (**1m**) (26.3 mg, 0.050 mmol), NBD-alkyne **2** (18.9 mg, 0.10 mmol), $MnBr(CO)_5$ (5.5 mg, 40 mol %), BPh_3 (9.7 mg, 80 mol %) and KOAc (3.9 mg, 80 mol %) in DCE (1.0 mL). Purification by column chromatography on silica gel (*n*-hexane/EtOAc = 3/2) yielded **17** (30.2 mg, 84%) as a purple solid.
M.p.: 181–183 °C.

^{1}H NMR (300 MHz, $CDCl_3$): δ 8.76 (dd, *J* = 4.9, 1.9 Hz, 1H), 8.41 (d, *J* = 15.7 Hz, 1H), 8.37 (d, *J* = 7.9 Hz, 1H), 8.00 (td, *J* = 7.7, 1.9 Hz, 1H), 7.71 (d, *J* = 7.9 Hz, 1H), 7.56 (d, *J* = 7.9 Hz, 2H), 7.49 (dd, *J* = 7.4, 4.9 Hz, 1H), 7.43 (d, *J* = 15.7 Hz, 1H), 7.34 (d, *J* = 8.2 Hz, 1H), 7.22 (dd, *J* = 8.2, 7.1 Hz, 1H), 7.18 (dd, *J* = 7.9, 7.1 Hz, 1H), 6.08 (d, *J* = 7.1 Hz, 1H), 5.63 (d, *J* = 7.6 Hz, 1H), 4.47 (ddd, *J* = 9.7, 7.6, 5.2 Hz, 1H), 4.31 (d, *J* = 6.4 Hz, 1H), 3.61 (dd, *J* = 14.2, 5.2 Hz, 1H), 3.41 (dd, *J* = 14.2, 9.7 Hz, 1H), 3.39 (s, 3H), 2.43 – 2.18 (m, 2H), 2.03 – 1.92 (m, 1H), 1.97 (s, 3H), 1.85 – 1.73 (m, 1H), 1.47 (s, 9H).

^{13}C NMR (75 MHz, $CDCl_3$): δ 170.9 (C_q), 170.7 (C_q), 155.3 (C_q), 151.0 (C_q), 150.1 (CH), 148.5 (C_q), 143.4 (C_q), 138.9 (CH), 138.6 (C_q), 136.5 (C_q), 133.8 (C_q), 133.6 (C_q), 131.7 (CH), 130.1 (CH), 129.1 (C_q), 127.2 (CH), 125.4 (CH), 124.5 (CH), 123.1 (CH), 122.4 (CH), 121.9 (CH), 119.5 (CH), 116.6 (C_q), 111.0 (CH), 80.4 (C_q), 55.4 (CH), 52.5 (CH_3), 51.8 (CH), 32.1 (CH_2), 30.0 (CH_2), 29.6 (CH_2), 28.5 (CH_3, overlapped, 3C), 15.5 (CH_3).

IR (ATR): 3345, 2977, 1743, 1668, 1513, 1437, 1315, 1158, 733 cm^{-1}.

MS (ESI) *m/z* (relative intensity): 738 $[M+Na]^+$ (100).

HR-MS (ESI) *m/z* calcd for $C_{35}H_{37}N_7O_8S$ $[M+H]^+$: 716.2497, found: 716.2483.

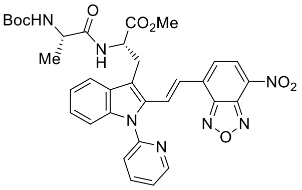

methyl (*S*)-2-((*S*)-2-((*tert*-butoxycarbonyl)amino)propanamido)-3-(2-((*E*)-2-(7-nitrobenzo[*c*][1,2,5]oxadiazol-4-yl)vinyl)-1-(pyridin-2-yl)-1*H*-indol-3-yl)propanoate (18)

The general procedure **A** was followed using methyl *N*$_\alpha$-((*tert*-butoxycarbonyl)-*L*-alanyl)-1-(pyridin-2-yl)-*L*-tryptophanate (**1n**) (46.7 mg, 0.10 mmol), NBD-alkyne **2** (28.4 mg, 0.15 mmol), $MnBr(CO)_5$ (5.5 mg, 20 mol %), BPh_3 (9.7 mg, 40 mol %) and KOAc (3.9 mg, 40 mol %) in DCE (1.0 mL). Purification by column chromatography on silica gel (*n*-hexane/EtOAc = 2/3) yielded **18** (49.8 mg, 76%) as a purple solid.

M.p.: 199–201 °C.

^{1}H NMR (300 MHz, $CDCl_3$): δ 8.78 – 8.69 (m, 1H), 8.38 (d, *J* = 7.8 Hz, 1H), 8.36 (d, *J* = 16.6 Hz, 1H), 7.97 (td, *J* = 7.7, 1.9 Hz, 1H), 7.54 (d, *J* = 7.8 Hz, 1H), 7.53 (d, *J* = 7.8, 1H), 7.49 – 7.40 (m, 2H), 7.36 (d, *J* = 8.1 Hz, 1H), 7.24 (t, *J* = 7.7 Hz, 1H), 7.18 (t, J = 7.4 Hz, 1H), 7.07 (d, *J* = 16.6 Hz, 1H), 6.98 (d, *J* = 7.3 Hz, 1H), 5.05 (s, 1H), 4.96 (td, *J* = 8.3, 5.5 Hz, 1H), 4.17 (s, 1H), 3.61 (dd, *J* = 14.4, 5.5 Hz, 1H), 3.50 (dd, *J* = 14.4, 8.3 Hz, 1H), 3.45 (s, 3H), 1.42 (s, 9H), 1.31 (d, *J* = 7.1 Hz, 3H).

^{13}C NMR (75 MHz, $CDCl_3$): δ 172.6 (C_q), 172.1 (C_q), 155.5 (C_q), 151.2 (C_q), 150.1 (CH), 148.5 (C_q), 143.4 (C_q), 138.9 (CH), 138.8 (C_q), 136.1 (C_q), 133.9 (C_q), 131.7 (CH), 129.7 (CH), 128.9 (C_q), 126.9 (CH), 125.7 (CH), 124.4 (CH), 123.1 (CH), 122.3 (CH), 121.9 (CH), 119.5 (CH), 117.2 (C_q), 111.2 (CH), 80.4 (C_q), 53.0 (CH), 52.7 (CH_3), 50.4 (CH), 29.2 (CH_2), 28.4 (CH_3, overlapped, 3C), 18.5 (CH_3). (One aromatic C_q is missing due to overlap.)

IR (ATR): 3319, 2979, 1740, 1666, 1513, 1436, 1310, 1156, 1087, 910, 730 cm^{-1}.

MS (ESI) *m/z* (relative intensity): 656 $[M+H]^+$ (20), 678 $[M+Na]^+$ (100).

HR-MS (ESI) *m/z* calcd for $C_{33}H_{33}N_7O_8$ $[M+Na]^+$: 656.2463, found: 656.2467.

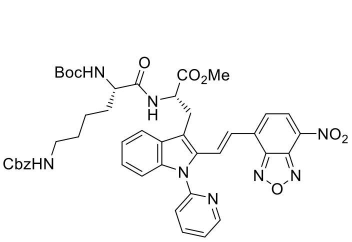

methyl (*S*)-2-((*S*)-6-(((benzyloxy)carbonyl)amino)-2-((*tert*-butoxycarbonyl)amino)hexanamido)-3-(2-((*E*)-2-(7-nitrobenzo[*c*][1,2,5]oxadiazol-4-yl)vinyl)-1-(pyridin-2-yl)-1*H*-indol-3-yl)propanoate (19)

The general procedure **C** was followed using methyl *N*$_\alpha$-(*N*$_6$-((benzyloxy)carbonyl)-*N*$_2$-(*tert*-butoxycarbonyl)-*L*-lysyl)-1-(pyridin-2-yl)-*L*-tryptophanate (**1o**) (32.9 mg, 0.050 mmol), NBD-alkyne **2** (18.9 mg, 0.10 mmol), $MnBr(CO)_5$ (5.5 mg, 40 mol %), BPh_3 (9.7 mg, 80

mol %) and KOAc (3.9 mg, 80 mol %) in DCE (1.0 mL). Purification by column chromatography on silica gel (DCM/EtOAc = 3/2) yielded **19** (27.9 mg, 66%) as a purple solid.

M.p.: 104–106 °C.

^{1}H NMR (300 MHz, $CDCl_3$): δ 8.67 (dd, *J* = 4.9, 1.9 Hz, 1H), 8.31 (d, *J* = 16.7 Hz, 1H), 8.30 (d, *J* = 7.9 Hz, 1H), 7.90 (td, *J* = 7.7, 2.0 Hz, 1H), 7.48 (d, *J* = 7.8 Hz, 1H), 7.43 – 7.36 (m, 3H), 7.32 – 7.10 (m, 8H), 6.98 (d, *J* = 16.7 Hz, 1H), 6.92 (s, 1H), 5.17 (s, 1H), 5.01 (s, 2H), 4.95 – 4.88 (m, 2H), 4.01 (s, 1H),3.53 (dd, *J* = 14.3, 5.9 Hz, 1H), 3.43 (dd, *J* = 14.3, 8.4 Hz, 1H), 3.39 (s, 3H), 3.08 (q, *J* = 6.6 Hz, 2H), 1.72 (s, 1H), 1.35 (m, 14H).

^{13}C NMR (75 MHz, $CDCl_3$): δ 172.2 (C_q), 172.0 (C_q), 156.8 (C_q), 155.8 (C_q), 151.2 (C_q), 150.1 (CH), 148.5 (C_q), 143.4 (C_q), 138.9 (CH), 138.8 (C_q), 136.7 (C_q), 136.0 (C_q), 133.9 (C_q), 133.8 (C_q), 131.7 (CH), 129.6 (CH), 128.8 (C_q), 128.6 (CH), 128.2 (CH), 127.0 (CH), 125.8 (CH), 124.5 (CH), 123.1 (CH), 122.3 (CH), 121.9 (CH), 119.5 (CH), 117.2 (C_q), 111.3 (CH), 80.3 (C_q), 66.8 (CH_2), 54.6 (CH), 53.0 (CH), 52.7 (CH_3), 40.3 (CH_2), 31.9 (CH_2), 29.5 (CH_2), 29.1 (CH_2), 28.4 (CH_3, overlapped, 3C), 22.4 (CH_2). (Three aromatic CH are missing due to overlap.)

IR (ATR): 3314, 2939, 1707, 1670, 1518, 1438, 1321, 1248, 1169, 744 cm^{-1}.

MS (ESI) *m/z* (relative intensity): 847 $[M+H]^+$ (35), 869 $[M+Na]^+$ (100).

HR-MS (ESI) *m/z* calcd for $C_{44}H_{46}N_8O_{10}$ $[M+H]^+$: 847.3410, found: 847.3408.

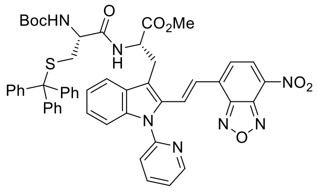

methyl (*S*)-2-((*R*)-2-((*tert*-butoxycarbonyl)amino)-3-(tritylthio)propanamido)-3-(2-((*E*)-2-(7-nitrobenzo[*c*][1,2,5]oxadiazol-4-yl)vinyl)-1-(pyridin-2-yl)-1*H*-indol-3-yl)propanoate (20)

The general procedure **A** was followed using methyl *N*$_\alpha$-(*N*-(*tert*-butoxycarbonyl)-*S*-trityl-*L*-cysteinyl)-1-(pyridin-2-yl)-*L*-tryptophanate (**1p**) (74.1 mg, 0.10 mmol), NBD-alkyne **2** (28.4 mg, 0.15 mmol), $MnBr(CO)_5$ (5.5 mg, 20 mol %), BPh_3 (9.7 mg, 40 mol %) and KOAc (3.9 mg, 40 mol %) in DCE (1.0 mL). Purification by column chromatography on silica gel (*n*-hexane/EtOAc = 2/3) yielded **20** (62.3 mg, 67%) as a purple solid.

M.p.: 112–114 °C.

^{1}H NMR (300 MHz, $CDCl_3$): δ 8.76 (dd, *J* = 5.4, 1.9 Hz, 1H), 8.41 (d, *J* = 8.9 Hz, 1H), 8.40 (d, *J* = 16.6 Hz, 1H), 7.97 (td, *J* = 7.7, 1.9 Hz, 1H), 7.64 (d, *J* = 7.9 Hz, 1H), 7.56 (d, *J* = 7.8 Hz, 1H), 7.50 – 7.44 (m, 2H), 7.44 – 7.33 (m, 8H), 7.31 – 7.12 (m, 11H), 6.91 (s, 1H), 4.93 – 4.86 (m, 2H), 3.91 (s, 1H), 3.61 (dd, *J* = 14.3, 5.2 Hz, 1H), 3.46 (dd, *J* = 14.3, 8.4 Hz, 1H), 3.41 (s, 3H), 2.80 (s, 1H), 2.53 (dd, *J* = 12.8, 5.1 Hz, 1H), 1.41 (s, 9H).

^{13}C NMR (75 MHz, $CDCl_3$): δ 172.0 (C_q), 170.4 (C_q), 155.4 (C_q), 151.2 (C_q), 150.1 (CH), 148.5 (C_q), 144.4 (C_q), 143.4 (C_q), 138.9 (CH), 138.7 (C_q), 136.2 (C_q), 133.9 (C_q), 133.8 (C_q), 131.8 (CH), 129.8 (CH), 129.6 (CH), 128.9 (C_q), 128.2 (CH), 127.1 (CH), 127.0 (CH), 125.6 (CH), 124.5 (CH), 123.1 (CH), 122.3 (CH), 121.8 (CH), 119.5 (CH), 117.0 (C_q), 111.2 (CH), 80.6 (C_q), 67.3 (C_q), 53.7 (CH), 53.2 (CH), 52.7 (CH_3), 33.8 (CH_2), 29.4 (CH_2), 28.3 (CH_3, overlapped, 3C). (Twelve aromatic CH and two aromatic C_q are missing due to overlap.)

IR (ATR): 3402, 2978, 1739, 1707, 1673, 1514, 1437, 1314, 1156, 909, 730, 701 cm^{-1}.

MS (ESI) *m/z* (relative intensity): 952 $[M+Na]^+$ (100).

HR-MS (ESI) *m/z* calcd for $C_{52}H_{47}N_7O_8S$ $[M+Na]^+$: 952.3099, found: 952.3095.

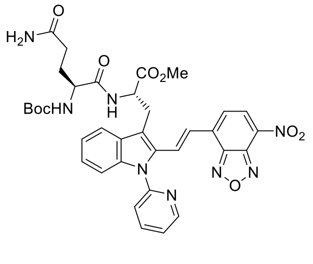

methyl (*S*)-2-((*S*)-5-amino-2-((*tert*-butoxycarbonyl)amino)-5-oxopentanamido)-3-(2-((*E*)-2-(7-nitrobenzo[*c*][1,2,5]oxadiazol-4-yl)vinyl)-1-(pyridin-2-yl)-1*H*-indol-3-yl)propanoate (21)

The general procedure **B** was followed using methyl N_α-((*tert*-butoxycarbonyl)-*L*-glutaminyl)-1-(pyridin-2-yl)-*L*-tryptophanate (**1q**) (26.2 mg, 0.050 mmol), NBD-alkyne **2** (28.4 mg, 0.15 mmol), $MnBr(CO)_5$ (13.7 mg, 100 mol %), BPh_3 (24.2 mg, 200 mol %) and KOAc (9.8 mg, 200 mol %) in DCE (1.0 mL). Purification by column chromatography on silica gel (DCM/MeOH/AcOH = 150/10/1) yielded **21** (26.7 mg, 75%) as a purple solid.

M.p.: 215–218 °C.

^{1}H NMR (400 MHz, Acetone-d_6): δ 8.76 (d, *J* = 5.0 Hz, 1H), 8.60 (d, *J* = 7.6 Hz, 1H), 8.42 (d, *J* = 16.6 Hz, 1H), 8.11 (t, *J* = 7.7 Hz, 1H), 7.98 (d, *J* = 8.3 Hz, 1H), 7.82 (d, *J* = 7.8 Hz, 1H), 7.75 (d, *J* = 7.9 Hz, 1H), 7.64 – 7.54 (m, 2H), 7.41 (d, *J* = 8.2 Hz, 1H), 7.27 (t, *J* = 7.6 Hz, 1H), 7.22 (t, *J* = 7.4 Hz, 1H), 7.13 (d, *J* = 16.6 Hz, 1H), 6.84 (s, 1H), 6.27 (s, 1H), 6.21 (s, 1H), 4.97 (d, *J* = 7.6 Hz, 1H), 4.15 (s, 1H), 3.64 (d, *J* = 6.8 Hz, 2H), 3.56 (s, 3H), 2.29 (t, *J* = 7.2 Hz, 2H), 2.20 – 2.10 (m, 1H), 1.91 – 1.81 (m, 1H), 1.35 (s, 9H).

^{13}C NMR (75 MHz, Acetic acid-d_4): δ 174.6 (C_q), 173.2 (C_q), 157.5 (C_q), 151.9 (C_q), 150.4 (CH), 149.7 (C_q), 144.6 (C_q), 141.5 (CH), 140.4 (C_q), 136.2 (C_q), 135.4 (C_q), 135.1 (C_q), 132.7 (CH), 129.7 (C_q), 129.2 (CH), 128.2 (CH), 126.6 (CH), 125.6 (CH), 124.9 (CH), 124.4 (CH), 122.9 (CH), 120.8 (CH), 118.8 (C_q), 111.8 (CH), 81.4 (C_q), 55.2 (CH), 54.2 (CH), 53.4 (CH_3), 32.4 (CH_2), 29.2 (CH_2), 28.8 (CH_2), 28.5 (CH_3, overlapped, 3C). (One aromatic C_q is missing due to overlap.)

IR (ATR): 3430, 3330, 3307, 1759, 1650, 1594, 1519, 1433, 1303, 1240, 1168, 1091, 997, 745 cm^{-1}.

MS (ESI) *m/z* (relative intensity): 735 $[M+Na]^+$ (100).

HR-MS (ESI) *m/z* calcd for $C_{35}H_{36}N_8O_9$ $[M+Na]^+$: 735.2497, found: 735.2506.

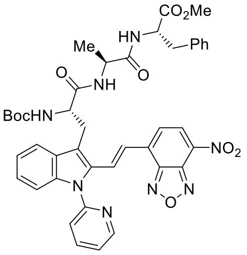

methyl ((*S*)-2-((*tert*-butoxycarbonyl)amino)-3-(2-((*E*)-2-(7-nitrobenzo[*c*][1,2,5]oxadiazol-4-yl)vinyl)-1-(pyridin-2-yl)-1*H*-indol-3-yl)propanoyl)-*L*-alanyl-*L*-phenylalaninate (22)

The general procedure **C** was followed using methyl N_α-(*tert*-butoxycarbonyl)-1-(pyridin-2-yl)-*L*-tryptophyl-*L*-alanyl-*L*-phenylalaninate (**1r**) (30.7 mg, 0.050 mmol), NBD-alkyne **2** (18.9 mg, 0.10 mmol), $MnBr(CO)_5$ (5.5 mg, 40 mol %), BPh_3 (9.7 mg, 80 mol %) and KOAc (3.9 mg, 80 mol %) in DCE (1.0 mL). Purification by column chromatography on silica gel (DCM/MeOH = 10/1) yielded **22** (26.9 mg, 67%) as a purple solid.

M.p.: 200–202 °C.

^{1}H NMR (300 MHz, $CDCl_3$): δ 8.75 (dd, *J* = 5.0, 1.9 Hz, 1H), 8.44 (d, *J* = 16.6 Hz, 1H), 8.35 (d, *J* = 7.8 Hz, 1H), 7.98 (td, *J* = 7.7, 2.0 Hz, 1H), 7.68 – 7.51 (m, 3H), 7.48 (dd, *J* = 7.5, 4.9 Hz, 1H), 7.34 (d, *J* = 8.0 Hz, 1H), 7.30 – 7.09 (m, 6H), 6.99 (d, *J* = 6.6 Hz, 2H), 6.51 (d, *J* = 6.7 Hz, 1H), 6.17 (s, 1H), 5.50 (d, *J* = 7.6 Hz, 1H), 4.59 – 4.52 (m, 2H), 4.19 (s, 1H), 3.65 (s, 3H), 3.57 – 3.47 (m, 2H), 3.05 – 2.88 (m, 2H), 1.38 (s, 9H), 1.21 (d, *J* = 7.0 Hz, 3H).

^{13}C NMR (75 MHz, $CDCl_3$): δ 171.6 (C_q), 170.9 (C_q), 170.8 (C_q), 155.4 (C_q), 151.2 (C_q), 150.1 (CH), 148.5 (C_q), 143.5 (C_q), 139.0 (CH), 136.3 (C_q), 135.7 (C_q), 133.8 (C_q), 133.6 (C_q), 131.7 (CH), 129.9 (CH), 129.3 (CH), 129.1 (C_q), 128.8 (CH), 127.8 (CH), 127.4 (CH), 127.0 (CH), 125.6 (CH), 124.5 (CH), 123.2 (CH), 122.6 (CH), 121.8 (CH), 119.8 (CH), 117.4 (C_q), 111.2 (CH), 80.5 (C_q), 55.3 (CH), 53.4 (CH), 52.5 (CH_3), 49.0 (CH), 37.9 (CH_2), 29.3 (CH_2), 28.4 (CH_3, overlapped, 3C), 18.60 (CH_3). (One aromatic CH and one aromatic C_q are missing due to overlap.)

IR (ATR): 3308, 2977, 1740, 1690, 1643, 1513, 1469, 1436, 1367, 1314, 1230, 1155, 1086, 996, 733, 700 cm^{-1}.

MS (ESI) *m/z* (relative intensity): 825 $[M+Na]^+$ (100).

HR-MS (ESI) *m/z* calcd for $C_{42}H_{42}N_8O_9$ $[M+Na]^+$: 825.2967, found: 825.2964.

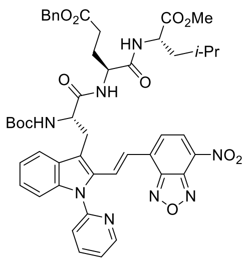

methyl (6*S*,9*S*,12*S*)-9-(3-(benzyloxy)-3-oxopropyl)-12-isobutyl-2,2-dimethyl-6-((2-((*E*)-2-(7-nitrobenzo[*c*][1,2,5]oxadiazol-4-yl)vinyl)-1-(pyridin-2-yl)-1*H*-indol-3-yl)methyl)-4,7,10-trioxo-3-oxa-5,8,11-triazatridecan-13-oate (23)

The general procedure **C** was followed using methyl (6*S*,9*S*,12*S*)-9-(3-(benzyloxy)-3-oxopropyl)-12-isobutyl-2,2-dimethyl-4,7,10-trioxo-6-((1-(pyridin-2-yl)-1*H*-indol-3-yl)methyl)-3-oxa-5,8,11-triazatridecan-13-oate (**1s**) (36.4 mg, 0.050 mmol), NBD-alkyne **2** (18.9 mg, 0.10 mmol), $MnBr(CO)_5$ (5.5 mg, 40 mol %), BPh_3 (9.7 mg, 80 mol %) and KOAc (3.9 mg, 80 mol %) in DCE (1.0 mL). Purification by column chromatography on silica gel (DCM/Et_2O = 3/1) yielded **23** (30.7 mg, 67%) as a purple solid.

M.p.: 214–216 °C.

^{1}H NMR (300 MHz, $CDCl_3$): δ 8.76 (dd, *J* = 4.9, 1.8 Hz, 1H), 8.44 (d, *J* = 16.8 Hz, 1H), 8.39 (dd, *J* = 7.6, 2.2 Hz, 1H), 7.98 (td, *J* = 7.7, 1.9 Hz, 1H), 7.63 (d, *J* = 8.1 Hz, 2H), 7.58 (d, *J* = 7.9 Hz, 1H), 7.47 (dd, *J* = 7.5, 4.9 Hz, 1H), 7.38 – 7.29 (m, 7H), 7.22 (t, *J* = 7.5 Hz, 1H), 7.15 (t, *J* = 7.3 Hz, 1H), 6.57 (d, *J* = 6.5 Hz, 1H), 6.45 (d, *J* = 7.3 Hz, 1H), 5.46 (d, *J* = 7.2 Hz, 1H), 5.11 (d, *J* = 12.8 Hz, 1H), 5.07 (d, *J* = 12.8 Hz, 1H), 4.51 (q, *J* = 7.3 Hz, 1H), 4.20 (s, 2H), 3.66 (s, 3H), 3.66 – 3.59 (m, 1H), 3.52 – 3.44 (m, 1H), 2.52 (dt, *J* = 16.5, 7.0 Hz, 1H), 2.40 (dt, *J* = 16.5, 6.7 Hz 1H), 2.03 (dt, *J* = 13.4, 7.0 Hz, 1H), 1.98 – 1.77 (m, 1H), 1.57 – 1.40 (m, 3H), 1.40 (s, 9H), 0.88 (d, *J* = 5.8 Hz, 3H), 0.83 (d, *J* = 5.8 Hz, 3H).

^{13}C NMR (75 MHz, $CDCl_3$): δ 173.7 (C_q), 172.9 (C_q), 170.9 (C_q), 170.1 (C_q), 155.4 (C_q), 151.0 (C_q), 150.0 (CH), 148.4 (C_q), 143.4 (C_q), 138.8 (CH), 138.7 (C_q), 136.3 (C_q), 135.7 (C_q), 133.7 (C_q), 133.6 (C_q), 131.6 (CH), 129.9 (CH), 128.9 (C_q), 128.7 (CH), 128.4 (CH), 128.2 (CH), 127.0 (CH), 125.5 (CH), 124.5 (CH), 123.2 (CH), 122.7 (CH), 121.7 (CH), 119.6 (CH), 116.9 (C_q), 111.1 (CH), 80.3 (C_q), 66.7 (CH_2), 55.3 (CH), 52.3 (CH_3), 52.1 (CH), 51.3 (CH), 40.8 (CH_2), 30.1 (CH_2), 29.3 (CH_2), 28.6 (CH_2), 28.4 (CH_3, overlapped, 3C), 24.8 (CH), 22.6 (CH_3), 22.2 (CH_3). (Two aromatic CH are missing due to overlap.)

IR (ATR): 3362, 2959, 1738, 1656, 1517, 1438, 1322, 1166, 744 cm^{-1}.

MS (ESI) *m/z* (relative intensity): 939 $[M+Na]^+$ (100).

HR-MS (ESI) *m/z* calcd for $C_{48}H_{52}N_8O_{11}$ $[M+Na]^+$: 939.3648, found: 939.3679.

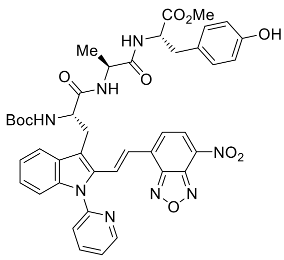

methyl ((*S*)-2-((*tert*-butoxycarbonyl)amino)-3-(2-((*E*)-2-(7-nitrobenzo[*c*][1,2,5]oxadiazol-4-yl)vinyl)-1-(pyridin-2-yl)-1*H*-indol-3-yl)propanoyl)-*L*-alanyl-*L*-tyrosinate (24)

The general procedure **C** was followed using methyl N_α-(*tert*-butoxycarbonyl)-1-(pyridin-2-yl)-*L*-tryptophyl-*L*-alanyl-*L*-tyrosinate (**1t**) (31.5 mg, 0.050 mmol), NBD-alkyne **2** (18.9 mg, 0.10 mmol), $MnBr(CO)_5$ (5.5 mg, 40 mol %), BPh_3 (9.7 mg, 80 mol %) and KOAc (3.9 mg, 80 mol %) in DCE (1.0 mL). Purification by column chromatography on silica gel (DCM/EtOAc = 2/1) yielded **24** (22.9 mg, 56%) as a purple solid.

M.p.: 154–157 °C.

^{1}H NMR (400 MHz, $CDCl_3$): δ 8.71 (dd, *J* = 5.0, 1.9 Hz, 1H), 8.34 (d, *J* = 7.8 Hz, 1H), 8.33 (d, *J* = 16.6 Hz, 1H), 8.05 (td, *J* = 7.8, 1.9 Hz, 1H), 7.65 (d, *J* = 7.8 Hz, 1H), 7.59 (d, *J* = 7.9 Hz, 1H), 7.52 (dd, *J* = 7.4, 5.1 Hz, 1H), 7.33 (d, *J* = 8.2 Hz, 1H), 7.29 – 7.22 (m, 3H), 7.20 (t, *J* = 7.4 Hz, 1H), 6.92 (d, *J* = 7.6 Hz, 1H), 6.69 – 6.65 (m, 3H), 6.41 (d, *J* = 8.4 Hz, 2H), 5.22 (d, *J* = 9.1 Hz, 1H), 4.73 (q, *J* = 5.7 Hz, 1H), 4.63 (s, 1H), 4.50 – 4.42 (m, 1H), 3.83 (dd, *J* = 14.8, 4.8 Hz, 1H) 3.82 (s, 3H), 3.34 (dd, *J* = 14.8, 8.4 Hz, 1H), 3.02 (dd, *J* = 14.1, 5.2 Hz, 1H), 2.89 (dd, *J* = 14.1, 5.0 Hz, 1H), 1.34 (d, *J* = 7.0 Hz, 3H), 1.20 (s, 9H). (OH proton is missing.)

^{13}C NMR (101 MHz, $CDCl_3$): δ 171.9 (C_q), 171.4 (C_q), 170.8 (C_q), 155.8 (C_q), 155.7 (C_q), 150.6 (C_q), 149.9 (CH), 148.4 (C_q), 143.4 (C_q), 139.5 (CH), 138.3 (C_q), 135.9 (C_q), 133.6 (C_q), 133.4 (C_q), 132.2 (CH), 130.3 (CH, overlapped, 2C), 129.4 (CH), 129.1 (C_q), 128.1 (CH), 126.5 (C_q), 125.5 (CH), 125.3 (CH), 123.4 (CH), 122.5 (CH), 121.9 (CH), 119.7 (CH), 117.9 (C_q), 115.5 (CH, overlapped, 2C), 110.8 (CH), 81.1 (C_q), 56.2 (CH), 53.6 (CH), 52.6 (CH_3), 48.8 (CH), 36.6 (CH_2), 29.2 (CH_2), 28.1 (CH_3, overlapped, 3C), 17.0 (CH_3).
IR (ATR): 3320, 2978, 1653, 1515, 1439, 1318, 1231, 1162, 746 cm^{-1}.
MS (ESI) *m/z* (relative intensity): 841 $[M+Na]^+$ (100).
HR-MS (ESI) *m/z* calcd for $C_{42}H_{42}N_8O_{10}$ $[M+Na]^+$: 841.2916, found: 841.2928.

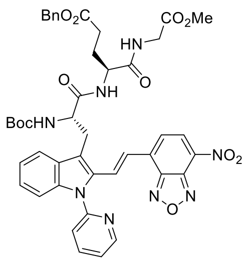

methyl (6*S*,9*S*)-9-(3-(benzyloxy)-3-oxopropyl)-2,2-dimethyl-6-((2-((*E*)-2-(7-nitrobenzo[*c*][1,2,5]oxadiazol-4-yl)vinyl)-1-(pyridin-2-yl)-1*H*-indol-3-yl)methyl)-4,7,10-trioxo-3-oxa-5,8,11-triazatridecan-13-oate (25)

The general procedure **C** was followed using methyl (6*S*,9*S*)-9-(3-(benzyloxy)-3-oxopropyl)-2,2-dimethyl-4,7,10-trioxo-6-((1-(pyridin-2-yl)-1*H*-indol-3-yl)methyl)-3-oxa-5,8,11-triazatridecan-13-oate (**1u**) (33.6 mg, 0.050 mmol), NBD-alkyne **2** (18.9 mg, 0.10 mol), $MnBr(CO)_5$ (5.5 mg, 40 mol %), BPh_3 (9.7 mg, 80 mol %) and KOAc (3.9 mg, 80 mol %) in DCE (1.0 mL). Purification by column chromatography on silica gel (DCM/EtOAc = 1/1) yielded **25** (27.1 mg, 63%) as a purple solid.
M.p.: 198–200 °C.
^{1}H NMR (300 MHz, $CDCl_3$): δ 8.74 (d, *J* = 4.8 Hz, 1H), 8.41 (d, *J* = 15.6 Hz, 1H), 8.37 (d, *J* = 7.5 Hz, 1H), 7.98 (t, *J* = 7.7 Hz, 1H), 7.67 – 7.46 (m, 4H), 7.39 – 7.28 (m, 7H), 7.23 – 7.13 (m, 2H), 6.74 (d, *J* = 7.4 Hz, 1H), 6.24 (s, 1H), 5.57 (d, *J* = 7.1 Hz, 1H), 5.04 (s, 2H), 4.52 (q, *J* = 7.3 Hz, 1H), 4.36 – 4.27 (m, 1H), 3.68 – 3.56 (m, 6H), 3.48 (dd, *J* = 13.2, 8.9 Hz, 1H), 2.48 (dt, *J* = 15.1, 7.3 Hz, 1H), 2.35 (dt, *J* = 15.1, 6.8 Hz, 1H), 2.08 – 1.78 (m, 2H), 1.43 (s, 9H).
^{13}C NMR (75 MHz, $CDCl_3$): δ 173.6 (C_q), 171.3 (C_q), 170.3 (C_q), 169.9 (C_q), 155.7 (C_q), 151.0 (C_q), 150.1 (CH), 148.5 (C_q), 143.5 (C_q), 138.9 (CH), 138.6 (C_q), 136.3 (C_q), 135.7 (C_q), 133.9 (C_q), 133.7 (C_q), 131.7 (CH), 129.9 (CH), 129.0 (C_q), 128.7 (CH), 128.4 (CH), 128.2 (CH), 127.7 (CH), 127.3 (CH), 125.4 (CH), 124.8 (CH), 123.2 (CH), 122.6 (CH), 122.0 (CH), 119.6 (CH), 116.6 (C_q), 111.1 (CH), 80.6 (C_q), 66.7 (CH_2), 55.6 (CH), 52.4 (CH_3), 52.4 (CH), 41.2 (CH_2), 30.2 (CH_2), 29.5 (CH_2), 28.5 (CH_3, overlapped, 3C), 28.0 (CH_2). (One aromatic CH is missing due to overlap.)
IR (ATR): 3299, 2977, 1738, 1868, 1642, 1617, 1438, 1316, 1231, 1170, 745 cm^{-1}.
MS (ESI) *m/z* (relative intensity): 883 $[M+Na]^+$ (100).
HR-MS (ESI) *m/z* calcd for $C_{44}H_{44}N_8O_{11}$ $[M+Na]^+$: 883.3022, found: 883.3044.

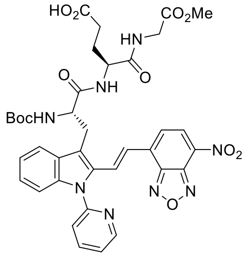

(*S*)-4-((*S*)-2-((*tert*-butoxycarbonyl)amino)-3-(2-((*E*)-2-(7-nitrobenzo[*c*][1,2,5]oxadiazol-4-yl)vinyl)-1-(pyridin-2-yl)-1*H*-indol-3-yl)propanamido)-5-((2-methoxy-2-oxoethyl)amino)-5-oxopentanoic acid (26)

The general procedure **B** was followed using (*S*)-4-((*S*)-2-((*tert*-butoxycarbonyl)amino)-3-(1-(pyridin-2-yl)-1*H*-indol-3-yl)propanamido)-5-((2-methoxy-2-oxoethyl)amino)-5-oxopentanoic acid (**1v**) (29.1 mg, 0.050 mmol), NBD-alkyne **2** (28.4 mg, 0.15 mmol), $MnBr(CO)_5$ (13.7 mg, 100 mol %), BPh_3 (24.2 mg, 200 mol %) and KOAc (9.8 mg, 200 mol %) in DCE (1.0 mL). Purification by column chromatography on silica gel (DCM/MeOH/AcOH = 150/10/1) yielded **26** (20.8 mg, 54%) as a purple solid.

M.p.: 182–185 °C.

^{1}H NMR (400 MHz, Acetone-*d*$_6$): δ 8.79 (dd, *J* = 4.9, 1.9 Hz, 1H), 8.61 (d, *J* = 7.8 Hz, 1H), 8.42 (d, *J* = 16.6 Hz, 1H), 8.14 (td, *J* = 7.7, 2.0 Hz, 1H), 7.82 (t, *J* = 7.2 Hz, 2H), 7.67 (d, *J* = 7.9 Hz, 1H), 7.62 (dd, *J* = 6.5, 4.8 Hz, 1H), 7.49 (d, *J* = 7.8 Hz, 1H), 7.41 – 7.37 (m, 2H), 7.32 – 7.24 (m, 2H), 7.20 (t, *J* = 6.9 Hz, 1H), 6.39 (d, *J* = 8.2 Hz, 1H), 4.67 (q, *J* = 7.3 Hz, 1H), 4.49 (q, *J* = 6.7 Hz, 1H), 3.85 (dd, *J* = 17.5, 5.6 Hz, 1H), 3.72 (dd, *J* = 17.5, 5.8 Hz, 1H), 3.68 – 3.57 (m, 2H), 3.62 (s, 3H), 2.37 (t, *J* = 7.8 Hz, 2H), 2.12 – 2.08 (m, 1H), 1.84 (dt, *J* = 14.8, 7.6 Hz, 1H), 1.36 (s, 9H). (CO_2H proton is missing.)

^{13}C NMR (101 MHz, Acetone-*d*$_6$): δ 173.5 (C$_q$), 171.2 (C$_q$), 170.8 (C$_q$), 169.8 (C$_q$), 155.4 (C$_q$), 151.3 (C$_q$), 149.8 (CH), 148.7 (C$_q$), 143.7 (C$_q$), 139.0 (CH), 138.9 (C$_q$), 135.6 (C$_q$), 134.0 (C$_q$), 133.8 (C$_q$), 132.2 (CH), 129.2 (CH), 129.0 (C$_q$), 127.4 (CH), 125.0 (CH), 124.2 (CH), 123.2 (CH), 122.7 (CH), 121.3 (CH), 120.0 (CH), 117.9 (C$_q$), 110.9 (CH), 78.9 (C$_q$), 55.6 (CH), 52.1 (CH), 51.3 (CH_3), 40.6 (CH_2), 27.9 (CH_2), 27.6 (CH_3, overlapped, 3C). (Two aliphatic CH_2 are missing due to overlap.)

IR (ATR): 3327, 3301, 1747, 1708, 1687, 1643, 1592, 1515, 1438, 1368, 1310, 1229, 1169, 1090, 996 cm^{-1}.

MS (ESI) *m/z* (relative intensity): 769 [M–H]$^+$ (100).

HR-MS (ESI) *m/z* calcd for $C_{37}H_{38}N_8O_{11}$ [M–H]$^+$: 769.2587, found: 769.2589.

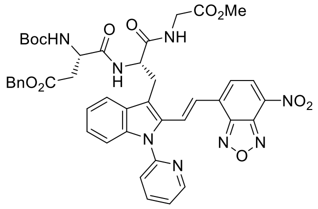

methyl (6*S*,9*S*)-6-(2-(benzyloxy)-2-oxoethyl)-2,2-dimethyl-9-((2-((*E*)-2-(7-nitrobenzo[*c*][1,2,5]oxadiazol-4-yl)vinyl)-1-(pyridin-2-yl)-1*H*-indol-3-yl)methyl)-4,7,10-trioxo-3-oxa-5,8,11-triazatridecan-13-oate (27)

The general procedure **C** was followed using methyl (6*S*,9*S*)-6-(2-(benzyloxy)-2-oxoethyl)-2,2-dimethyl-4,7,10-trioxo-9-((1-(pyridin-2-yl)-1*H*-indol-3-yl)methyl)-3-oxa-5,8,11-triazatridecan-13-oate (**1w**) (32.9 mg, 0.050 mmol), NBD-alkyne **2** (18.9 mg, 0.10 mol), MnBr(CO)$_5$ (5.5 mg, 40 mol %), BPh$_3$ (9.7 mg, 80 mol %) and KOAc (3.9 mg, 80 mol %) in DCE (1.0 mL). Purification by column chromatography on silica gel (DCM/EtOAc = 1/1) yielded **27** (27.9 mg, 66%) as a purple solid.

M.p.: 123–126 °C.

^{1}H NMR (400 MHz, $CDCl_3$): δ 8.74 (dd, *J* = 4.8, 1.9 Hz, 1H), 8.40 (d, *J* = 16.7 Hz, 1H), 8.36 (d, *J* = 7.9 Hz, 1H), 8.01 (td, *J* = 7.7, 1.9 Hz, 1H), 7.73 (d, *J* = 7.9 Hz, 1H), 7.64 (d, *J* = 7.8 Hz, 1H), 7.56 (dt, *J* = 7.9, 1.0 Hz, 1H), 7.51 – 7.42 (m, 3H), 7.39 (d, *J* = 8.3 Hz, 1H), 7.30 – 7.15 (m, 7H), 6.00 (s, 1H), 5.67 (d, *J* = 8.9 Hz, 1H), 5.10 (d, *J* = 12.3 Hz, 1H), 5.06 (d, *J* = 12.3 Hz, 1H), 4.74 (ddd, *J* = 9.8, 7.5, 4.9 Hz, 1H), 4.58 (s, 1H), 3.88 (dd, *J* = 18.2, 5.4 Hz, 1H), 3.58 (dd, *J* = 14.3, 5.0 Hz, 1H), 3.54 (s, 3H), 3.50 (dd, *J* = 18.2, 4.7 Hz, 1H), 3.46 (dd, *J* = 14.3, 9.9 Hz, 1H), 3.15 (dd, *J* = 17.1, 4.8 Hz, 1H), 2.79 (dd, *J* = 17.1, 5.4 Hz, 1H), 1.46 (s, 9H).

^{13}C NMR (101 MHz, $CDCl_3$): δ 171.9 (C$_q$), 170.8 (C$_q$), 170.4 (C$_q$), 169.2 (C$_q$), 155.6 (C$_q$), 151.0 (C$_q$), 150.2 (CH), 148.6 (C$_q$), 143.5 (C$_q$), 139.0 (CH), 138.4 (C$_q$), 136.3 (C$_q$), 135.3 (C$_q$), 133.9 (C$_q$), 133.9 (C$_q$), 131.9 (CH), 130.0 (CH), 129.2 (C$_q$), 128.7 (CH), 128.6 (CH), 128.3 (CH), 127.4 (CH), 125.6 (CH), 124.9 (CH), 123.2 (CH), 122.4 (CH), 122.0 (CH), 119.7 (CH), 116.4 (C$_q$), 111.2 (CH), 81.0 (C$_q$), 67.0 (CH_2), 54.5 (CH), 52.4 (CH_3), 51.1 (CH), 41.4 (CH_2), 36.4 (CH_2), 29.4 (CH_2), 28.4 (CH_3, overlapped, 3C). (Two aromatic CH are missing due to overlap.)

IR (ATR): 3323, 2932, 1738, 1654, 1517, 1438, 1320, 1231, 1157, 745 cm^{-1}.

MS (ESI) *m/z* (relative intensity): 869 [M+Na]$^+$ (100).

HR-MS (ESI) *m/z* calcd for $C_{43}H_{42}N_8O_{11}$ [M+Na]$^+$: 869.2865, found: 869.2869.

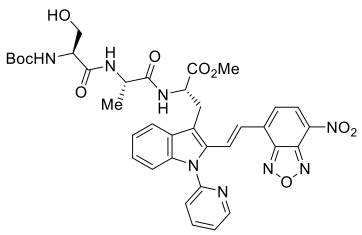

methyl (6*S*,9*S*,12*S*)-6-(hydroxymethyl)-2,2,9-trimethyl-12-((2-((*E*)-2-(7-nitrobenzo[*c*][1,2,5]oxadiazol-4-yl)vinyl)-1-(pyridin-2-yl)-1*H*-indol-3-yl)methyl)-4,7,10-trioxo-3-oxa-5,8,11-triazatridecan-13-oate (28)

The general procedure **A** was followed using methyl N_{α}-(*tert*-butoxycarbonyl)-*L*-seryl-*L*-alanyl-1-(pyridin-2-yl)-*L*-tryptophanate (**1x**) (55.4 mg, 0.10 mmol), NBD-alkyne **2** (28.4 mg, 0.15 mmol), $MnBr(CO)_5$ (5.5 mg, 20 mol %), BPh_3 (9.7 mg, 40 mol %) and KOAc (3.9 mg, 40 mol %) in DCE (1.0 mL). Purification by column chromatography on silica gel (DCM/*n*-hexane/MeOH = 90/20/1) yielded **28** (48.3 mg, 65%) as a purple solid.

M.p.: 179–182 °C.

^{1}H NMR (400 MHz, $CDCl_3$): δ 8.68 (dd, *J* = 5.1, 1.9 Hz, 1H), 8.34 (d, *J* = 7.8 Hz, 1H), 8.22 (d, *J* = 16.6 Hz, 1H), 8.00 (td, *J* = 7.8, 2.0 Hz, 1H), 7.54 (d, *J* = 8.1 Hz, 1H), 7.50 – 7.45 (m, 2H), 7.43 – 7.39 (m, 2H), 7.21 – 7.07 (m, 3H), 6.97 (d, *J* = 16.6 Hz, 1H), 6.85 (d, *J* = 7.9 Hz, 1H), 5.65 (d, *J* = 7.8 Hz, 1H), 4.98 (td, *J* = 8.6, 5.0 Hz, 1H), 4.71 (t, *J* = 7.6 Hz, 1H), 4.24 (s, 1H), 4.05 – 3.96 (m, 2H), 3.63 – 3.58 (m, 1H), 3.55 (dd, *J* = 14.1, 5.1 Hz, 1H), 3.44 (dd, *J* = 14.1, 8.8 Hz, 1H), 3.40 (s, 3H), 1.46 (s, 9H), 1.38 (d, *J* = 7.2 Hz, 3H).

^{13}C NMR (101 MHz, $CDCl_3$): δ 172.4 (C_q), 172.2 (C_q), 171.4 (C_q), 156.0 (C_q), 150.9 (C_q), 150.1 (CH), 148.3 (C_q), 143.4 (C_q), 139.2 (CH), 138.5 (C_q), 135.7 (C_q), 134.0 (C_q), 133.9 (C_q), 131.7 (CH), 129.5 (CH), 128.8 (C_q), 127.1 (CH), 125.7 (CH), 124.3 (CH), 123.4 (CH), 122.4 (CH), 121.8 (CH), 119.5 (CH), 116.6 (C_q), 111.0 (CH), 80.5 (C_q), 63.0 (CH_2), 55.1 (CH), 53.0 (CH), 52.8 (CH_3), 49.3 (CH), 29.2 (CH_2), 28.5 (CH_3, overlapped, 3C), 18.3 (CH_3).

IR (ATR): 3375, 2930, 1746, 1712, 1437, 1314, 1153, 745 cm^{-1}.

MS (ESI) *m/z* (relative intensity): 743 $[M+H]^+$ (15), 765 $[M+Na]^+$ (100).

HR-MS (ESI) *m/z* calcd for $C_{36}H_{38}N_8O_{10}$ $[M+Na]^+$: 765.2603, found: 765.2602.

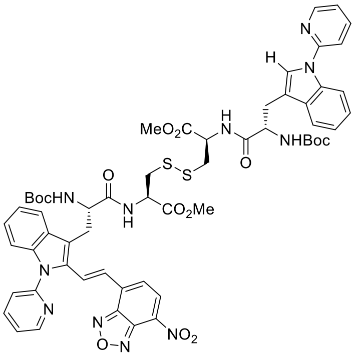

methyl *N*-(N_{α}-(*tert*-butoxycarbonyl)-1-(pyridin-2-yl)-*L*-tryptophyl)-*S*-(((*R*)-2-((*S*)-2-((*tert*-butoxycarbonyl)amino)-3-(2-((*E*)-2-(7-nitrobenzo[*c*][1,2,5]oxadiazol-4-yl)vinyl)-1-(pyridin-2-yl)-1*H*-indol-3-yl)propanamido)-3-methoxy-3-oxopropyl)thio)-*L*-cysteinate (29a)

The general procedure **C** was followed using methyl (6*S*,9*R*,14*R*)-14-((*S*)-2-((*tert*-butoxycarbonyl)amino)-3-(1-(pyridin-2-yl)-1*H*-indol-3-yl)propanamido)-9-(methoxycarbonyl)-2,2-dimethyl-4,7-dioxo-6-((1-(pyridin-2-yl)-1*H*-indol-3-yl)methyl)-3-oxa-11,12-dithia-5,8-diazapentadecan-15-oate (**1y**) (49.8 mg, 0.050 mmol), NBD-alkyne **2** (18.9 mg, 0.10 mol), $MnBr(CO)_5$ (5.5 mg, 40 mol %), BPh_3 (9.7 mg, 80 mol %) and KOAc (3.9 mg, 80 mol %) in DCE (1.0 mL). Purification by column chromatography on silica gel (DCM/EtOAc = 2/1) yielded mono-alkenylated product **29a** (18.9 mg, 31%) and di-alkenylated product **29b** (14.4 mg, 21%) as purple solids.

M.p.: 115–117 °C.

^{1}H NMR (300 MHz, $CDCl_3$): δ 8.72 (d, *J* = 3.5 Hz, 1H), 8.44 (dd, *J* = 4.9, 1.8 Hz, 1H), 8.35 (d, *J* = 16.5 Hz, 1H), 8.26 (d, *J* = 7.8 Hz, 1H), 8.12 (d, *J* = 8.3 Hz, 1H), 7.92 (td, *J* = 7.8, 2.0 Hz, 1H), 7.72 (td, *J* = 7.8, 1.9 Hz, 1H), 7.62 (d, *J* = 8.0 Hz, 1H), 7.56 – 7.52 (m, 3H), 7.45 – 7.41 (m, 2H), 7.35 (d, *J* = 8.1 Hz, 2H), 7.28 – 7.05 (m, 7H), 6.92 (d, *J* = 7.0 Hz, 1H), 5.86 (d, *J* = 8.2 Hz, 1H), 5.46 (s, 1H), 4.80 – 4.41 (m, 4H), 3.69 – 3.39 (m, 8H), 3.22 (d, *J* = 6.5 Hz, 2H), 3.06 (dd, *J* = 14.2, 5.2 Hz, 1H), 3.00 – 2.80 (m, 3H), 1.38 (s, 9H), 1.36 (s, 9H).

^{13}C NMR (101 MHz, $CDCl_3$): δ 172.0 (C_q), 171.5 (C_q), 170.4 (C_q), 169.7 (C_q), 155.8 (C_q), 155.5 (C_q), 152.3 (C_q), 151.2 (C_q), 150.0 (CH), 148.9 (CH), 148.5 (C_q), 143.4 (C_q), 138.8 (CH), 138.7 (C_q), 138.4 (CH), 136.2 (C_q), 135.3 (C_q), 133.7 (C_q), 133.6 (C_q), 131.7 (CH), 130.1 (C_q), 130.0 (CH), 129.2 (C_q), 126.9 (CH), 125.4 (CH), 124.7 (CH), 124.5 (CH), 123.6 (CH), 123.0 (CH), 122.4 (CH), 121.8 (CH), 121.3 (CH), 119.9 (CH), 119.8 (CH), 119.1 (CH), 117.4 (C_q), 114.2 (CH), 113.8 (C_q), 113.5 (CH), 111.1 (CH), 80.2 (C_q), 55.4 (CH), 54.8 (CH), 52.8 (CH_3, overlapped, 2C), 52.1 (CH), 51.9 (CH), 40.8 (CH_2), 40.5 (CH_2), 29.8 (CH_2), 28.5 (CH_3, overlapped, 3C), 28.4 (CH_3, overlapped, 3C), 28.1 (CH_2). (One aromatic C_q is missing due to overlap.)

IR (ATR): 3344, 2978, 1745, 1676, 1516, 1472, 1438, 1320, 1167, 746 cm^{-1}.

MS (ESI) *m/z* (relative intensity): 1184 [M+H]+ (10), 1206 [M+Na]+ (100).

HR-MS (ESI) *m/z* calcd for $C_{58}H_{61}N_{11}O_{13}S_2$ [M+Na]+: 1206.3784, found: 1206.3782.

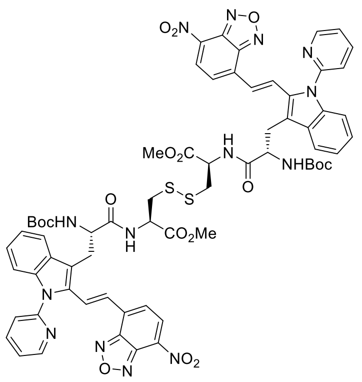

methyl (6*S*,9*R*,14*R*)-14-((*S*)-2-((*tert*-butoxycarbonyl)amino)-3-(2-((*E*)-2-(7-nitrobenzo[*c*][1,2,5]oxadiazol-4-yl)vinyl)-1-(pyridin-2-yl)-1*H*-indol-3-yl)propanamido)-9-(methoxycarbonyl)-2,2-dimethyl-6-((2-((*E*)-2-(7-nitrobenzo[*c*][1,2,5]oxadiazol-4-yl)vinyl)-1-(pyridin-2-yl)-1*H*-indol-3-yl)methyl)-4,7-dioxo-3-oxa-11,12-dithia-5,8-diazapentadecan-15-oate (29b)

M.p.: 121–123 °C.

^{1}H NMR (400 MHz, $CDCl_3$): δ 8.63 (d, *J* = 4.5 Hz, 2H), 8.33 (d, *J* = 16.5 Hz, 2H), 8.20 (d, *J* = 7.8 Hz, 2H), 7.91 (td, *J* = 7.7, 1.9 Hz, 2H), 7.49 (d, *J* = 7.9 Hz, 2H), 7.44 – 7.41 (m, 4H), 7.32 (d, *J* = 7.9 Hz, 2H), 7.22 (d, *J* = 8.3 Hz, 2H), 7.21 – 7.13 (m, 4H), 7.10 – 7.06 (m, 4H), 6.92 (d, *J* = 7.1 Hz, 2H), 5.71 (d, *J* = 8.4 Hz, 2H), 4.69 – 4.50 (m, 4H), 3.56 (s, 6H), 3.56 – 3.48 (m, 4H), 3.07 (dd, *J* = 14.2, 4.9 Hz, 2H), 3.02 – 2.91 (m, 2H), 1.34 (s, 18H).

^{13}C NMR (101 MHz, $CDCl_3$): δ 171.5 (C_q, overlapped, 2C), 169.9 (C_q, overlapped, 2C), 155.6 (C_q, overlapped, 2C), 151.1 (C_q, overlapped, 2C), 149.9 (CH, overlapped, 2C), 148.5 (C_q, overlapped, 2C), 143.4 (C_q, overlapped, 2C), 138.8 (CH, overlapped, 2C), 136.0 (C_q, overlapped, 2C), 133.6 (C_q, overlapped, 2C), 133.5 (C_q, overlapped, 2C), 131.8 (CH, overlapped, 2C), 129.5 (CH, overlapped, 2C), 129.0 (C_q, overlapped, 2C), 127.0 (CH, overlapped, 2C), 125.5 (CH, overlapped, 2C), 124.5 (CH, overlapped, 2C), 123.1 (CH, overlapped, 2C), 122.4 (CH, overlapped, 2C), 121.8 (CH, overlapped, 2C), 119.7 (CH, overlapped, 2C), 117.9 (C_q, overlapped, 2C), 111.1 (CH, overlapped, 2C), 80.4 (C_q, overlapped, 2C), 55.5 (CH, overlapped, 2C), 52.9 (CH_3, overlapped, 2C), 52.2 (CH, overlapped, 2C), 41.3 (CH_2, overlapped, 2C), 29.6 (CH_2, overlapped, 2C), 28.4 (CH_3, overlapped, 6C). (Two aromatic C_q are missing due to overlap.)

IR (ATR): 3388, 2929, 1745, 1680, 1515, 1469, 1437, 1368, 1320, 1166, 745 cm^{-1}.

MS (ESI) *m/z* (relative intensity): 1373 [M+H]+ (10), 1395 [M+Na]+ (100).

HR-MS (ESI) *m/z* calcd for $C_{66}H_{64}N_{14}O_{16}S_2$ [M+Na]+: 1395.3958, found: 1395.3952.

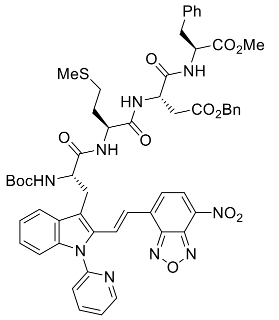

methyl (6*S*,9*S*,12*S*,15*S*)-15-benzyl-12-(2-(benzyloxy)-2-oxoethyl)-2,2-dimethyl-9-(2-(methylthio)ethyl)-6-((2-((*E*)-2-(7-nitrobenzo[*c*][1,2,5]oxadiazol-4-yl)vinyl)-1-(pyridin-2-yl)-1*H*-indol-3-yl)methyl)-4,7,10,13-tetraoxo-3-oxa-5,8,11,14-tetraazahexadecan-16-oate (30)

The general procedure **C** was followed using methyl (6*S*,9*S*,12*S*,15*S*)-15-benzyl-12-(2-(benzyloxy)-2-oxoethyl)-2,2-dimethyl-9-(2-(methylthio)ethyl)-4,7,10,13-tetraoxo-6-((1-(pyridin-2-yl)-1*H*-indol-3-yl)methyl)-3-oxa-5,8,11,14-tetraazahexadecan-16-oate (**1z**) (44.0 mg, 0.050 mmol), NBD-alkyne **2** (18.9 mg, 0.10 mol), $MnBr(CO)_5$ (5.5 mg, 40 mol %), BPh_3 (9.7 mg, 80 mol %) and KOAc (3.9 mg, 80

mol %) in DCE (1.0 mL). Purification by column chromatography on silica gel (DCM/EtOAc = 3/2) yielded **30** (23.0 mg, 43%) as a purple solid.

M.p.: 205–207 °C.

^{1}H NMR (400 MHz, $CDCl_3$): δ 8.74 (dd, *J* = 5.0, 1.9 Hz, 1H), 8.42 (d, *J* = 15.4 Hz, 1H), 8.39 (d, *J* = 5.9 Hz, 1H), 7.95 (td, *J* = 7.7, 2.0 Hz, 1H), 7.66 – 7.62 (m, 2H), 7.52 (d, *J* = 8.0 Hz, 1H), 7.45 (dd, *J* = 7.5, 4.9 Hz, 1H), 7.38 – 7.27 (m, 7H), 7.24 – 7.12 (m, 5H), 7.09 (d, *J* = 8.7 Hz, 2H), 7.00 (d, *J* = 7.9 Hz, 1H), 6.96 (d, *J* = 7.9 Hz, 1H), 6.67 (d, *J* = 6.8 Hz, 1H), 5.46 (d, *J* = 6.4 Hz, 1H), 5.11 (d, *J* = 12.2 Hz, 1H), 5.05 (d, *J* = 12.2 Hz, 1H), 4.72 (q, *J* = 7.1 Hz, 1H), 4.58 (q, *J* = 6.5 Hz, 1H), 4.48 (q, *J* = 6.9 Hz, 1H), 4.25 (d, *J* = 6.6 Hz, 1H), 3.68 (s, 3H), 3.62 (dd, *J* = 14.6, 6.0 Hz, 1H), 3.51 (dd, *J* = 14.6, 8.2 Hz, 1H), 3.08 (dd, *J* = 13.9, 5.7 Hz, 1H), 3.00 (dd, *J* = 13.9, 6.9 Hz, 1H), 2.74 (dd, *J* = 17.2, 5.1 Hz, 1H), 2.61 (dd, *J* = 17.2, 6.7 Hz, 1H), 2.36 (dt, *J* = 13.6, 7.0 Hz, 1H), 2.27 (dt, *J* = 13.6, 6.8 Hz, 1H), 1.96 (s, 3H), 1.91 – 1.73 (m, 2H), 1.40 (s, 9H).

^{13}C NMR (101 MHz, $CDCl_3$): δ 171.6 (C_q), 171.6 (C_q), 171.4 (C_q), 170.0 (C_q), 169.7 (C_q), 155.8 (C_q), 151.0 (C_q), 150.1 (CH), 148.5 (C_q), 143.4 (C_q), 139.0 (CH), 138.8 (C_q), 136.1 (C_q), 136.0 (C_q), 135.4 (C_q), 133.9 (C_q), 133.7 (C_q), 131.7 (CH), 129.7 (CH), 129.4 (CH), 129.3 (CH), 129.0 (C_q), 128.7 (CH), 128.7 (CH), 128.5 (CH), 128.4 (CH), 127.2 (CH), 125.6 (CH), 124.6 (CH), 123.3 (CH), 122.6 (CH), 121.9 (CH), 119.6 (CH), 116.8 (C_q), 111.2 (CH), 80.7 (C_q), 67.0 (CH_2), 55.8 (CH), 53.7 (CH), 52.9 (CH), 52.5 (CH_3), 49.4 (CH), 37.8 (CH_2), 35.7 (CH_2), 30.8 (CH_2), 29.9 (CH_2), 28.9 (CH_2), 28.4 (CH_3, overlapped, 3C), 15.2 (CH_3). (Four aromatic CH are missing due to overlap.)

IR (ATR): 3306, 3067, 2924, 1739, 1651, 1518, 1439, 1321, 1168, 748, 700 cm^{-1}.

MS (ESI) *m/z* (relative intensity): 1090 $[M+Na]^+$ (100).

HR-MS (ESI) *m/z* calcd for $C_{55}H_{57}N_9O_{12}S$ $[M+Na]^+$: 1090.3740, found: 1090.3756.

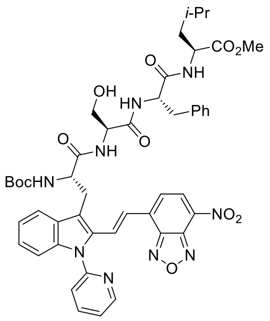

methyl ((*S*)-2-((*tert*-butoxycarbonyl)amino)-3-(2-((*E*)-2-(7-nitrobenzo[*c*][1,2,5]oxadiazol-4-yl)vinyl)-1-(pyridin-2-yl)-1*H*-indol-3-yl)propanoyl)-*L*-seryl-*L*-phenylalanyl-*L*-leucinate (31)

The general procedure **C** was followed using methyl N_α-(*tert*-butoxycarbonyl)-1-(pyridin-2-yl)-*L*-tryptophyl-*L*-seryl-*L*-phenylalanyl-*L*-leucinate (**1aa**) (37.1 mg, 0.050 mmol), NBD-alkyne **2** (18.9 mg, 0.10 mol), $MnBr(CO)_5$ (5.5 mg, 40 mol %), BPh_3 (9.7 mg, 80 mol %) and KOAc (3.9 mg, 80 mol %) in DCE (1.0 mL). Purification by column chromatography on silica gel (DCM/MeOH = 9/1) yielded **31** (21.9 mg, 47%) as a purple solid.

M.p.: 213–215 °C.

^{1}H NMR (600 MHz, $CDCl_3$): δ 8.73 (dd, *J* = 4.9, 1.1 Hz, 1H), 8.43 (d, *J* = 7.8 Hz, 1H), 8.39 (d, *J* = 16.6 Hz, 1H), 8.01 (td, *J* = 7.7, 2.0 Hz, 1H), 7.65 (d, *J* = 7.9 Hz, 1H), 7.58 (d, *J* = 7.9 Hz, 1H), 7.56 (d, *J* = 7.9 Hz, 1H), 7.50 (ddd, *J* = 7.6, 4.9, 1.0 Hz, 1H), 7.36 – 7.28 (m, 1H), 7.28 – 7.10 (m, 8H), 6.66 – 6.46 (m, 3H), 5.49 (d, *J* = 6.2 Hz, 1H), 4.54 – 4.49 (m, 3H), 4.10 (s, 1H), 3.76 – 3.70 (m, 1H), 3.67 (s, 3H), 3.66 – 3.58 (m, 1H), 3.53 – 3.39 (m, 2H), 3.17 (s, 1H), 3.07 (dd, *J* = 14.1, 6.2 Hz, 1H), 2.99 (dd, *J* = 14.1, 7.3 Hz, 1H), 1.53 – 1.34 (m, 12H), 0.84 (d, *J* = 6.2 Hz, 3H), 0.83 (d, *J* = 6.0 Hz, 3H).

^{13}C NMR (126 MHz, $CDCl_3$): δ 173.4 (C_q), 171.7 (C_q), 170.5 (C_q), 169.3 (C_q), 155.8 (C_q), 151.0 (C_q), 150.2 (CH), 148.6 (C_q), 143.5 (C_q), 139.2 (CH), 138.9 (C_q), 136.5 (C_q), 136.1 (C_q), 134.0 (C_q), 133.7 (C_q), 131.8 (CH), 129.5 (CH), 129.3 (CH), 129.0 (C_q), 128.9 (CH), 127.3 (CH), 127.0 (CH), 125.8 (CH), 124.5 (CH), 123.5 (CH), 122.7 (CH), 122.0 (CH), 119.4 (CH), 116.9 (C_q), 111.2 (CH), 80.8 (C_q), 62.4 (CH_2), 55.8 (CH), 55.0 (CH), 54.7 (CH), 52.5 (CH_3), 50.9 (CH), 41.4 (CH_2), 37.4 (CH_2), 29.0 (CH_2), 28.4 (CH_3, overlapped, 3C), 24.7 (CH), 22.9 (CH_3), 22.0 (CH_3). (One aromatic C_q is missing due to overlap.)

IR (ATR): 3309, 2958, 1739, 1691, 1644, 1516, 1438, 1325, 1172, 735 cm^{-1}.

MS (ESI) *m/z* (relative intensity): 954 $[M+Na]^+$ (100).

HR-MS (ESI) *m/z* calcd for $C_{48}H_{53}N_9O_{11}$ $[M+Na]^+$: 954.3757, found: 954.3769.

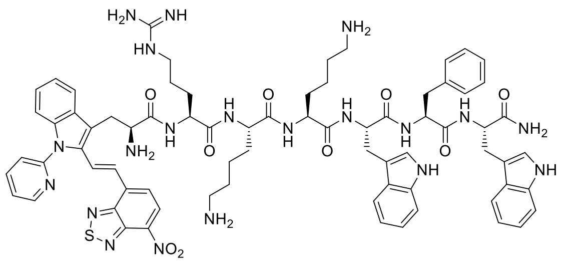

H-8-Arg-Lys-Lys-Trp-Phe-Trp-NH_2 (32)

The synthesis was performed on 70 mg of Rink Amide resin (0.18 mmol g^{-1}). Fmoc-Arg(Pbf)-OH, Fmoc-Lys(Boc)-OH, Fmoc-Trp(Boc)-OH and Fmoc-Phe-OH were used as building blocks. Automated microwave-assisted SPPS was carried out in a Liberty Blue microwave peptide synthesizer (CEM). DIC and OxymaPure reagents were used for amide couplings and 20% (v/v) piperidine/DMF was employed for the removal of Fmoc protecting groups. Prior to SPPS, amino acid ester **8** (8.0 mg, 0.013 mmol) was hydrolyzed by dissolving it in 1.0 mL MeOH followed by the addition of 250 µL 0.1 M $NaOH_{aq}$ at 0 °C and stirring for 7 h at room temperature. After solvent removal, the mono-protected amino acid **8’** was used without further purification. The amino acid **8’** (1.1 equiv) was then manually incorporated in the peptide sequence using Pyoxim (1.1 equiv) and DIPEA (2.2 equiv) in DMF. Solvents, excess of reagents and soluble byproducts were removed by suction. The peptide was cleaved from the resin using 95% TFA, 2.5% TIS, 2.5% H_2O for 1 h and washed with DCM (4 × 1 min). The combined filtrates were collected into a round bottom flask and concentrated under reduced pressure. The fully deprotected peptide was then precipitated by adding cold Et_2O (dropwise) and the resulting precipitate was decanted and dried. Purification was conducted by semi-preparative HPLC using a 5-50% gradient over 17 min, with detection at 254 and 500 nm. Kinetex 150 × 10.0 mm (5 µm) C_{18} column was used, together with H_2O (0.1% HCOOH) and CH_3CN (0.1% HCOOH) as eluents and a flow rate of 5 mL min^{-1}. Pure fractions were collected and lyophilised to afford pure peptide **32** as a dark red solid (0.7 mg, 4% overall yield).

HPLC-MS: t_R: 3.1 min (>99% purity).

HR-MS (ESI+) m/z calcd. for $C_{73}H_{84}N_{20}O_9S$, $[M+2H]^{2+}$: 709.3298; found: 709.3318. $[M+3H]^{3+}$: 473.2231.

MALDI (m/z): $[M+H]^+$: 1417.6524; found: 1417.6524. $[M+Na]^+$: 1439.6556.

Computational Studies

Calculations were performed using the Gaussian 16, Revision A.03 package.[3] All structures were optimized at the TPSS[4] level of theory in combination with Grimme's D3 dispersion corrections with the Becke-Johnson damping scheme [D3(BJ)][5] in combination with a def2-TZVP basis set.[6] Analytical frequency calculations were carried out at the same level of theory in order to identify the stationary points either as intermediates (no imaginary frequencies) or transition states (only one imaginary frequency), as well as to provide thermal and non-thermal corrections to the free energy in gas-phase at 333 K and 1 atm. The electronic energy was then refined through PW6B95[7] single-point calculations on the optimized geometries in combination with a standalone version of Grimme's D4[8] dispersion corrections with a def2-QZVP basis set.[6] Solvent effects were included through the use of the implicit solvation model SMD[9] with a dielectric constant of $\varepsilon = 10.125$, which corresponds to 1,2-dichloroethane, the solvent of choice used in the experimental work. Unless otherwise stated, the energies herein provided are based on gas-phase Gibbs free energies with def2-TZVP basis set for which the electronic energies were improved at the PW6B95-D4/def2-QZVP+SMD(1,2-diChloroethane) level of theory.

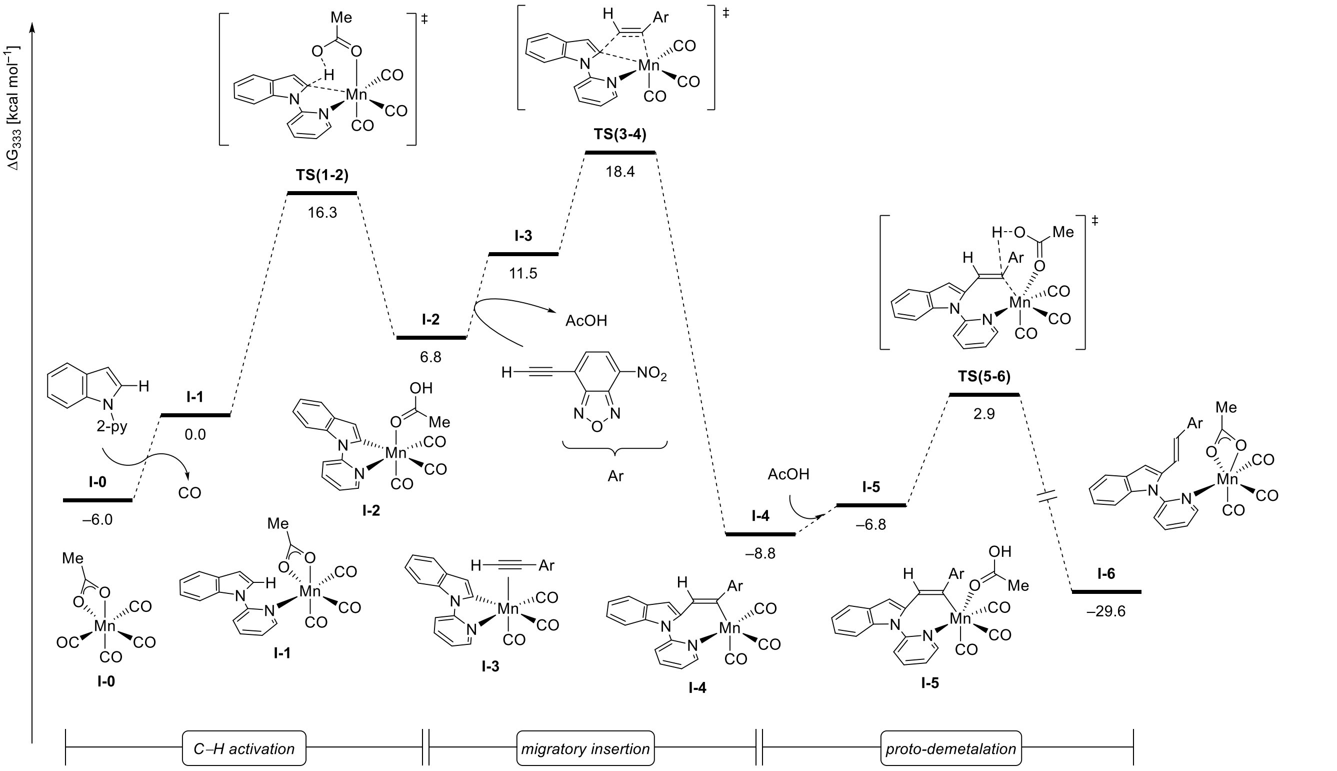

Figure S3. Computed Gibbs free energies (ΔG_{333}) in kcal·mol^{-1} between the C–H activation and proto-demetalation elementary steps at the PW6B95-D4/def2-QZVP+SMD(1,2-dichloroethane)//TPSS-D3(BJ)/def2-TZVP level of theory.

Based on previous reports[10] and preliminary computational studies, we obtained insights into the reaction mechanism through DFT calculations at the PW6B95-D4/def2-QZVP+SMD(1,2-dichloroethane)//TPSS-D3(BJ)/def2-TZVP level of theory (Figure S3). Since it is known that $MnBr(CO)_5$ can react with acetate and form $(CO)_4Mn(\kappa_2\text{-OAc})$ *via* $(CO)_5Mn(\kappa_1\text{-OAc})$ under an almost thermoneutral process,[10] we started with substrate coordinated complex **I-1**. The cyclometalated intermediate **I-2** is afforded *via* C–H activation with a barrier of 16.3 kcal·mol^{-1}. After ligand exchange, alkyne migratory insertion leads to the formation of intermediate **I-4** through TS(3-4) with a barrier of 18.4 kcal·mol^{-1}. Therefore, calculations indicate that alkyne insertion is the rate determining step. Then, subsequent proto-demetalation provides the desired product **I-6**. Although we tried to certify the role of BPh_3, we could not yet rationalize its role and further investigation is ongoing.[11]

Cartesian Coordinates and Energies

Intermediate 0

E[(PW6B95-D4/def2-QZVP+SMD(1,2-dichloroethane)]
= -1834.71254759 E_h
Total Gibbs Free Energy = -1834.6923458595 E_h
Lowest frequency = 30.11 cm^{-1}
Charge = 0, Multiplicity =1

16

Mn	1.618562	-0.523399	0.476798
C	0.106010	-1.477905	0.752223
O	-0.859372	-2.089257	0.912898
O	1.929134	0.107153	3.355723
O	3.278026	-3.018777	0.826235
C	2.657981	-2.066824	0.678054
C	1.800505	-0.143211	2.236659
O	1.907516	-0.652917	-1.674072
C	2.964279	0.063692	-1.587233
C	3.740179	0.444325	-2.815145
H	3.627957	-0.322294	-3.584222
H	3.340405	1.387258	-3.205299
H	4.792261	0.593268	-2.564683
O	3.329131	0.466817	-0.428701
C	0.616404	1.035811	0.218842
O	-0.013900	1.983473	0.086286

Intermediate 1

E[(PW6B95-D4/def2-QZVP+SMD(1,2-dichloroethane)]
= -2333.21789984 E_h
Total Gibbs Free Energy = -2333.0565120173 E_h
Lowest frequency = 32.66 cm^{-1}
Charge = 0, Multiplicity =1

39

C	0.800908	3.623824	0.518457
C	1.326743	2.345586	0.436167
C	-0.706228	1.452054	-0.226446
C	-1.302964	2.718127	-0.172192
C	-0.548449	3.813268	0.216392
H	1.440179	4.446995	0.815785
H	2.368519	2.144544	0.651744
H	-2.340851	2.815753	-0.466396
H	-0.997579	4.800436	0.257712
C	-2.790027	0.087601	-0.249777
C	-2.163103	-1.382331	-1.903364
H	-2.167469	-2.178026	-2.634179
N	-1.471773	0.357248	-0.641703
Mn	1.609142	-0.607527	0.440016
C	0.134840	-1.562102	0.847021
O	-0.756592	-2.221867	1.181235
O	1.909125	0.041156	3.300051
C	-3.240562	-1.012851	-1.026189
C	-4.529317	-1.519612	-0.801721
C	-5.322328	-0.932922	0.177159
H	-4.894975	-2.364003	-1.378221
C	-3.571544	0.665539	0.750901
C	-4.846903	0.142993	0.948334
H	-6.321145	-1.316369	0.361540
H	-3.202644	1.478556	1.367189
H	-5.480708	0.569657	1.719378
N	0.593479	1.256539	0.096753
O	3.210677	-3.060282	0.845174
C	2.589086	-2.100388	0.677730
C	1.783525	-0.214836	2.175578
O	1.923496	-0.703810	-1.723974
C	2.957013	0.032627	-1.593280
C	3.743888	0.482632	-2.793011
H	3.695329	-0.271457	-3.580463
H	3.301749	1.408566	-3.177953
H	4.779096	0.684635	-2.513693
O	3.294569	0.399900	-0.415522
C	-1.112060	-0.550987	-1.651436
H	-0.125313	-0.497720	-2.085824

TS(1-2)

E[(PW6B95-D4/def2-QZVP+SMD(1,2-dichloroethane)]
= -2333.19394022 E_h
Total Gibbs Free Energy = -2333.0304895281 E_h
Lowest frequency = -934.98 cm^{-1}
Charge = 0, Multiplicity =1

39

C	2.649389	0.143042	0.211058
C	2.808702	-1.215215	0.571949
C	4.086746	-1.776426	0.599298
C	5.167510	-0.994328	0.249878
C	4.989165	0.337190	-0.140939
C	3.736833	0.922927	-0.170261
C	0.578324	-0.754057	0.612962
C	1.506036	-1.740804	0.815190
H	4.220027	-2.814341	0.879156
H	6.165339	-1.414667	0.262822
H	5.850433	0.924570	-0.434488
H	3.626978	1.944063	-0.505881
H	-0.355118	-0.598123	1.535723
H	1.274365	-2.744223	1.138295
N	1.293723	0.411563	0.261524
C	0.606665	1.559181	-0.059835
C	-1.417306	2.421559	-0.748213
C	0.349297	3.922747	-0.238249
C	-0.957331	3.717831	-0.661016
H	-2.433611	2.206227	-1.049658
H	0.742914	4.926030	-0.131747
H	-1.611598	4.541908	-0.908061
N	-0.655390	1.360311	-0.474296
C	-1.790579	-2.365379	-0.313295
C	-2.716533	-0.376238	-1.669583
C	-0.269971	-1.075412	-1.912943
O	-2.055791	-3.464162	-0.157373
O	0.419736	-1.359226	-2.778548
O	-3.575958	-0.222569	-2.407396
Mn	-1.340280	-0.635868	-0.546825
O	-2.600770	-0.025718	1.049341
C	-2.209169	-0.025839	2.239816
C	-3.177741	0.377844	3.313228
H	-4.130595	0.674597	2.883228
H	-3.323252	-0.462412	3.993695

H -2.752655 1.195363 3.896314
O -1.043643 -0.348263 2.602409
C 1.140146 2.839055 0.077723
H 2.135776 2.968033 0.472562

Intermediate 2

E[(PW6B95-D4/def2-QZVP+SMD(1,2-dichloroethane)]
= -2333.20152828 E_h
Total Gibbs Free Energy = -2333.0456360719 E_h
Lowest frequency = 22.28 cm^{-1}
Charge = 0, Multiplicity =1

39

C 2.541912 0.067398 0.331581
C 2.576468 -1.355271 0.364949
C 3.778356 -2.013534 0.651308
C 4.924006 -1.260000 0.889031
C 4.880764 0.139645 0.835306
C 3.694879 0.821652 0.555121
C 0.415185 -0.755741 -0.164268
C 1.253666 -1.827844 0.048773
H 3.812469 -3.098633 0.678573
H 5.863142 -1.757777 1.110419
H 5.786194 0.712512 1.009849
H 3.712940 1.901737 0.501483
H -0.288519 -1.321327 1.541755
H 0.959177 -2.866212 -0.031398
N 1.213254 0.417321 0.021986
C 0.598964 1.639426 -0.142282
C -1.387654 2.684685 -0.775504
C 0.452521 4.039728 -0.090172
C -0.860985 3.946871 -0.554182
H -2.404510 2.551578 -1.122984
H 0.901522 5.006986 0.112168
H -1.467930 4.825812 -0.735511
N -0.689715 1.548987 -0.578804
C -1.918551 -2.083034 -0.732003
C -3.106500 0.151633 -1.411698
C -0.839896 -0.544861 -2.436293
O -2.174357 -3.214212 -0.706494
O -0.414565 -0.684420 -3.505465
O -4.132956 0.420004 -1.879858
Mn -1.459948 -0.343095 -0.775239
O -2.174916 0.014597 1.175347
C -1.818786 -0.456799 2.263882
C -2.569770 -0.172143 3.532092
H -3.403014 0.497764 3.327338
H -2.938979 -1.113182 3.951192
H -1.894248 0.271790 4.268827
O -0.771329 -1.246768 2.423277
C 1.188634 2.884649 0.122228
H 2.195277 2.939380 0.505869

Intermediate 3

E[(PW6B95-D4/def2-QZVP+SMD(1,2-dichloroethane)]
= -2801.22106953 E_h
Total Gibbs Free Energy = -2801.0462156598 E_h
Lowest frequency = 10.99 cm^{-1}
Charge = 0, Multiplicity =1

48

C 2.808523 -1.158829 -2.991997
C 2.402565 -1.448026 -1.700225
C 1.163041 0.524048 -1.541127
C 1.515487 0.866645 -2.856934
C 2.341040 0.020330 -3.578255
H 3.466902 -1.839931 -3.518733
H 2.724185 -2.353971 -1.200235
H 1.134777 1.771725 -3.303699
H 2.619235 0.280771 -4.594961
C -0.196834 2.563494 -0.850278
C -0.731676 1.669568 1.217016
H -1.139660 1.558787 2.212679
N 0.374869 1.279288 -0.706035
Mn 0.844436 -1.088210 0.887149
C -0.015211 -1.328425 2.462658
O -0.577025 -1.445861 3.464040
C 2.058982 -0.007990 1.687713
O 2.815870 0.696679 2.198457
C 1.915586 -2.544408 1.168477
O 2.633112 -3.427006 1.380231
C 0.041924 0.744753 0.574189
C -0.904820 2.814141 0.358732
C -1.584598 4.027059 0.521262
C -1.549009 4.967572 -0.504277
H -2.127161 4.229131 1.440494
C -0.149460 3.511701 -1.871893
C -0.836042 4.713268 -1.682993
H -2.072592 5.912244 -0.389589
H 0.405398 3.367047 -2.789305
H -0.809121 5.462020 -2.468880
N 1.592115 -0.644249 -0.983379
C -1.049621 -1.467778 -0.028857
H -1.908572 -0.826598 -0.038324
C -0.458411 -2.539539 -0.253814
C -0.072572 -3.826839 -0.699945
C 0.289941 -4.084915 -2.016307
C -0.018722 -4.945399 0.195764
C 0.701837 -5.370186 -2.444193
H 0.261288 -3.275273 -2.736665
C 0.400301 -6.254763 -0.225454
C 0.769524 -6.444383 -1.588265
H 0.979364 -5.533369 -3.479899
N 1.214019 -7.744154 -2.098805
O 1.241501 -8.679384 -1.297718
O 1.529596 -7.810499 -3.293665
N -0.321073 -4.976973 1.483740
O -0.093529 -6.275904 1.852974
N 0.349292 -7.077275 0.816119

TS(3-4)

E[(PW6B95-D4/def2-QZVP+SMD(1,2-dichloroethane)]
= -2801.22127620 E_h
Total Gibbs Free Energy = -2801.0352892855 E_h
Lowest frequency = -266.46 cm^{-1}
Charge = 0, Multiplicity =1

48

C -0.025695 2.679285 2.240369

C	0.042014	1.334633	1.948916
C	-1.869714	1.417532	0.665661
C	-1.983991	2.791872	0.873268
C	-1.056302	3.418978	1.676172
H	0.721188	3.132706	2.876384
H	0.846376	0.725313	2.337667
H	-2.761792	3.352800	0.382227
H	-1.128062	4.485617	1.848772
C	-4.024205	0.929973	-0.535679
C	-3.358556	-1.147102	-1.238745
H	-3.344256	-2.115089	-1.715436
N	-2.725343	0.678058	-0.106802
Mn	-0.710285	-1.277599	0.527615
C	-0.647334	-2.934977	-0.173076
O	-0.630434	-3.980905	-0.625871
C	-1.915901	-1.832180	1.746378
O	-2.710045	-2.168971	2.491047
C	0.685656	-1.662441	1.612271
O	1.555199	-1.898556	2.312598
C	-2.325014	-0.608668	-0.519245
C	-4.433901	-0.214357	-1.260495
C	-5.718798	-0.259676	-1.804125
C	-6.567734	0.810529	-1.607606
H	-6.043245	-1.129732	-2.361691
C	-4.890230	1.992846	-0.312943
C	-6.160083	1.918625	-0.861347
H	-7.567470	0.788476	-2.023172
H	-4.622861	2.848757	0.288002
H	-6.850654	2.736436	-0.698393
N	-0.853892	0.707034	1.182941
C	-0.591237	-0.521133	-1.466223
H	-1.002867	-0.667989	-2.446151
C	0.516361	-0.305594	-0.894153
C	1.842139	0.157841	-0.856675
C	2.171092	1.497917	-0.932301
C	2.947465	-0.735745	-0.687973
C	3.502491	1.949809	-0.839416
H	1.379276	2.224927	-1.052804
C	4.300030	-0.289137	-0.594931
C	4.562758	1.105604	-0.668666
H	3.714467	3.009203	-0.898362
N	5.900786	1.644946	-0.569100
O	6.804338	0.851864	-0.422354
O	6.029640	2.853010	-0.637467
N	2.935883	-2.043241	-0.611749
O	4.217661	-2.393264	-0.471737
N	5.068033	-1.347295	-0.460113

Intermediate 4

E[(PW6B95-D4/def2-QZVP+SMD(1,2-dichloroethane)] = -2801.25569762 E_h
Total Gibbs Free Energy = -2801.0785599340 E_h
Lowest frequency = 14.56 cm^{-1}
Charge = 0, Multiplicity =1

48

C	-0.859441	3.494792	1.705483
C	-0.624208	2.127542	1.632063
C	-2.007963	1.932462	-0.211580
C	-2.282889	3.293978	-0.212258
C	-1.705228	4.091235	0.772097
H	-0.379152	4.075360	2.484769
H	0.030903	1.633521	2.338543
H	-2.923310	3.704256	-0.983995
H	-1.900494	5.158072	0.799969
C	-3.866217	0.493710	-0.903839
C	-2.448699	-1.215138	-1.511178
H	-2.090583	-2.161503	-1.889858
N	-2.579700	1.037951	-1.148918
Mn	-0.858360	-0.736357	0.529391
C	-0.546105	-2.491246	0.235946
O	-0.378947	-3.620762	0.057597
C	-2.296854	-1.101358	1.603298
O	-3.186201	-1.356213	2.298577
C	0.305358	-0.778251	1.875932
O	1.089046	-0.816310	2.732005
C	-1.720681	-0.035117	-1.521279
C	-3.814106	-0.895934	-1.158403
C	-4.970946	-1.669477	-1.008238
C	-6.138186	-1.042597	-0.583965
H	-4.950824	-2.737066	-1.202863
C	-5.019792	1.123434	-0.450980
C	-6.158570	0.332419	-0.300011
H	-7.045640	-1.624133	-0.457993
H	-5.040536	2.184464	-0.225793
H	-7.080142	0.791696	0.043255
N	-1.194061	1.337171	0.699122
C	-0.308142	0.219743	-1.884803
H	-0.045220	0.659995	-2.850202
C	0.515404	-0.158712	-0.895269
C	1.940089	0.065381	-0.828825
C	2.499898	1.321535	-1.036957
C	2.870358	-0.953614	-0.435837
C	3.883102	1.580205	-0.882847
H	1.844205	2.141242	-1.311345
C	4.281595	-0.712259	-0.275265
C	4.776764	0.603131	-0.509720
H	4.274275	2.575931	-1.059618
N	6.191589	0.938840	-0.365390
O	6.955986	0.027114	-0.041283
O	6.528494	2.112364	-0.578186
N	2.619876	-2.226723	-0.171004
O	3.839315	-2.759981	0.147551
N	4.876455	-1.842296	0.086173

Intermediate 5

E[(PW6B95-D4/def2-QZVP+SMD(1,2-dichloroethane)] = -3030.75588096 E_h
Total Gibbs Free Energy = -3030.5285921952 E_h
Lowest frequency = 22.22 cm^{-1}
Charge = 0, Multiplicity =1

56

C	-2.503284	4.074958	-0.890735
C	-1.609980	3.302949	-0.169455
C	-2.533836	1.332005	-0.930249
C	-3.459381	2.047544	-1.703531
C	-3.452190	3.431547	-1.685029
H	-2.446064	5.155487	-0.827881
H	-0.862163	3.772483	0.455389
H	-4.157817	1.489492	-2.315378
H	-4.158393	3.994739	-2.286377

C	-3.841804	-0.746669	-0.891626
C	-2.237592	-2.180268	-1.671298
H	-1.714739	-3.062050	-2.014277
N	-2.616131	-0.070272	-0.953567
Mn	-0.065066	1.026261	1.078894
C	-0.764475	1.913923	2.524814
O	-1.166941	2.508070	3.431799
O	1.826196	3.207829	0.518884
C	-1.629904	-0.969246	-1.412231
C	-3.622045	-2.079477	-1.330037
C	-4.691752	-2.989848	-1.302444
C	-5.931157	-2.557248	-0.846259
H	-4.547766	-4.016408	-1.625983
C	-5.080782	-0.309992	-0.423196
C	-6.123818	-1.233676	-0.408907
H	-6.765765	-3.251014	-0.818603
H	-5.228854	0.703758	-0.065953
H	-7.099141	-0.926967	-0.044657
N	-1.605893	1.946766	-0.167867
C	-0.214346	-0.683122	-1.510719
H	0.272808	-1.257960	-2.302319
C	0.553152	0.040679	-0.662845
C	1.999206	0.010525	-0.875211
C	2.754780	0.892074	-1.630767
C	2.758198	-1.004023	-0.209320
C	4.167937	0.795883	-1.718277
H	2.256374	1.692442	-2.166247
C	4.187220	-1.127226	-0.277356
C	4.894633	-0.175873	-1.067918
H	4.720689	1.507761	-2.321405
N	6.345977	-0.215510	-1.207965
O	6.941710	-1.111328	-0.602696
O	6.881082	0.644416	-1.920289
N	2.282305	-1.971533	0.554152
O	3.382718	-2.683413	0.959765
N	4.564215	-2.174317	0.449427
O	2.006661	-0.005493	2.913947
C	1.195625	0.348035	2.163898
C	1.062157	2.356777	0.729215
O	-1.420239	-0.561821	1.505310
C	-1.371038	-1.792265	1.630949
C	-2.587646	-2.569956	2.047288
H	-3.475734	-1.948160	1.951048
H	-2.683915	-3.470579	1.438826
H	-2.465611	-2.879066	3.091161
O	-0.305853	-2.553962	1.461046
H	0.519475	-2.088869	1.146369

TS(5-6)

E[(PW6B95-D4/def2-QZVP+SMD(1,2-dichloroethane)]
= -3030.74641374 E_h
Total Gibbs Free Energy = -3030.5132081518 E_h
Lowest frequency = -1161.51 cm^{-1}
Charge = 0, Multiplicity =1

56

C	-2.240647	3.862026	-1.407690
C	-1.383619	3.153385	-0.598220
C	-2.438221	1.171307	-1.021208
C	-3.345292	1.815135	-1.862417
C	-3.255976	3.174711	-2.054353
H	-2.107842	4.928958	-1.521906
H	-0.586629	3.667198	-0.080805
H	-4.096081	1.221023	-2.363999
H	-3.950275	3.684509	-2.710355
C	-3.795455	-0.839411	-0.805700
C	-2.333947	-2.279999	-1.788178
H	-1.875043	-3.167320	-2.194611
N	-2.555887	-0.222855	-0.891491
Mn	-0.133233	1.058596	1.106703
C	-0.631383	2.369829	2.222880
O	-0.934226	3.204810	2.938762
O	2.078439	2.741261	0.165283
C	-1.663687	-1.129051	-1.483241
C	-3.678847	-2.138493	-1.338491
C	-4.783600	-2.993454	-1.297554
C	-5.962192	-2.529277	-0.750753
H	-4.711699	-4.002616	-1.684124
C	-4.979945	-0.366688	-0.256520
C	-6.059728	-1.229228	-0.237231
H	-6.827018	-3.179648	-0.709506
H	-5.048985	0.627270	0.166554
H	-6.994461	-0.898288	0.197749
N	-1.472175	1.830240	-0.377597
C	-0.239042	-0.909886	-1.559311
H	0.234750	-1.255378	-2.476932
C	0.524815	-0.520041	-0.513865
C	1.965140	-0.485944	-0.738679
C	2.536746	0.163055	-1.806865
C	2.892914	-1.124091	0.140822
C	3.934437	0.216801	-2.015966
H	1.893995	0.692410	-2.498717
C	4.308834	-1.084741	-0.051749
C	4.822643	-0.376316	-1.171041
H	4.333010	0.753381	-2.866664
N	6.245421	-0.272476	-1.434253
O	6.990899	-0.798010	-0.638867
O	6.591631	0.331695	-2.430668
N	2.646360	-1.851739	1.204092
O	3.844062	-2.232266	1.652904
N	4.873487	-1.782917	0.907047
O	1.580675	0.145382	3.321990
C	0.934371	0.442828	2.434558
C	1.218713	2.066108	0.505073
O	-1.707394	-0.017110	1.821477
C	-1.628298	-1.273905	1.854715
C	-2.754661	-2.042057	2.474318
H	-3.336891	-1.405708	3.135976
H	-3.403258	-2.403172	1.672611
H	-2.367098	-2.908765	3.006650
O	-0.663468	-1.915830	1.366487
H	-0.015468	-1.075577	0.586978

Intermediate 6

E[(PW6B95-D4/def2-QZVP+SMD(1,2-dichloroethane)]
= -3030.78988140 E_h
Total Gibbs Free Energy = -3030.5649965393 E_h
Lowest frequency = 10.40 cm^{-1}
Charge = 0, Multiplicity =1

56

C	0.748058	1.088789	3.491304

C	1.044041	1.566814	2.222310
C	1.522370	-0.556171	1.454940
C	1.251314	-1.106460	2.705178
C	0.845190	-0.275967	3.743019
H	0.443131	1.787654	4.261718
H	0.966434	2.618389	1.979795
H	1.359538	-2.176947	2.834397
H	0.616737	-0.684978	4.721568
C	3.309024	-1.807627	0.331773
C	2.063449	-3.177831	-1.002155
H	1.750963	-3.928118	-1.715054
N	1.973989	-1.421873	0.424207
Mn	1.780667	1.776397	-0.680949
C	2.539571	0.459771	-1.635193
O	3.013486	-0.302823	-2.368803
O	4.421301	2.609663	0.346217
C	1.198208	-2.284537	-0.378116
C	3.389212	-2.901043	-0.575532
C	4.652680	-3.446729	-0.871997
C	5.771724	-2.904093	-0.260445
H	4.744706	-4.272897	-1.570016
C	4.429899	-1.257355	0.950958
C	5.661757	-1.823658	0.640465
H	6.754404	-3.309568	-0.479056
H	4.351260	-0.414244	1.628871
H	6.560060	-1.417965	1.094721
N	1.416443	0.764906	1.195273
C	-0.215345	-2.222528	-0.548658
H	-0.595118	-2.978536	-1.232010
C	-1.084902	-1.352199	0.032616
C	-2.501835	-1.297491	-0.193511
C	-3.226326	-2.012158	-1.145380
C	-3.286447	-0.401972	0.613158
C	-4.619268	-1.864677	-1.305650
H	-2.710382	-2.696607	-1.809931
C	-4.707367	-0.230510	0.459491
C	-5.372402	-0.997954	-0.538612
H	-5.141423	-2.437757	-2.063588
N	-6.806333	-0.890203	-0.768630
O	-7.429681	-0.088886	-0.065177
O	-7.304278	-1.605742	-1.649973
N	-2.860577	0.380567	1.591615
O	-3.986988	1.029033	2.032591
N	-5.134698	0.663497	1.343976
O	2.150568	3.491895	-3.059434
C	1.991962	2.819030	-2.133943
C	3.383496	2.291178	-0.062678
O	-0.307389	1.467582	-1.109378
C	-0.608681	2.554220	-0.511362
C	-2.025047	3.051198	-0.454214
H	-2.691711	2.370996	-0.984355
H	-2.341318	3.143529	0.588192
H	-2.074109	4.046184	-0.906122
O	0.338170	3.225349	0.033887
H	-0.719142	-0.606134	0.723500

1-(pyridin-2-yl)-1*H*-indole

E[(PW6B95-D4/def2-QZVP+SMD(1,2-dichloroethane)]
= -612.002840619 E_h
Total Gibbs Free Energy = -611.8857668184 E_h
Lowest frequency = 39.29 cm^{-1}
Charge = 0, Multiplicity =1

25

C	3.960876	-0.755877	0.002147
C	2.849105	-1.544410	-0.274426
C	1.395305	0.213922	-0.026138
C	2.447301	1.090357	0.298097
C	3.741475	0.591062	0.299363
H	4.958859	-1.181456	-0.003032
H	2.959787	-2.604498	-0.495332
H	2.256277	2.122696	0.566423
H	4.571866	1.246588	0.545951
C	-1.112265	-0.083864	-0.013092
C	-1.646575	2.153298	-0.155872
H	-2.193349	3.082811	-0.231914
N	0.069851	0.676420	-0.075653
C	-2.200763	0.831732	-0.052904
C	-3.515495	0.348732	0.014479
C	-3.722173	-1.020295	0.125343
H	-4.354887	1.038121	-0.016844
C	-1.317512	-1.461578	0.102003
C	-2.634266	-1.909498	0.171246
H	-4.734396	-1.411404	0.177187
H	-0.482106	-2.147119	0.118472
H	-2.821537	-2.976213	0.256644
N	1.592274	-1.081200	-0.298057
C	-0.289558	2.026517	-0.162884
H	0.471877	2.783351	-0.265762

AcOH

E[(PW6B95-D4/def2-QZVP+SMD(1,2-dichloroethane)]
= -229.474084076 E_h
Total Gibbs Free Energy = -229.4532199065 E_h
Lowest frequency = 61.80 cm^{-1}
Charge = 0, Multiplicity =1

8

C	-0.902778	-0.352698	-0.009768
C	0.381771	0.422686	-0.143886
O	1.423874	-0.238958	0.445147
O	0.520395	1.494122	-0.692367
H	-0.791860	-1.337614	-0.474077
H	-1.130451	-0.513105	1.048720
H	-1.710970	0.199080	-0.488168
H	2.210018	0.326287	0.314099

NBD-alkyne

E[(PW6B95-D4/def2-QZVP+SMD(1,2-dichloroethane)]
= -697.498060765 E_h
Total Gibbs Free Energy = -697.4612651842 E_h
Lowest frequency = 38.29 cm^{-1}
Charge = 0, Multiplicity =1

17

C	-0.246715	1.572702	-0.026717
C	-1.399642	0.825903	-0.014793
C	-1.381451	-0.591189	0.009737
C	-0.197600	-1.314169	0.023301

C	1.010874	-0.541959	0.011021
C	1.006692	0.896912	-0.013889
N	2.263057	-0.973048	0.019630
O	3.014586	0.171530	0.000517
N	2.261568	1.331268	-0.020249
C	-0.165820	-2.726863	0.047772
C	-0.132665	-3.935699	0.068718
N	-0.337882	3.038690	-0.052132
O	0.723498	3.662540	-0.062028
O	-1.468443	3.538575	-0.061704
H	-2.345757	1.355886	-0.024823
H	-2.324271	-1.126821	0.018156
H	-0.098428	-5.001258	0.087186

CO

E[(PW6B95-D4/def2-QZVP+SMD(1,2-dichloroethane)]
= -113.494457331 E_h
Total Gibbs Free Energy = -113.5120759601 E_h
Lowest frequency = 2139.77 cm^{-1}
Charge = 0, Multiplicity =1

2

C	-0.567281	0.000000	0.000000
O	0.567281	0.000000	0.000000

Reference

[1] a) N. Kaplaneris, J. Son, L. Mendive-Tapia, A. Kopp, N. D. Barth, I. Maksso, M. Vendrell, L. Ackermann, *Nat. Commun.,* **2021**, *12*, 3389; b) A. Schischko, H. Ren, N. Kaplaneris, L. Ackermann, *Angew. Chem. Int. Ed.,* **2017**, *56*, 1576.

[2] A. M. Brouwer, *Pure Appl. Chem.,* **2011**, *83*, 2213.

[3] M. J. Frisch, G. W. Trucks, H. B. Schlegel, G. E. Scuseria, M. A. Robb, J. R. Cheeseman, G. Scalmani, V. Barone, G. A. Petersson, H. Nakatsuji, X. Li, M. Caricato, A. V. Marenich, J. Bloino, B. G. Janesko, R. Gomperts, B. Mennucci, H. P. Hratchian, J. V. Ortiz, A. F. Izmaylov, J. L. Sonnenberg, Williams, F. Ding, F. Lipparini, F. Egidi, J. Goings, B. Peng, A. Petrone, T. Henderson, D. Ranasinghe, V. G. Zakrzewski, J. Gao, N. Rega, G. Zheng, W. Liang, M. Hada, M. Ehara, K. Toyota, R. Fukuda, J. Hasegawa, M. Ishida, T. Nakajima, Y. Honda, O. Kitao, H. Nakai, T. Vreven, K. Throssell, J. A. Montgomery Jr., J. E. Peralta, F. Ogliaro, M. J. Bearpark, J. J. Heyd, E. N. Brothers, K. N. Kudin, V. N. Staroverov, T. A. Keith, R. Kobayashi, J. Normand, K. Raghavachari, A. P. Rendell, J. C. Burant, S. S. Iyengar, J. Tomasi, M. Cossi, J. M. Millam, M. Klene, C. Adamo, R. Cammi, J. W. Ochterski, R. L. Martin, K. Morokuma, O. Farkas, J. B. Foresman, D. J. Fox, Gaussian16 Rev.A.03: Wallingford, CT, **2016**.

[4] J. Tao, J. P. Perdew, V. N. Staroverov, G. E. Scuseria, *Phys. Rev. Lett.,* **2003**, *91*, 146401.

[5] a) S. Grimme, S. Ehrlich, L. Goerigk, *J. Comput. Chem.,* **2011**, *32*, 1456; b) S. Grimme, J. Antony, S. Ehrlich, H. Krieg, *J. Chem. Phys.,* **2010**, *132*, 154104.

[6] a) F. Weigend, *Phys. Chem. Chem. Phys.,* **2006**, *8*, 1057; b) F. Weigend, R. Ahlrichs, *Phys. Chem. Chem. Phys.,* **2005**, *7*, 3297.

[7] Y. Zhao, D. G. Truhlar, *J. Phys. Chem. A,* **2005**, *109*, 5656.

[8] a) E. Caldeweyher, S. Ehlert, A. Hansen, H. Neugebauer, S. Spicher, C. Bannwarth, S. Grimme, *J. Chem. Phys.,* **2019**, *150*, 154122; b) E. Caldeweyher, C. Bannwarth, S. Grimme, *J. Chem. Phys.,* **2017**, *147*, 034112; c) https://www.chemie.uni-bonn.de/pctc/mulliken-center/software/dftd4.

[9] A. V. Marenich, C. J. Cramer, D. G. Truhlar, *J. Phys. Chem. B,* **2009**, *113*, 6378.

[10] a) N. Kaplaneris, T. Rogge, R. Yin, H. Wang, G. Sirvinskaite, L. Ackermann, *Angew. Chem. Int. Ed.,* **2019**, *58*, 3476; b) X. Ma, Y. Dang, *J. Org. Chem.,* **2019**, *84*, 1916; c) C. Wang, B. Maity, L. Cavallo, M. Rueping, *Org. Lett.,* **2018**, *20*, 3105.

[11] a) T. Liu, Y. Hu, Y. Yang, C. Wang, *CCS Chem.,* **2020**, *2*, 749; b) Z. Ruan, N. Sauermann, E. Manoni, L. Ackermann, *Angew. Chem. Int. Ed.,* **2017**, *56*, 3172; c) S. Sueki, Z. Wang, Y. Kuninobu, *Org. Lett.,* **2016**, *18*, 304.

^{1}H NMR and ^{13}C NMR Spectra

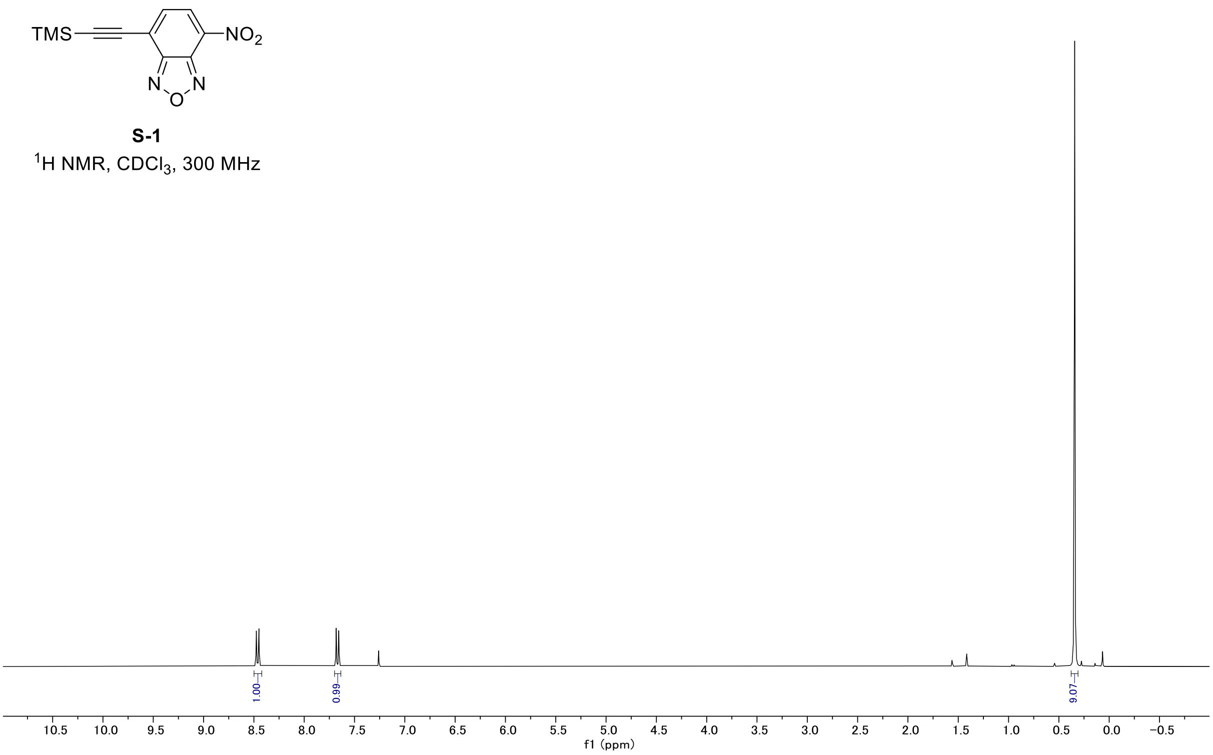

S-1

^{1}H NMR, $CDCl_3$, 300 MHz

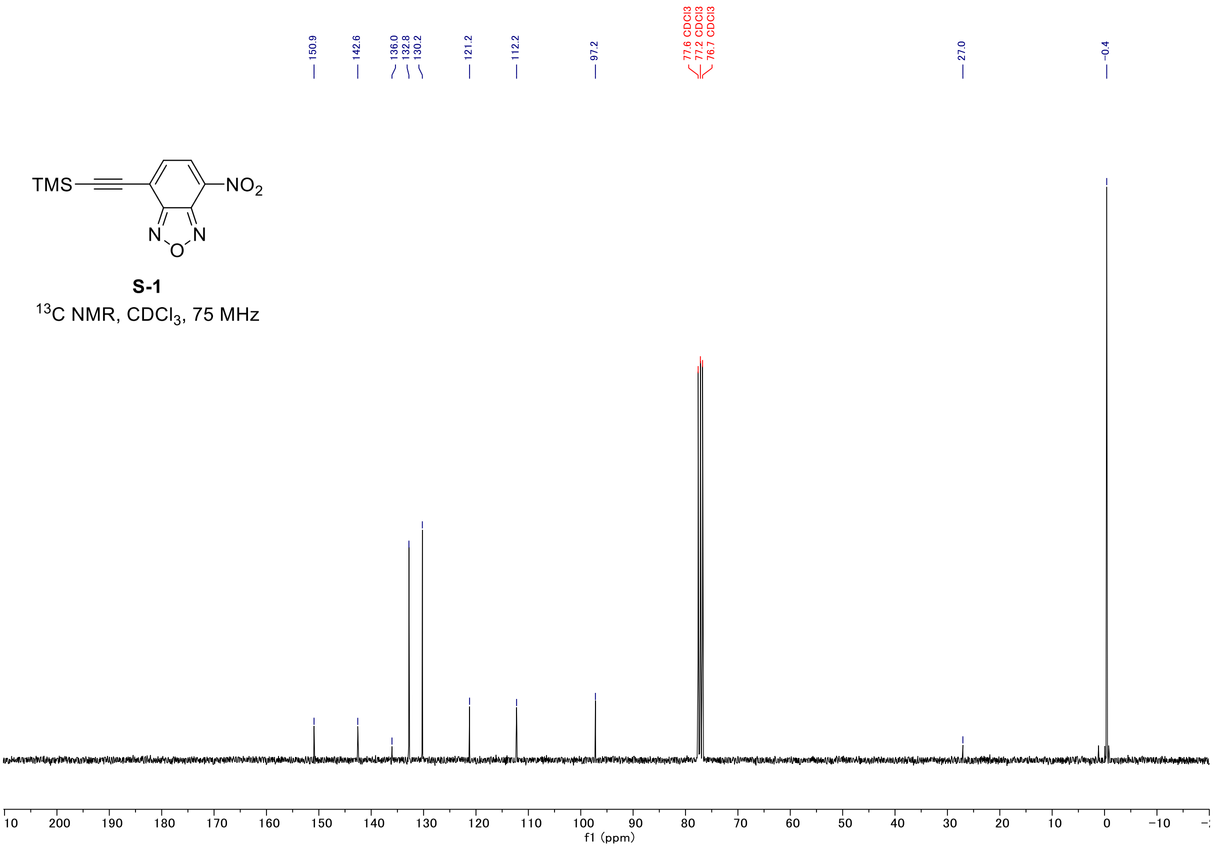

S-1

^{13}C NMR, $CDCl_3$, 75 MHz

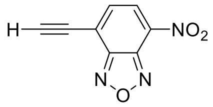

2

^{1}H NMR, $CDCl_3$, 300 MHz

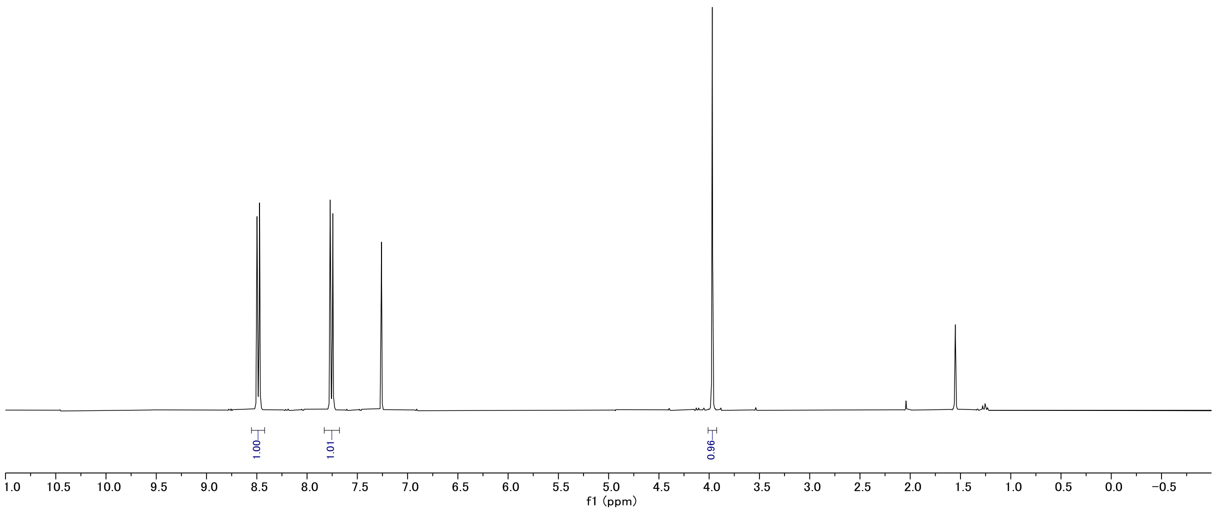

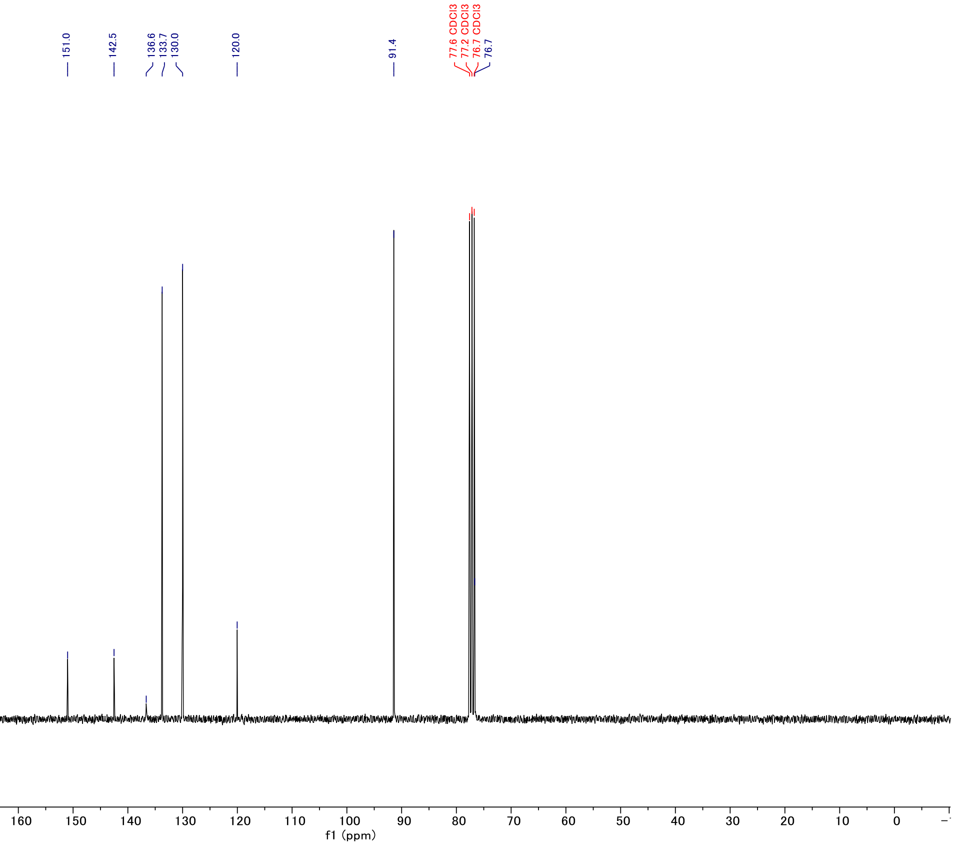

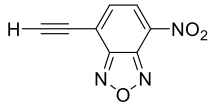

2

^{13}C NMR, $CDCl_3$, 75 MHz

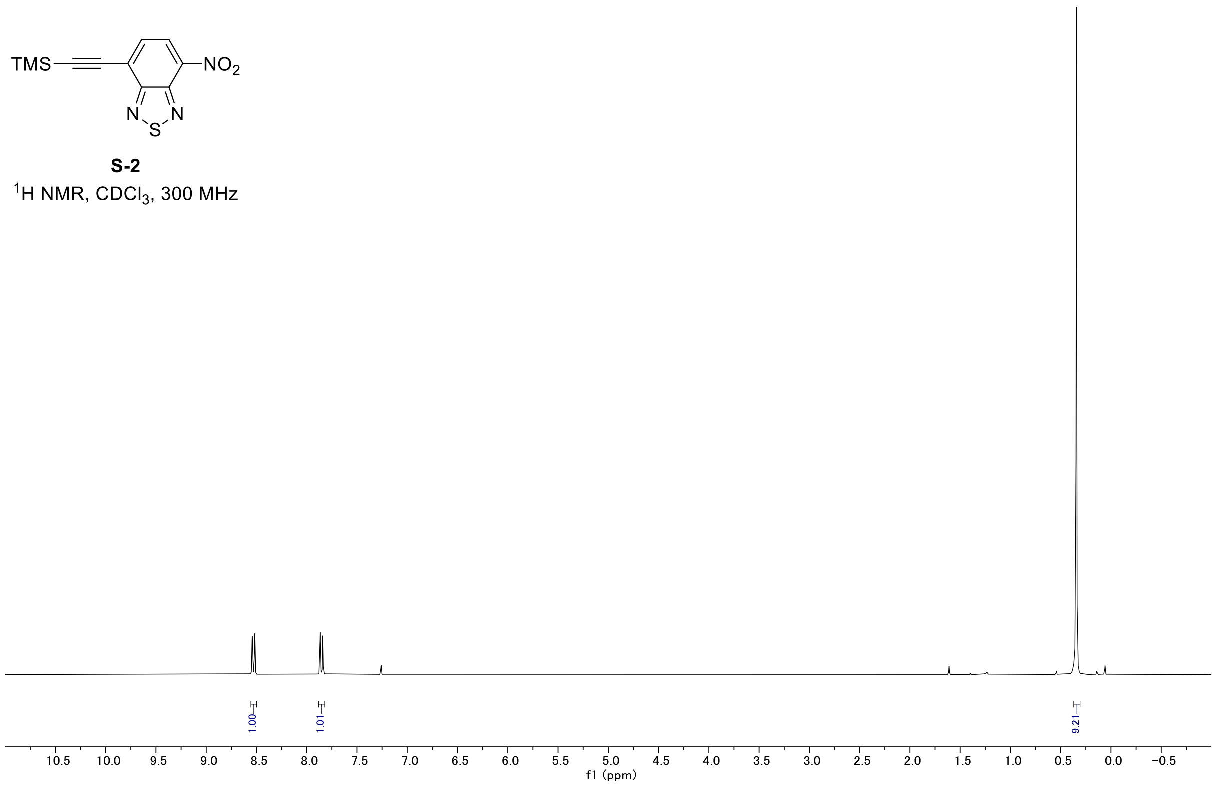

S-2

^{1}H NMR, $CDCl_3$, 300 MHz

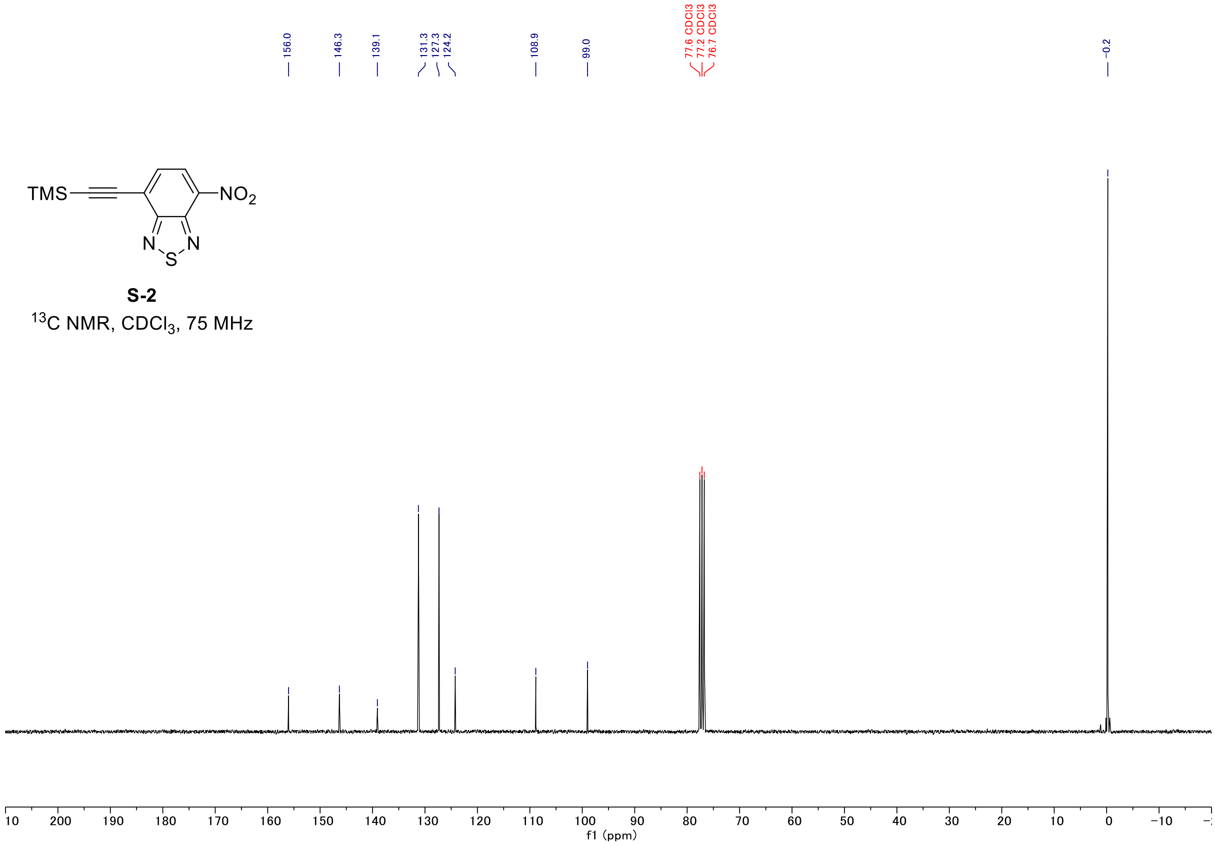

S-2

^{13}C NMR, $CDCl_3$, 75 MHz

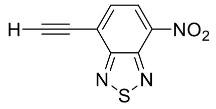

S-3

^{1}H NMR, $CDCl_3$, 400 MHz

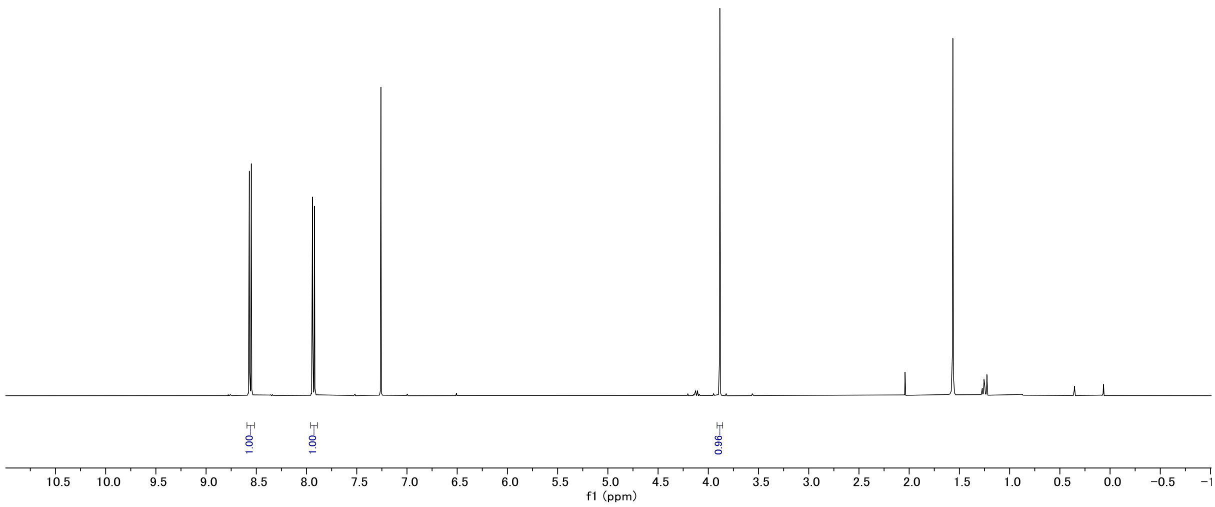

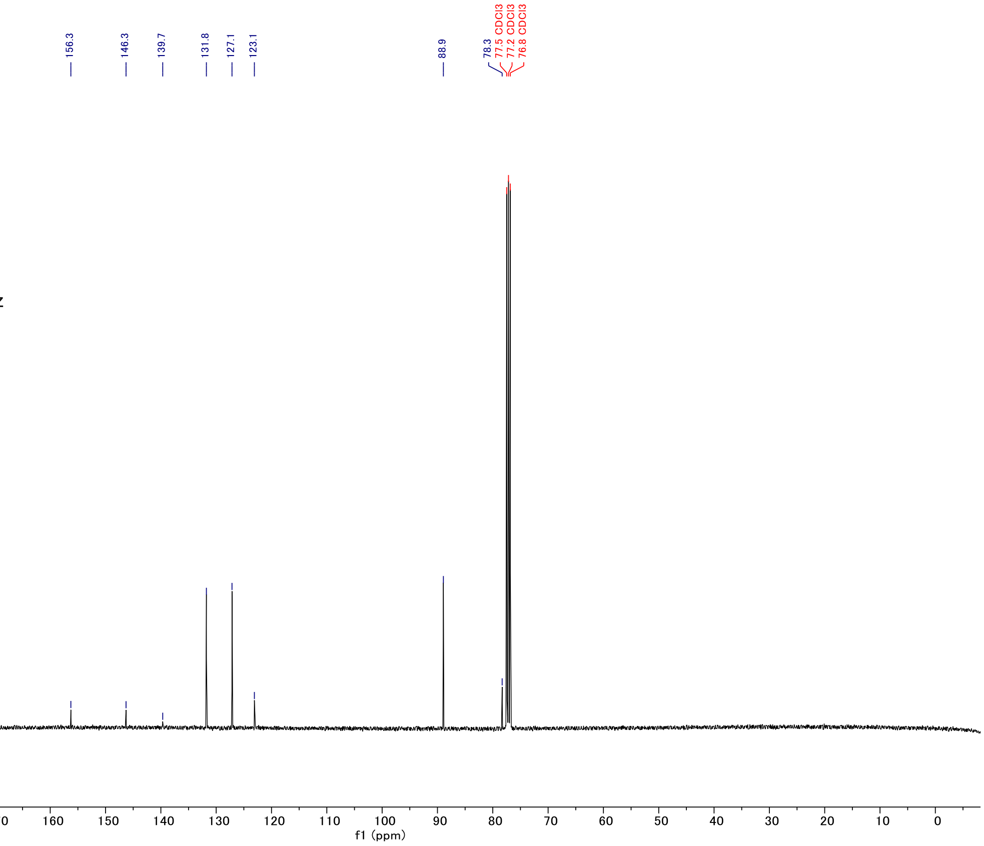

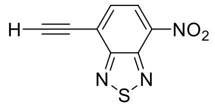

S-3

^{13}C NMR, $CDCl_3$, 101 MHz

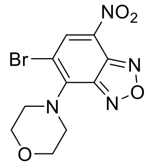

S-4

^{1}H NMR, $CDCl_3$, 400 MHz

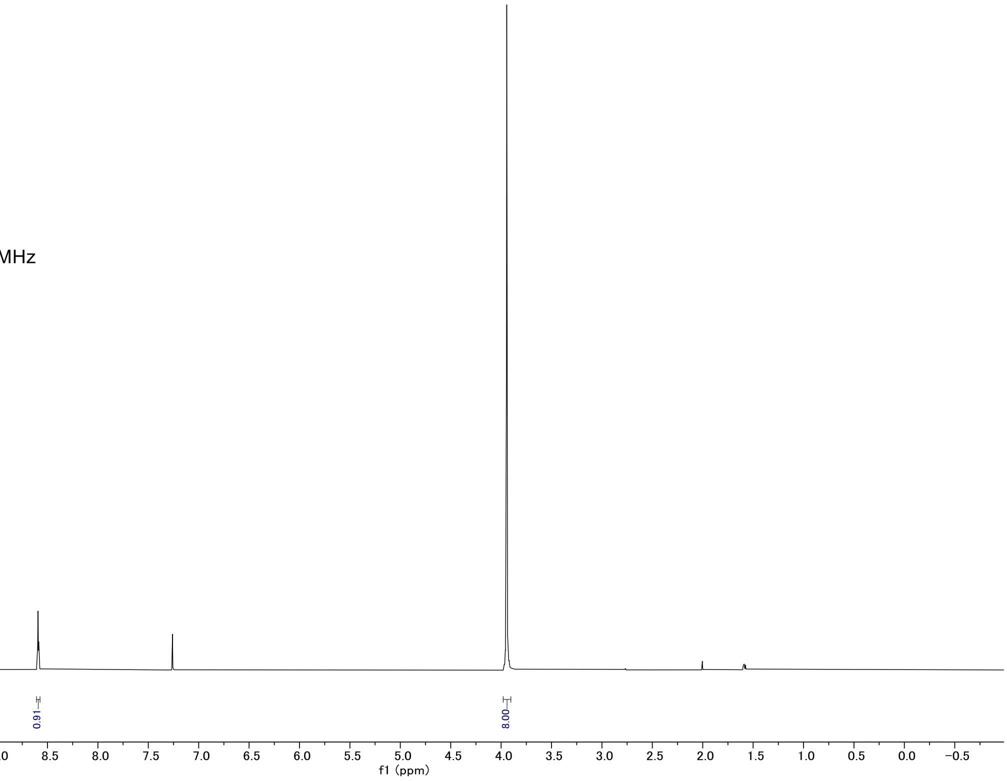

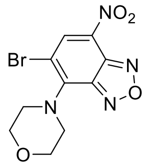

S-4

^{13}C NMR, $CDCl_3$, 101 MHz

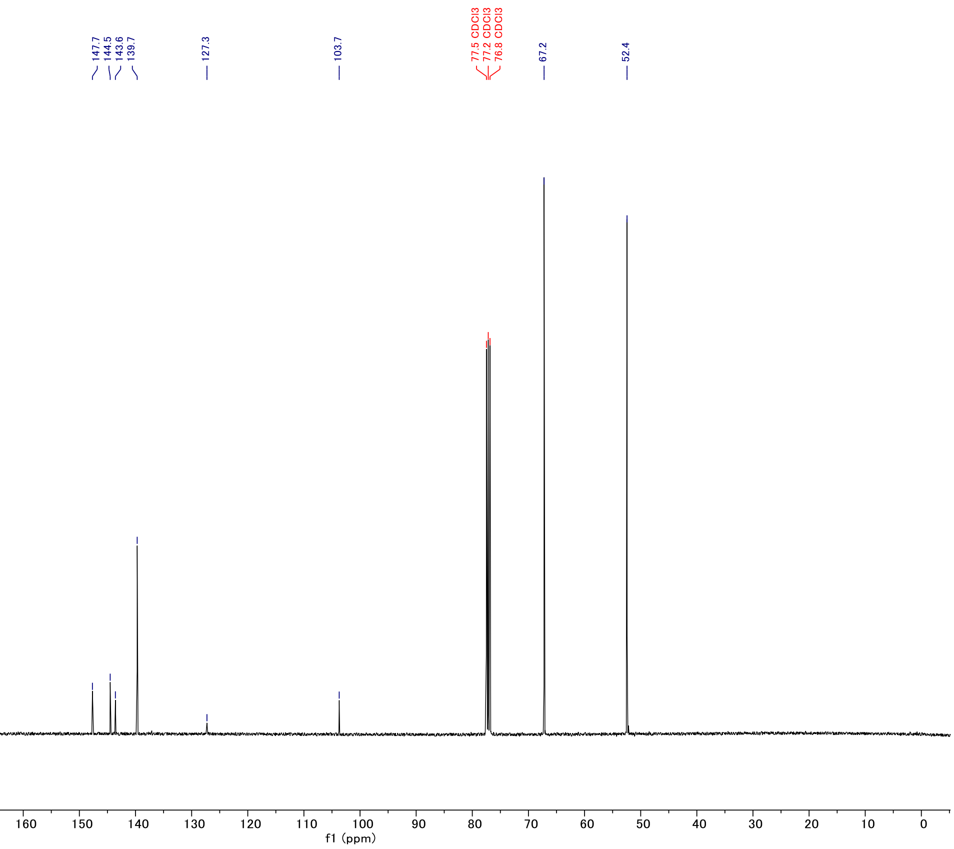

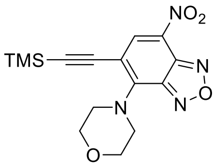

S-5

^{1}H NMR, $CDCl_3$, 300 MHz

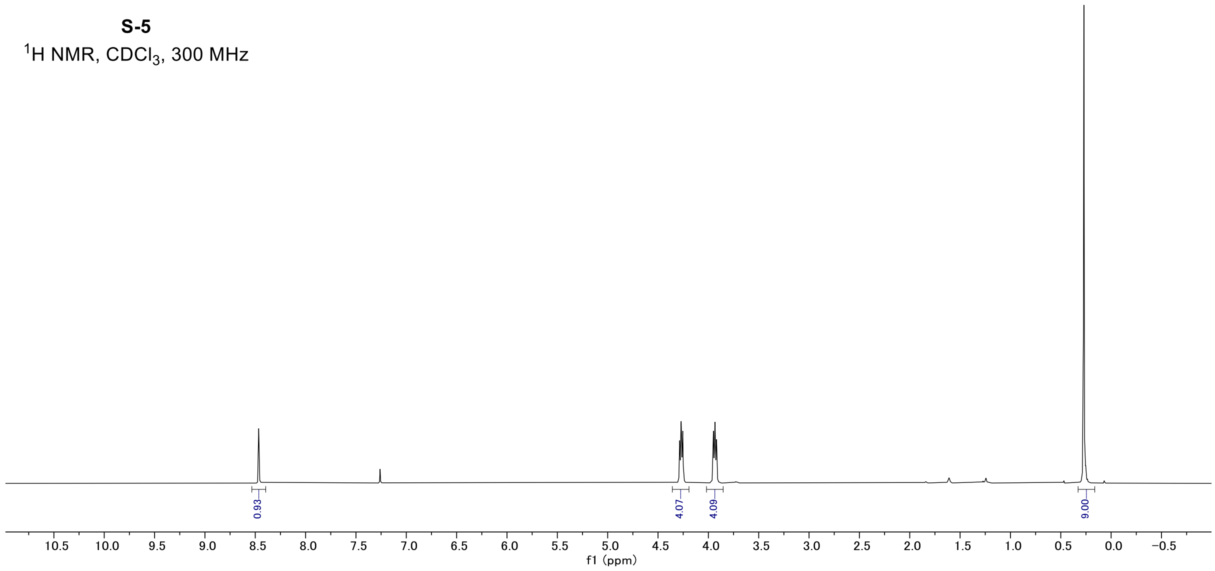

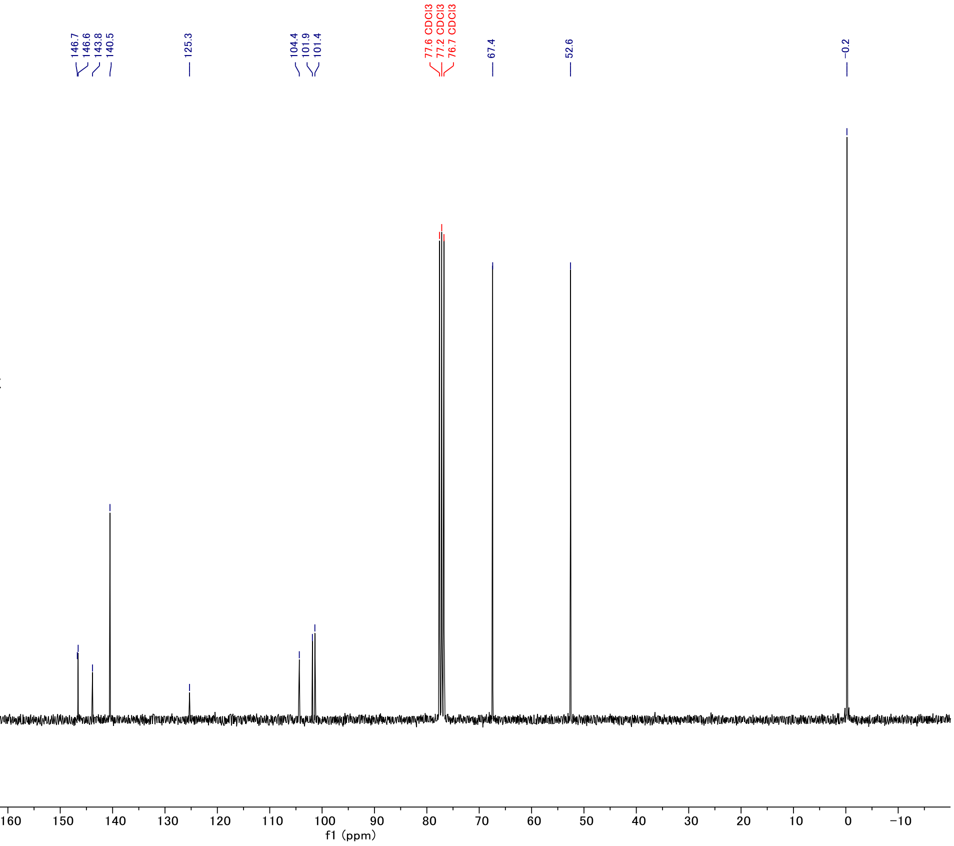

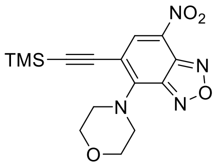

S-5

^{13}C NMR, $CDCl_3$, 75 MHz

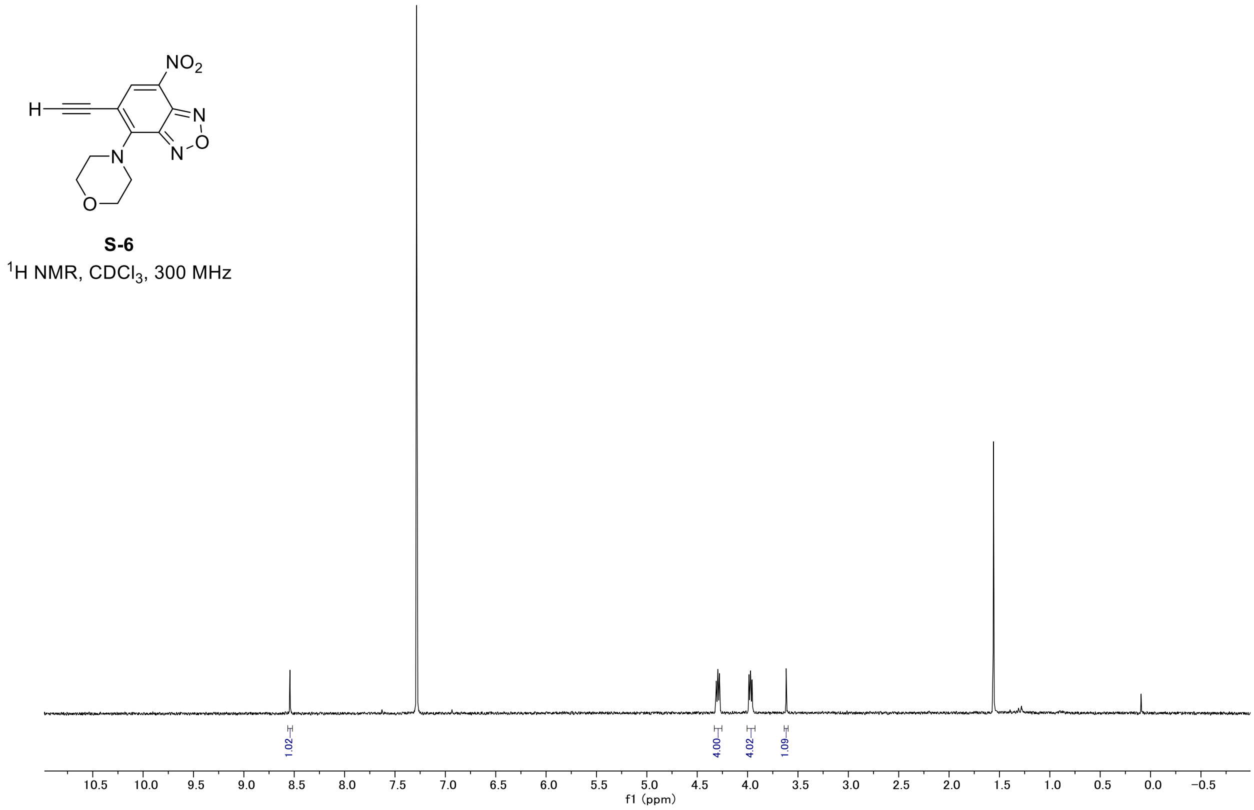
NO2
H
N
O
S-6
1H NMR, CDCl3, 300 MHz
1.02
4.00
4.02
1.09
10.5 10.0 9.5 9.0 8.5 8.0 7.5 7.0 6.5 6.0 5.5 5.0 4.5 4.0 3.5 3.0 2.5 2.0 1.5 1.0 0.5 0.0 −0.5
f1 (ppm)

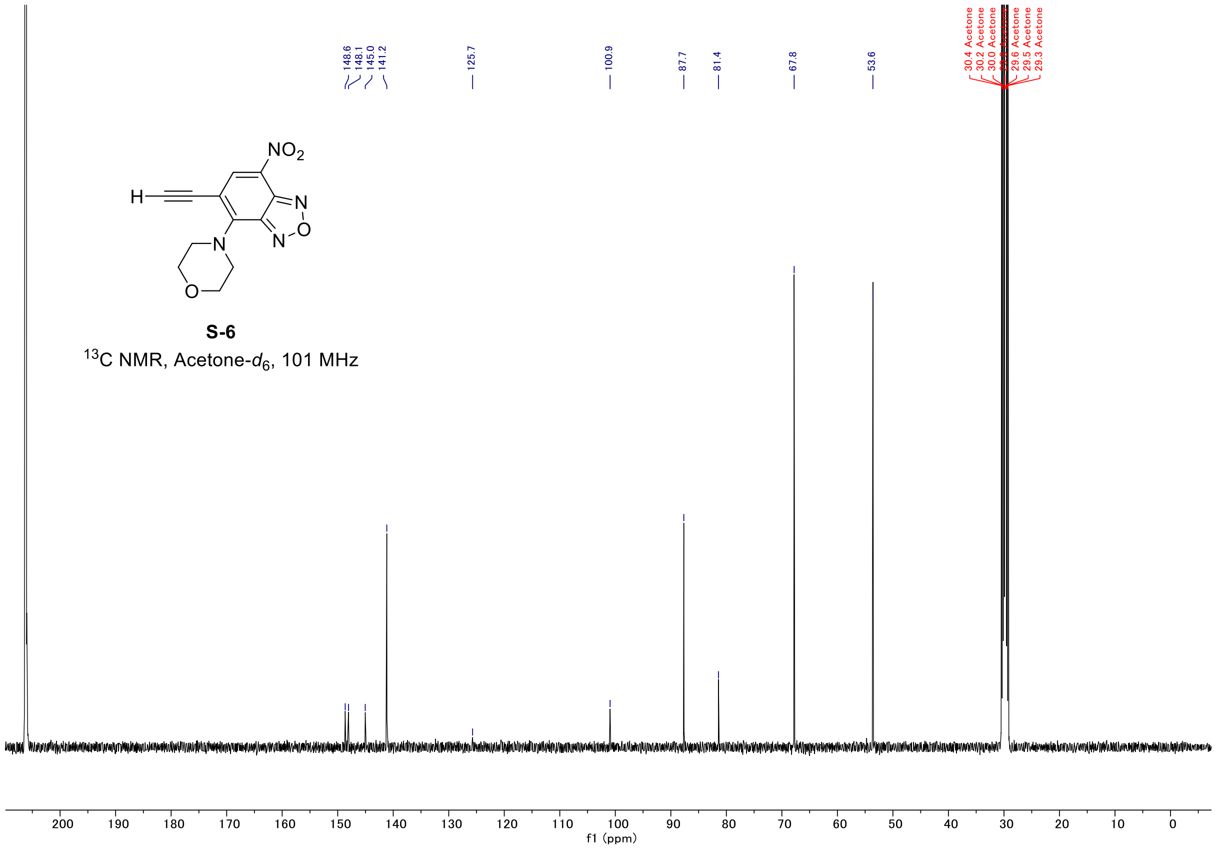
148.6
148.1
145.0
141.2
125.7
100.9
87.7
81.4
67.8
53.6
30.4 Acetone
30.2 Acetone
30.0 Acetone
29.6 Acetone
29.5 Acetone
29.3 Acetone
NO2
H
N
O
S-6
13C NMR, Acetone-d6, 101 MHz
200 190 180 170 160 150 140 130 120 110 100 90 80 70 60 50 40 30 20 10 0
f1 (ppm)

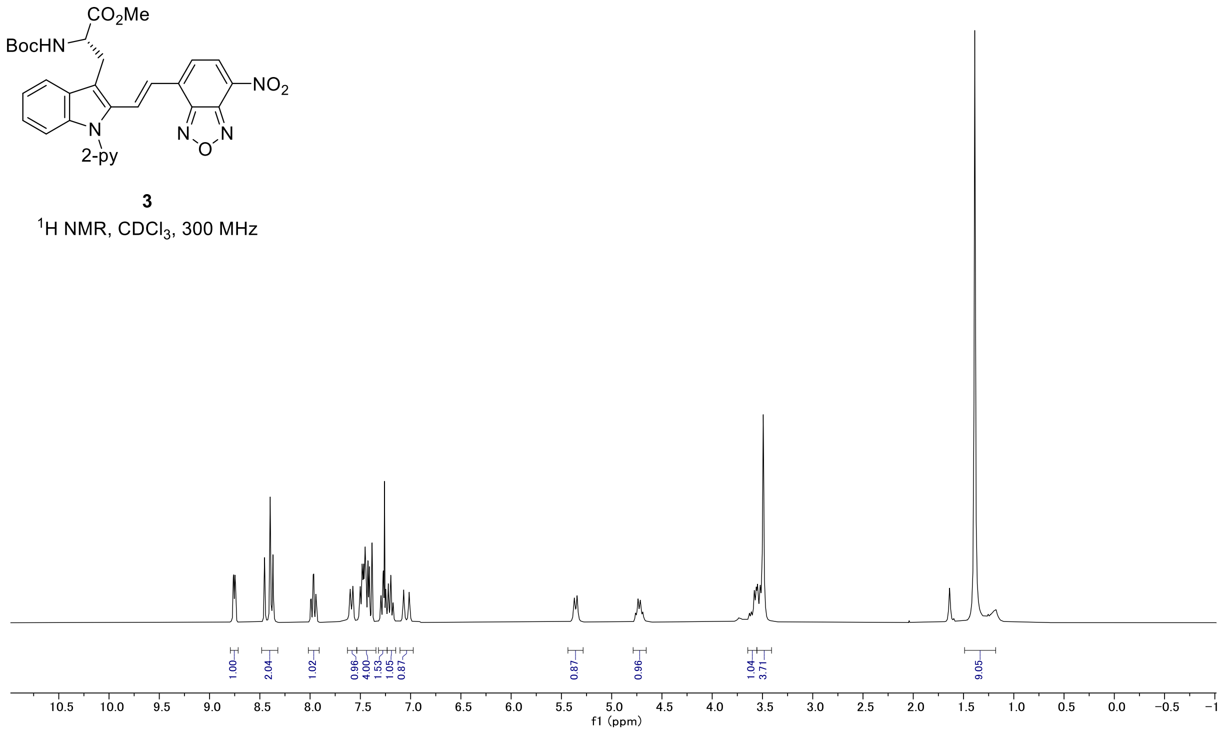

3

^{1}H NMR, $CDCl_3$, 300 MHz

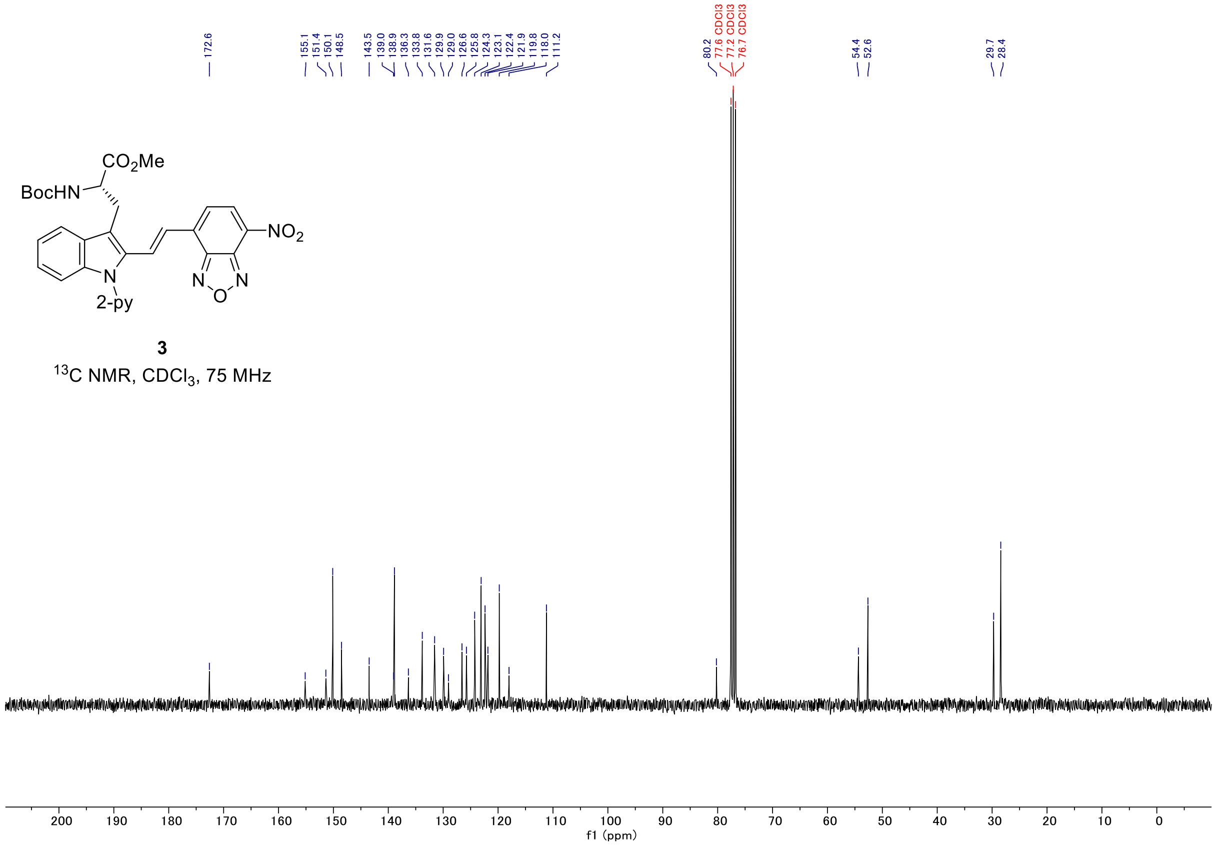

3

^{13}C NMR, $CDCl_3$, 75 MHz

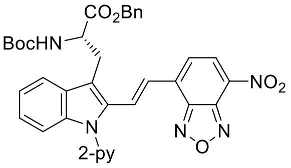

4

^{1}H NMR, $CDCl_3$, 300 MHz

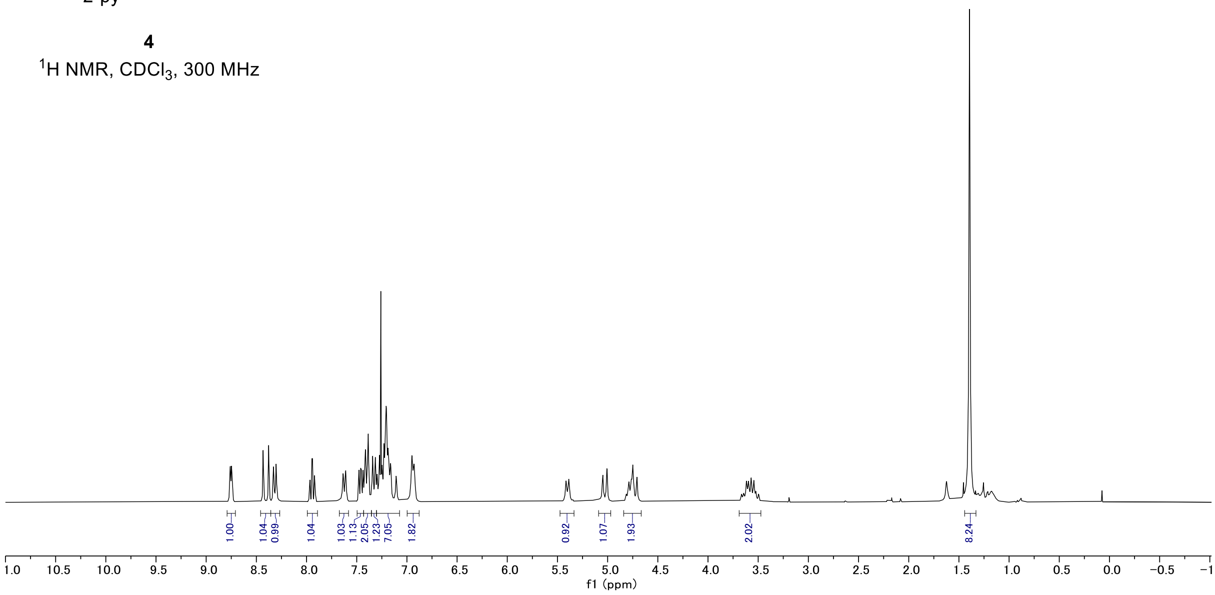

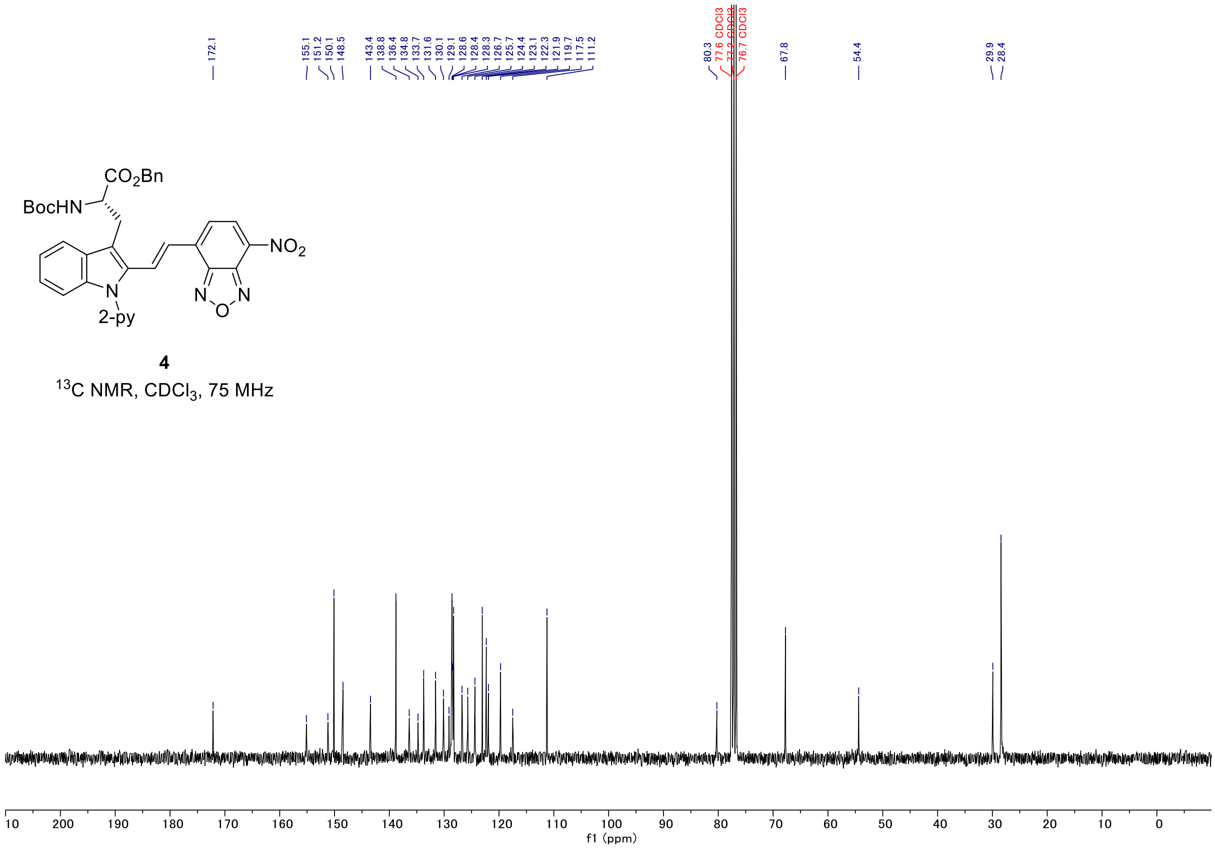

4

^{13}C NMR, $CDCl_3$, 75 MHz

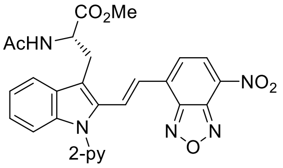

5

^{1}H NMR, $CDCl_3$, 300 MHz

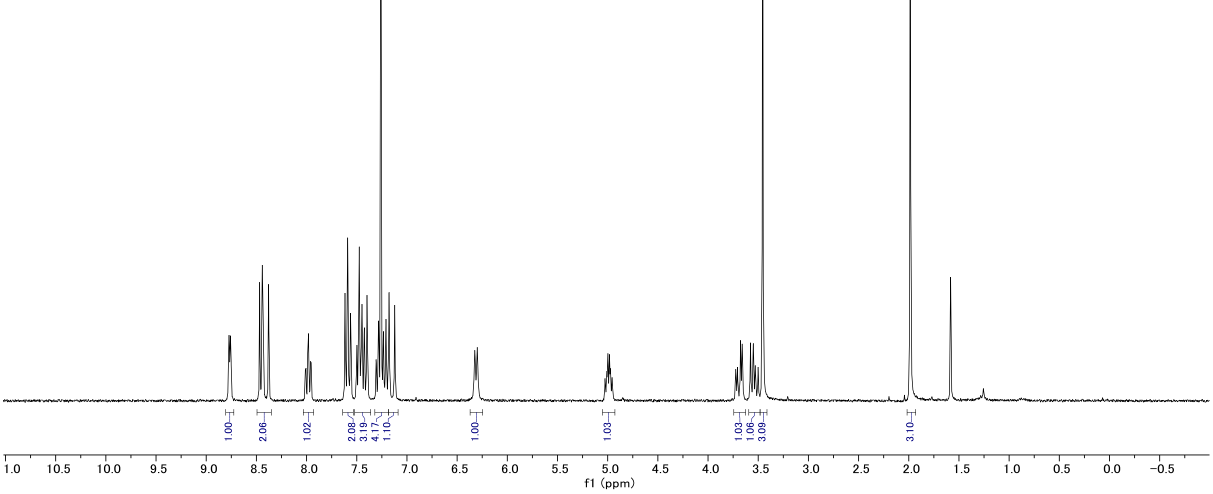

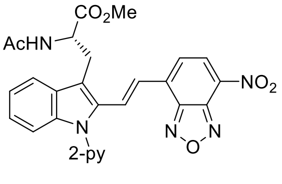

5

^{13}C NMR, $CDCl_3$, 75 MHz

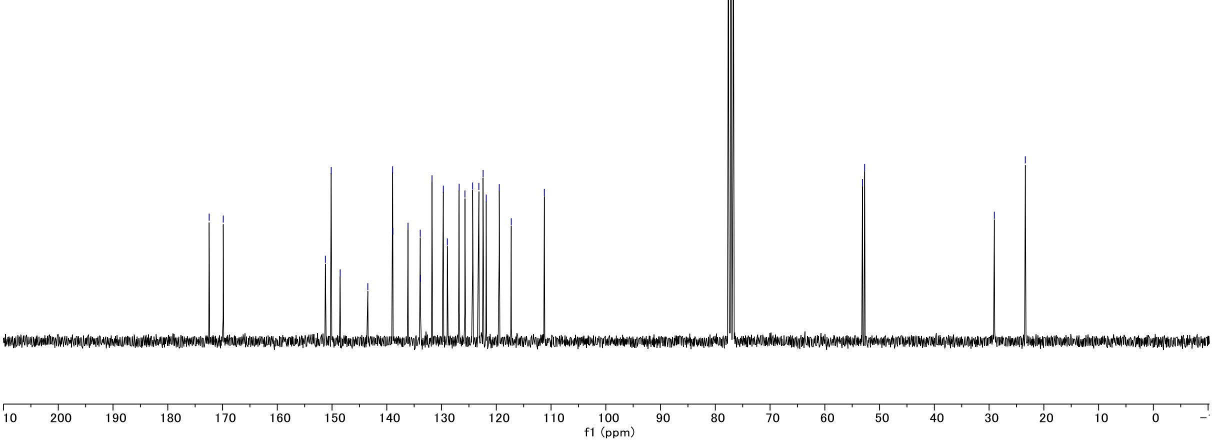

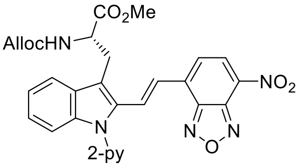

6
^1H NMR, $CDCl_3$, 300 MHz

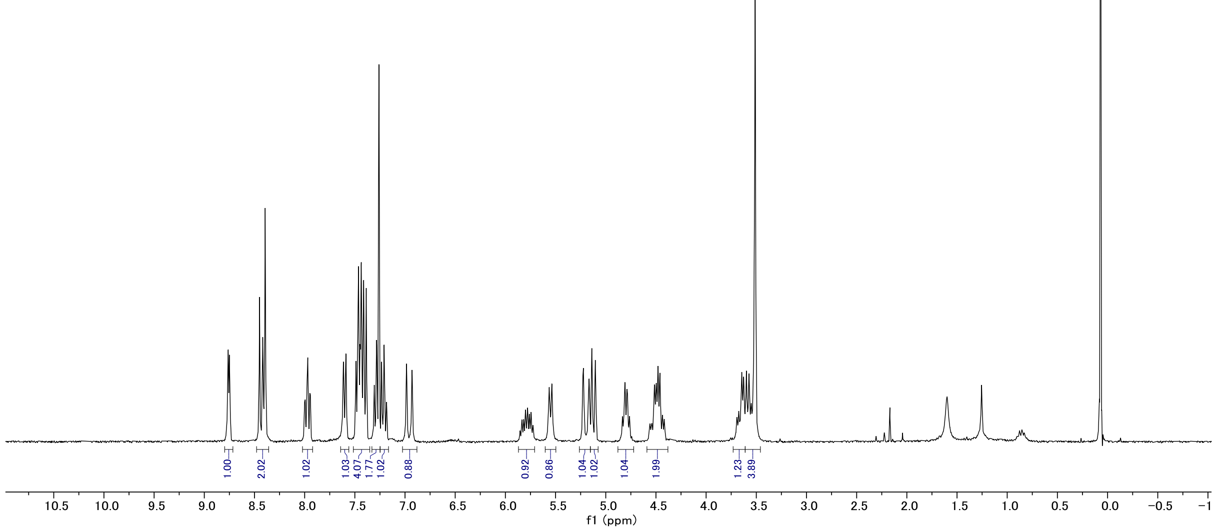

6
^{13}C NMR, $CDCl_3$, 75 MHz

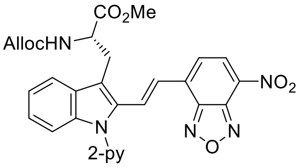

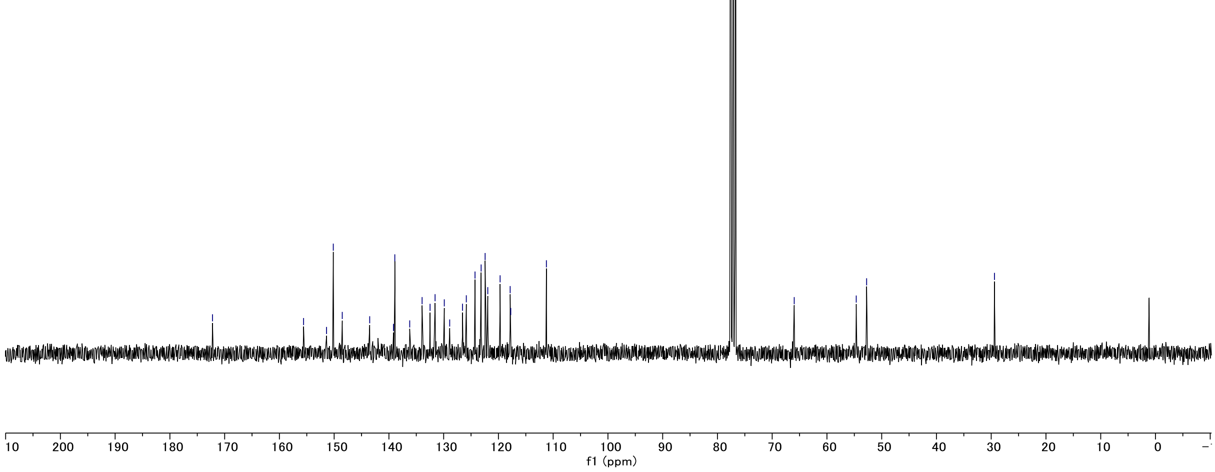

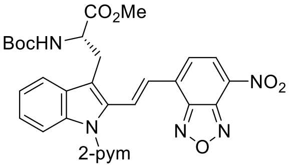

7

^{1}H NMR, $CDCl_3$, 300 MHz

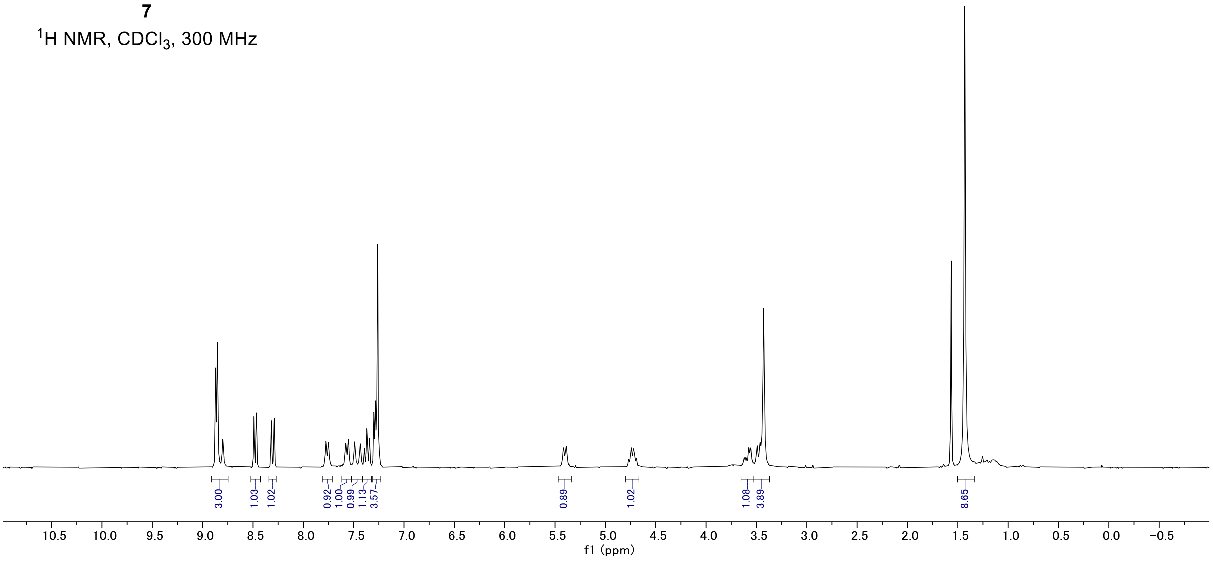

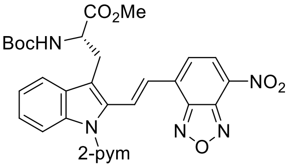

7

^{13}C NMR, $CDCl_3$, 75 MHz

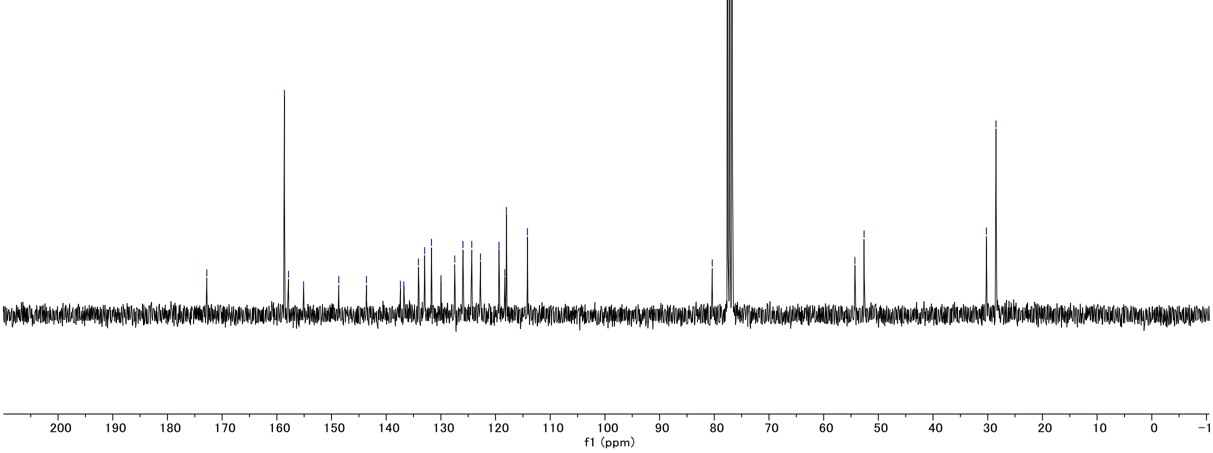

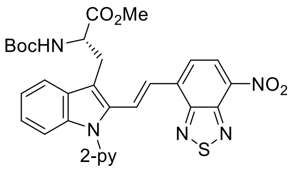

8

^{1}H NMR, $CDCl_3$, 300 MHz

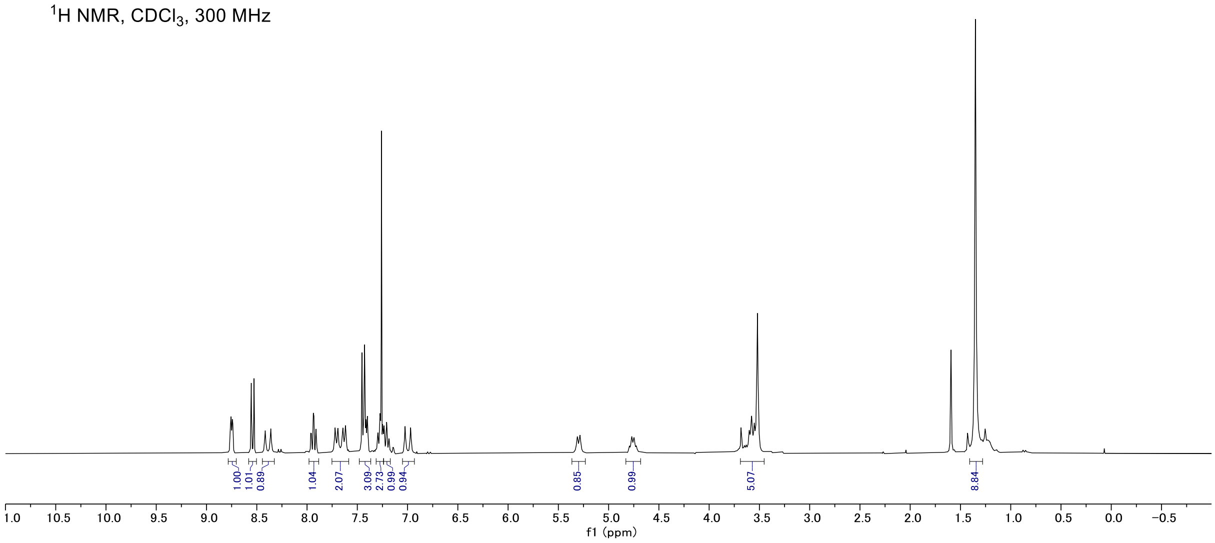

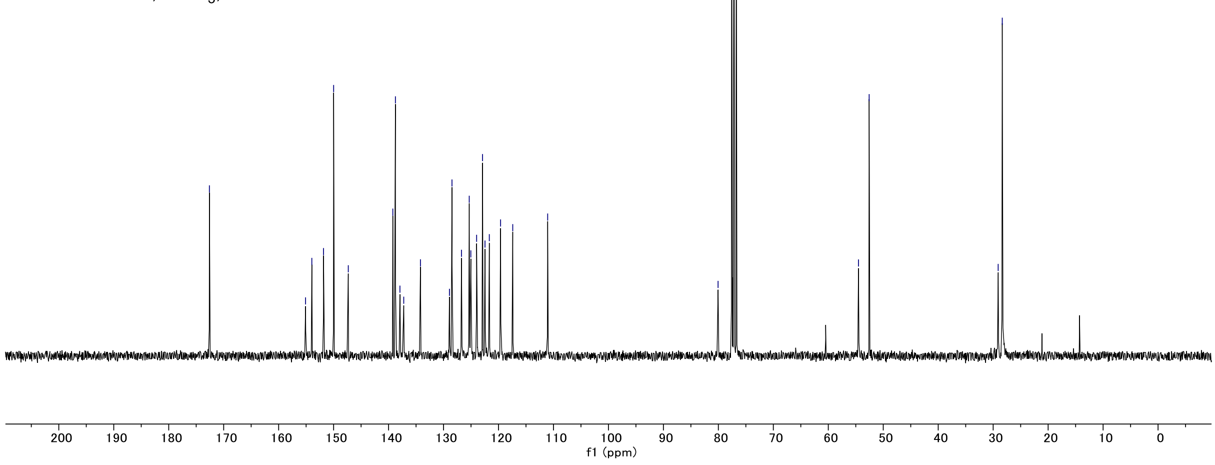

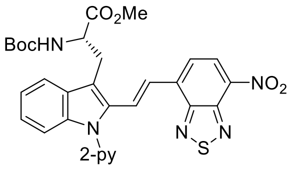

8

^{13}C NMR, $CDCl_3$, 75 MHz

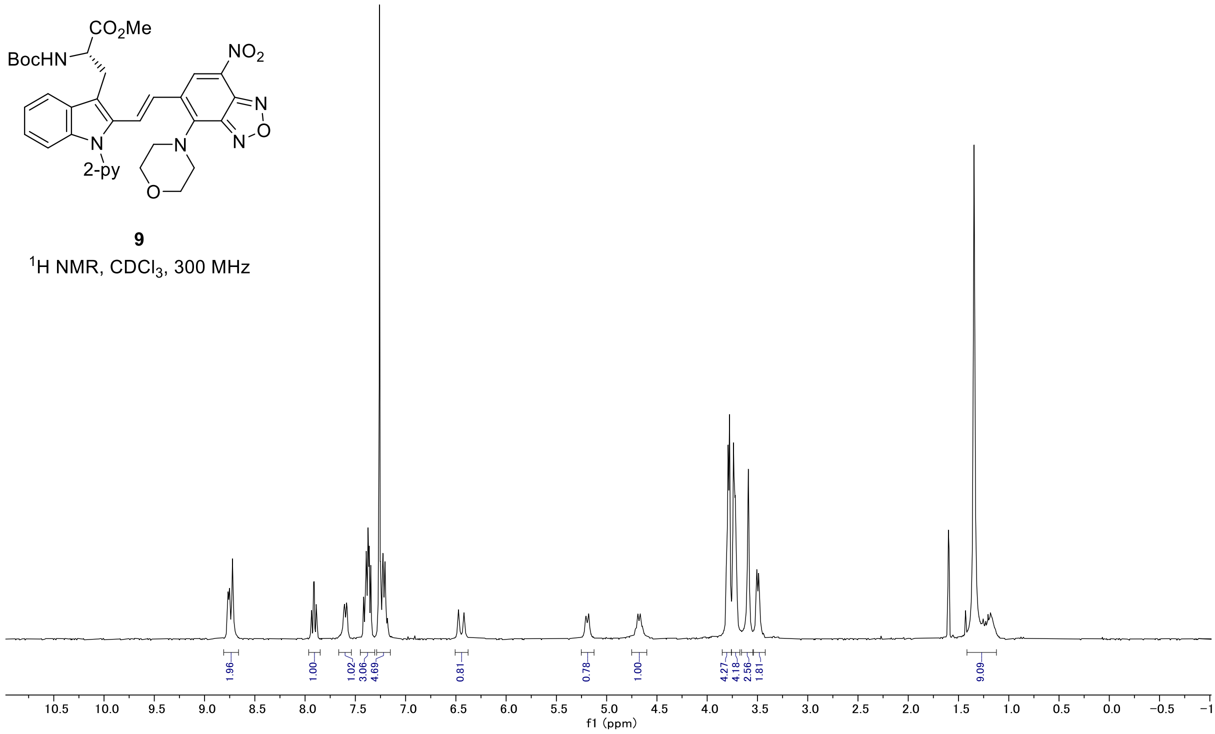
CO2Me
BocHN
NO2
N
O
N
N
2-py
N
O
9
1H NMR, CDCl3, 300 MHz
1.96
1.00
1.02
3.06
4.69
0.81
0.78
1.00
4.27
4.18
2.56
1.81
9.09
f1 (ppm)

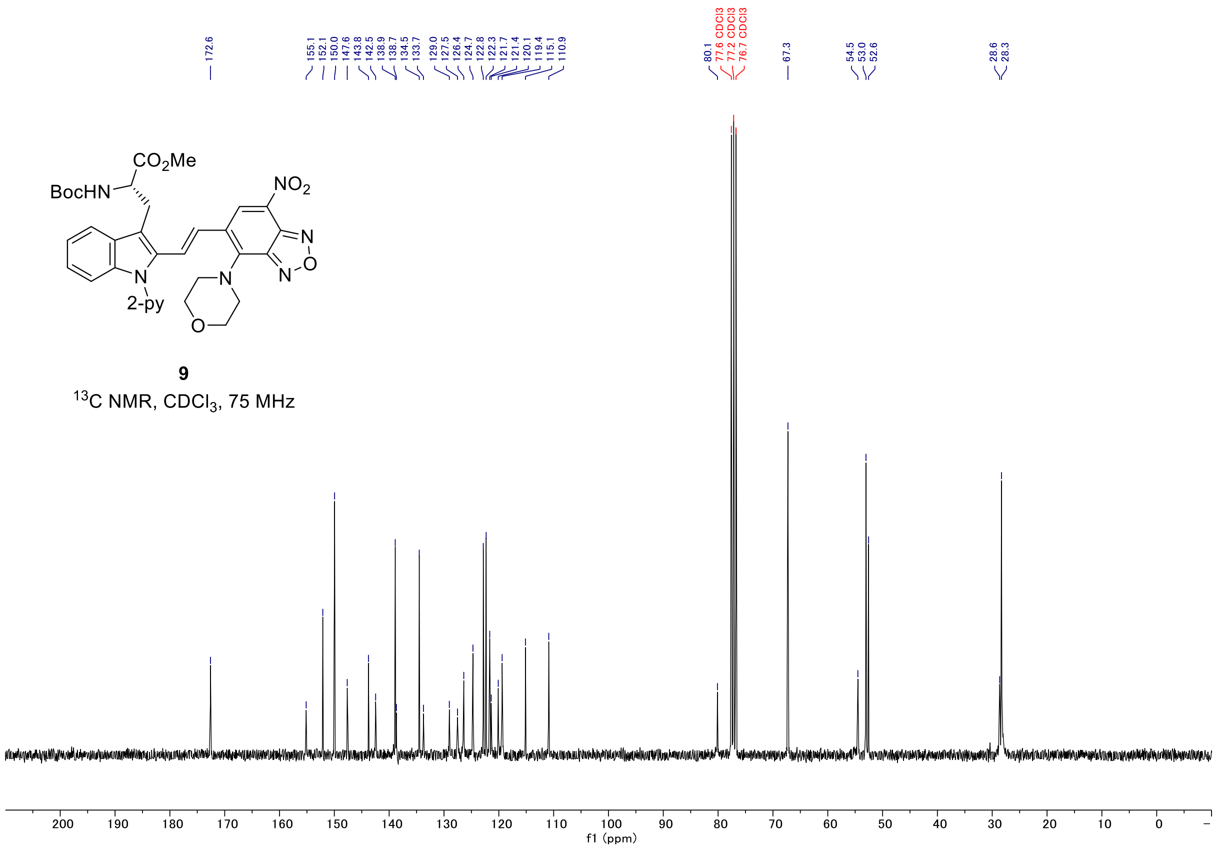
172.6
155.1
152.1
150.0
147.6
143.8
142.5
138.9
138.7
134.5
133.7
129.0
127.5
126.4
124.7
122.8
122.3
121.7
121.4
120.1
119.4
115.1
110.9
80.1
77.6 CDCl3
77.2 CDCl3
76.7 CDCl3
67.3
54.5
53.0
52.6
28.6
28.3
CO2Me
BocHN
NO2
N
O
N
N
2-py
N
O
9
13C NMR, CDCl3, 75 MHz
f1 (ppm)

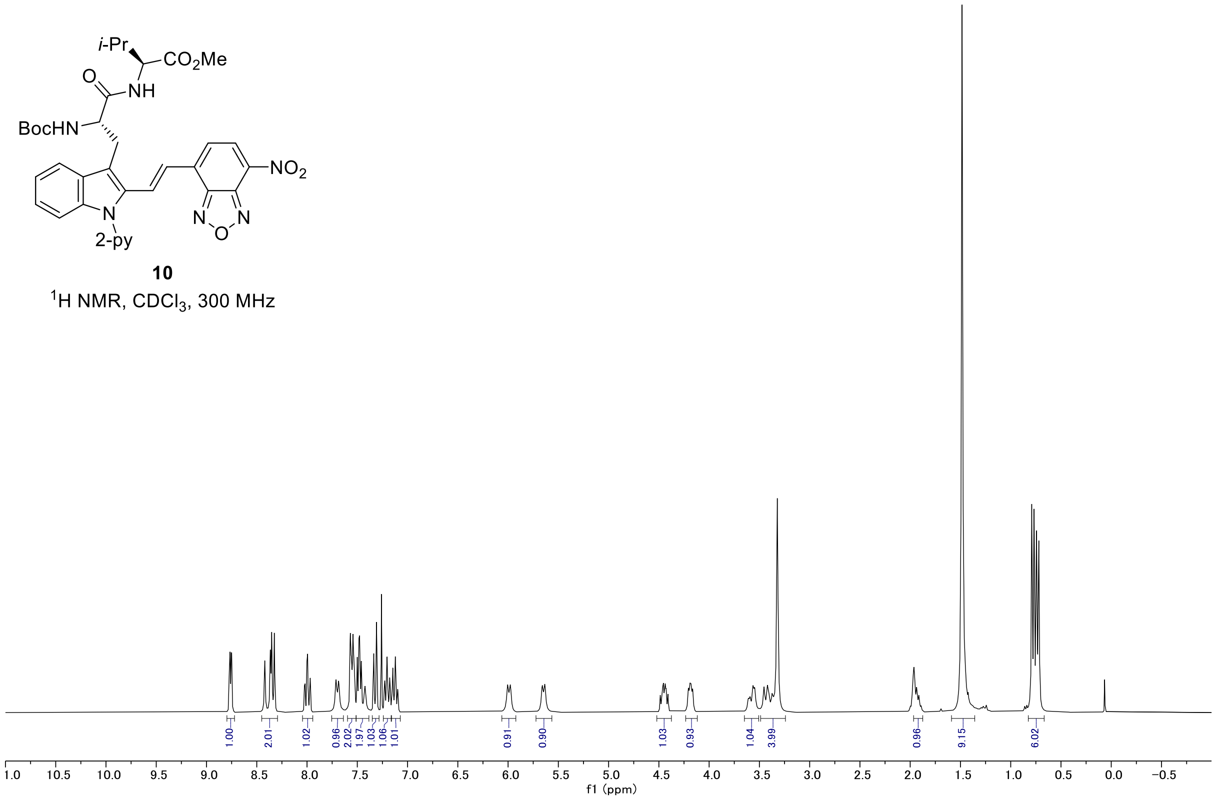

10

^{1}H NMR, $CDCl_3$, 300 MHz

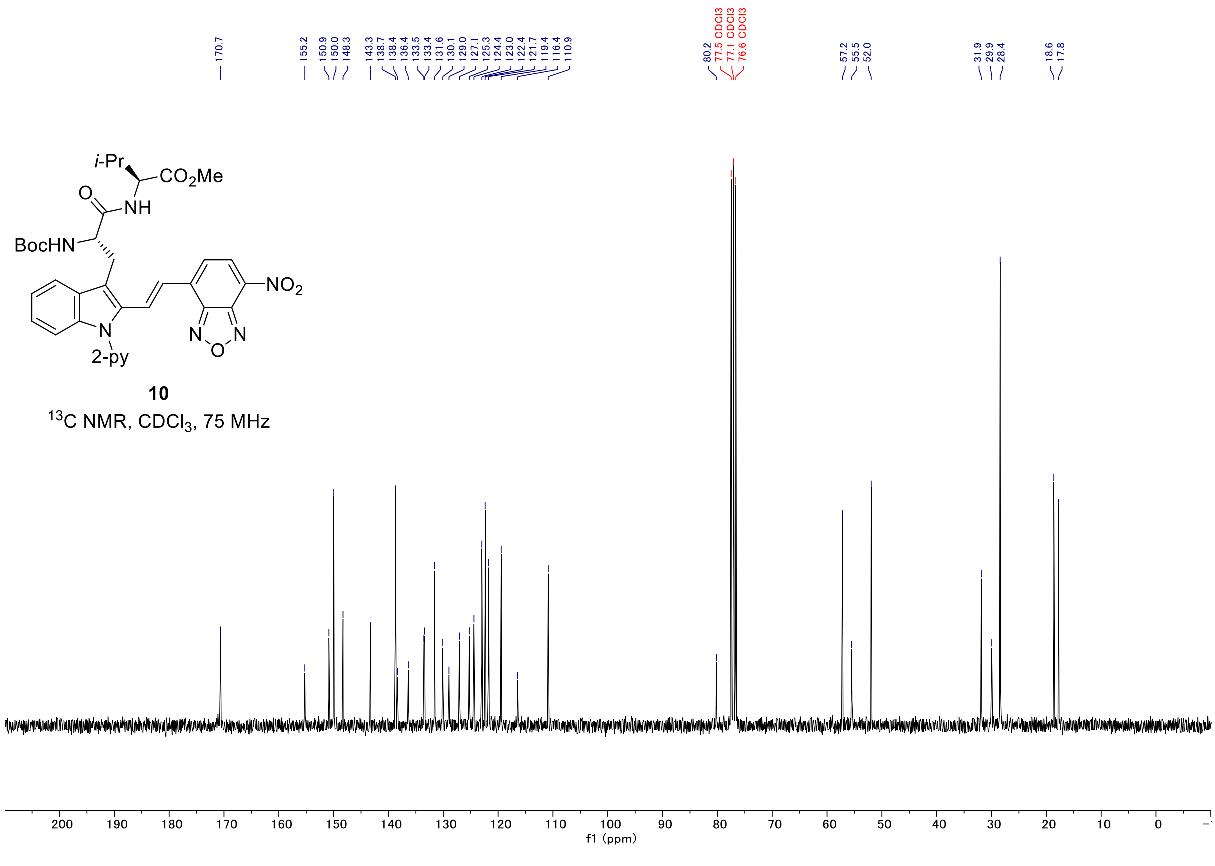

10

^{13}C NMR, $CDCl_3$, 75 MHz

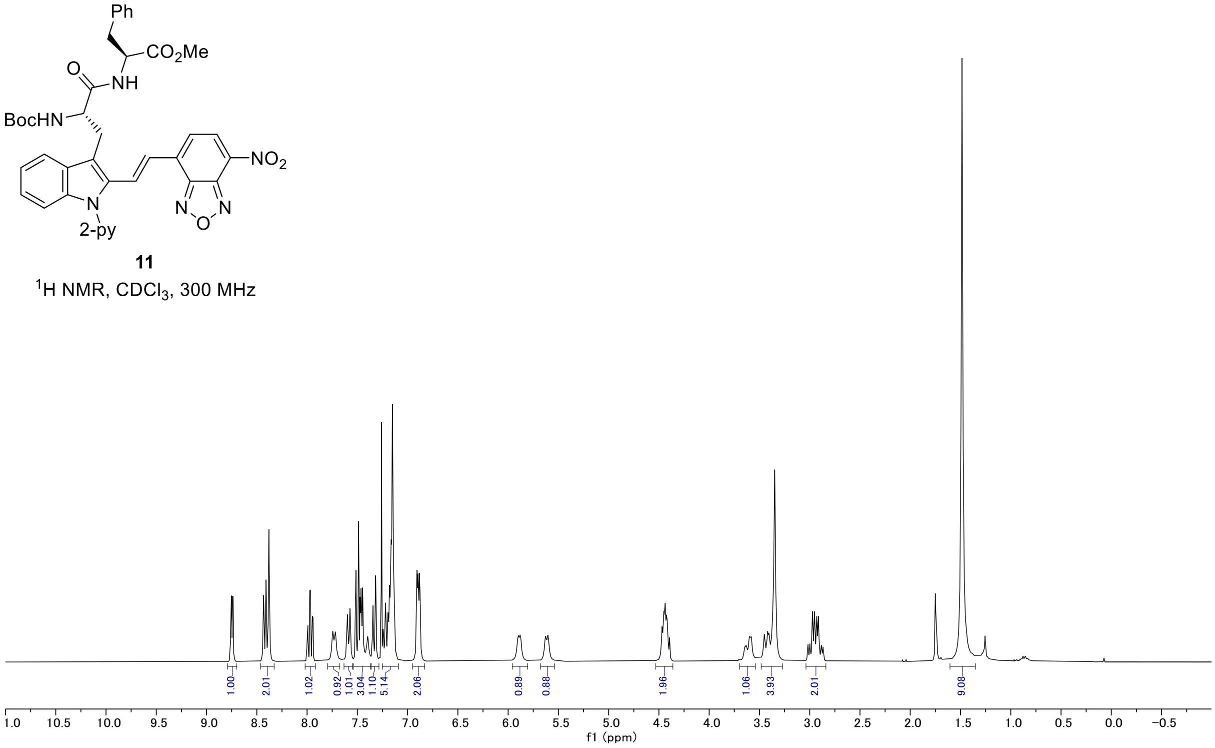

11

^{1}H NMR, $CDCl_3$, 300 MHz

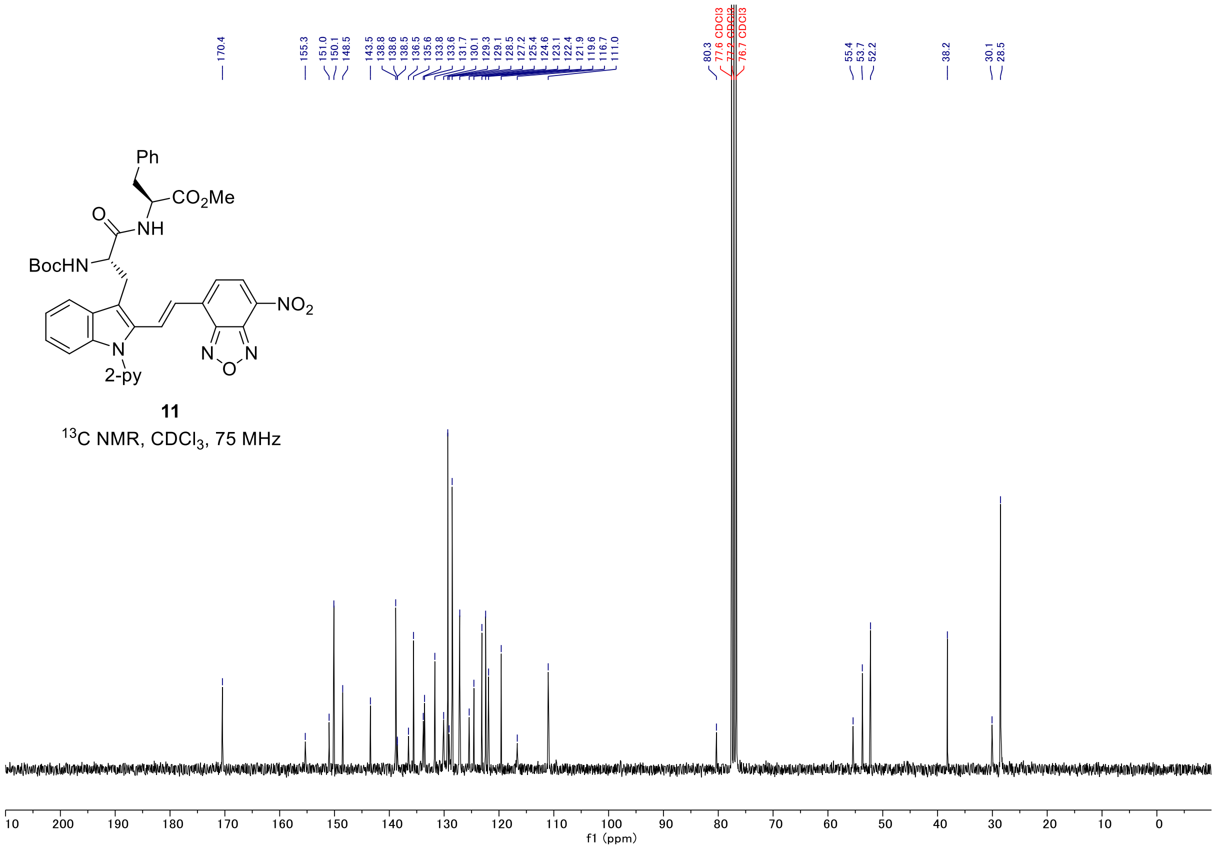

11

^{13}C NMR, $CDCl_3$, 75 MHz

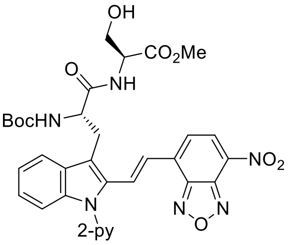

12

^{1}H NMR, $CDCl_3$, 300 MHz

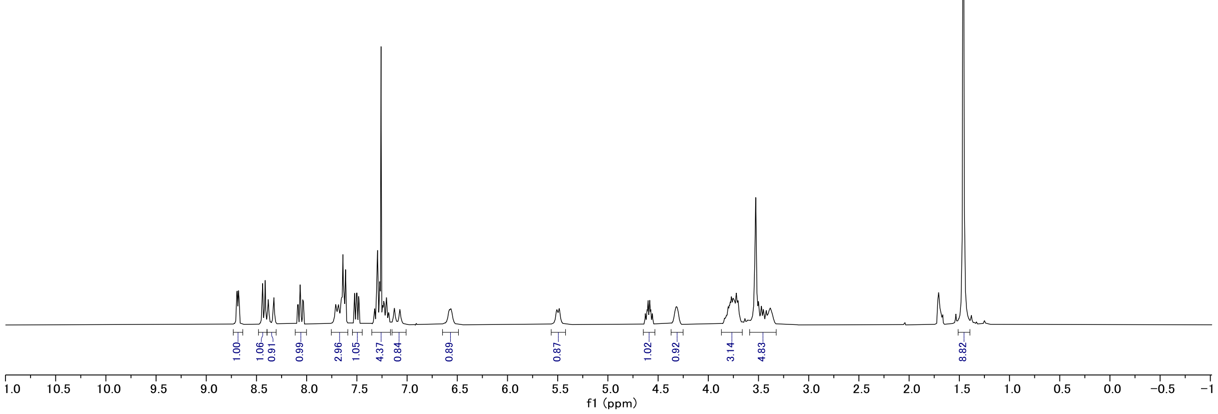

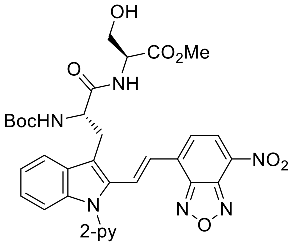

12

^{13}C NMR, $CDCl_3$, 75 MHz

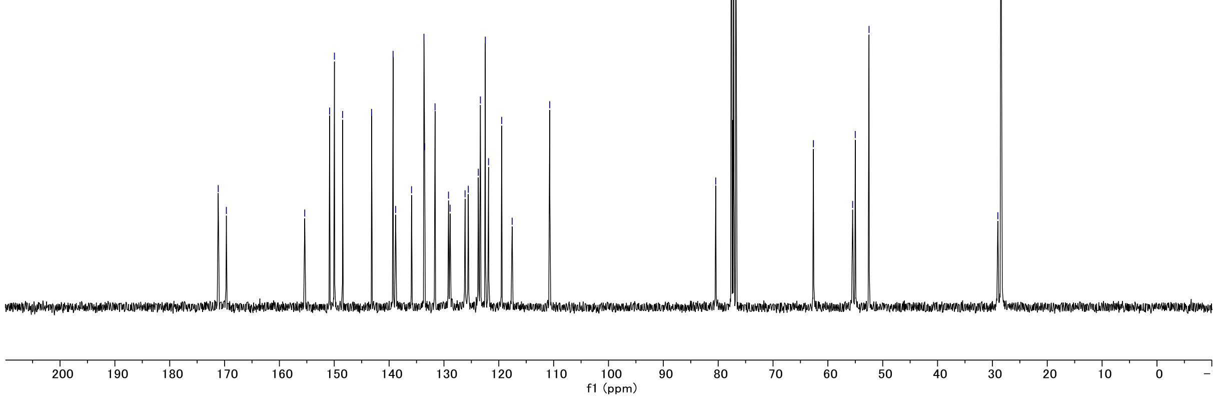

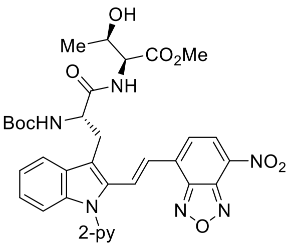

13

^{1}H NMR, $CDCl_3$, 300 MHz

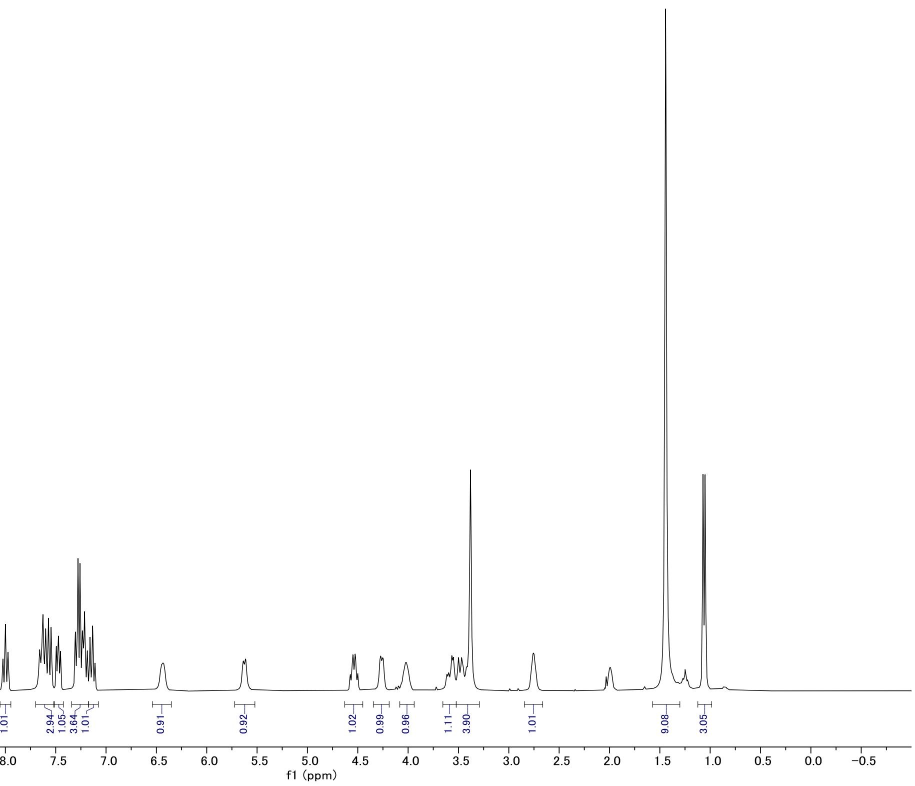

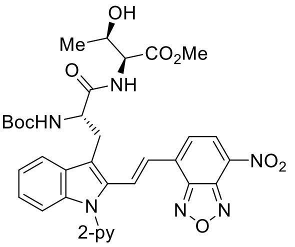

13

^{13}C NMR, $CDCl_3$, 75 MHz

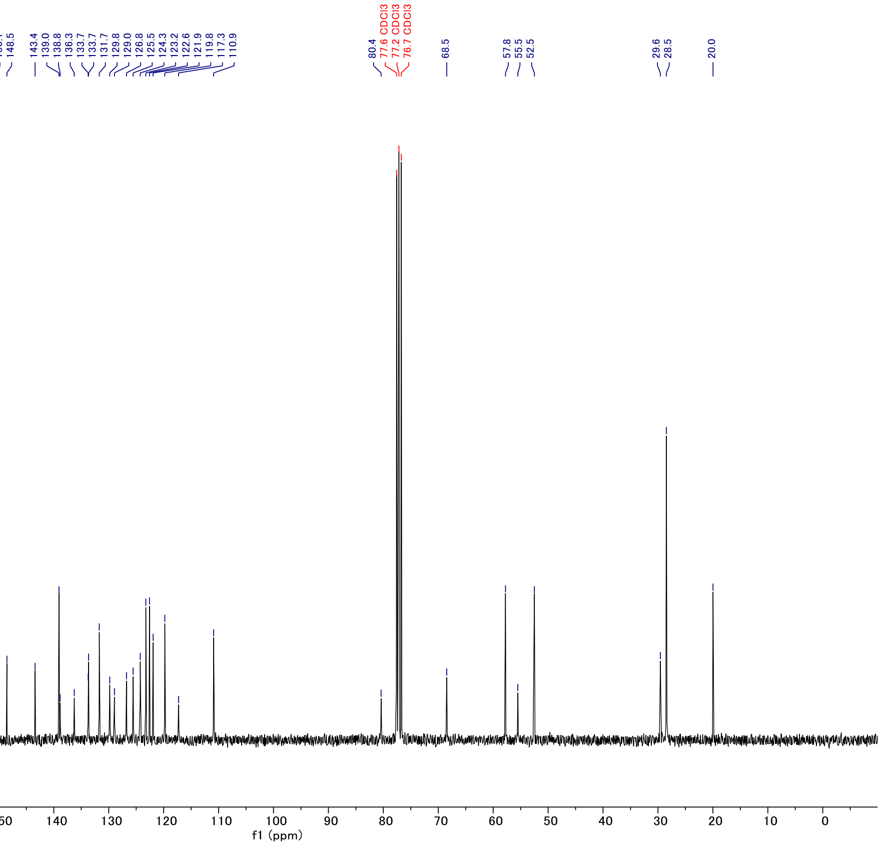

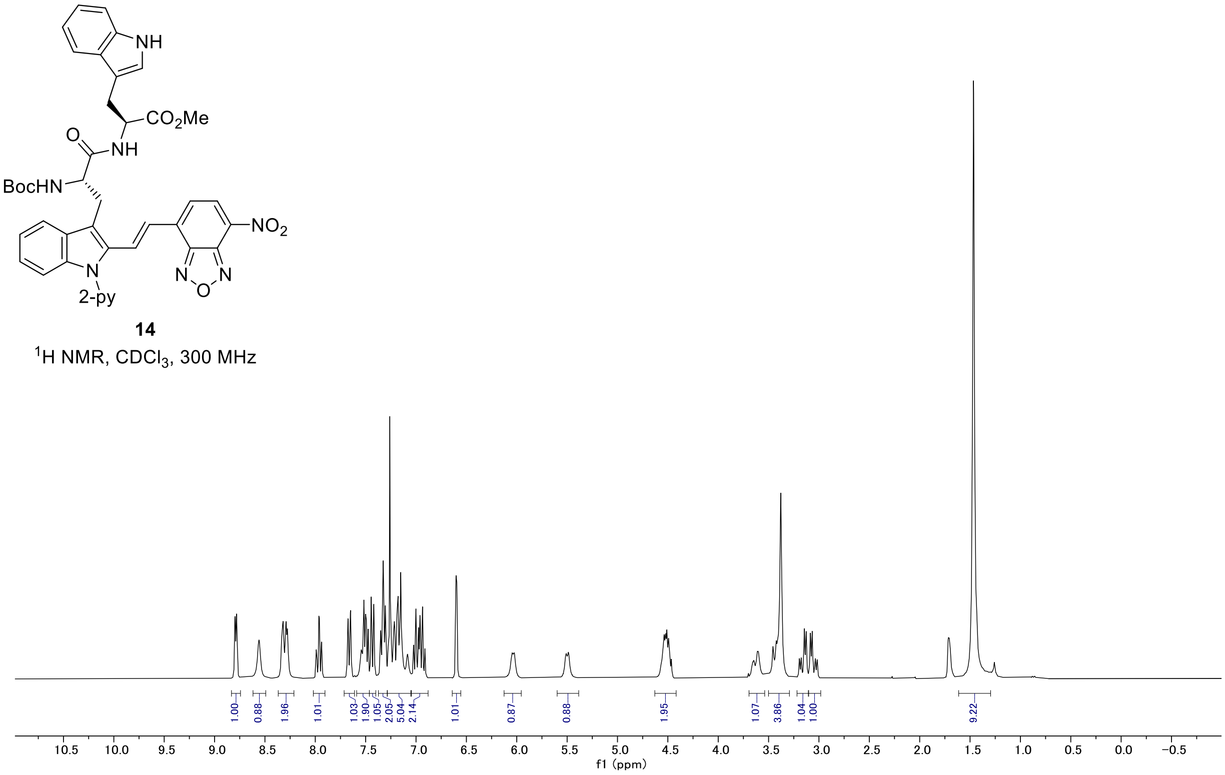

14

^{1}H NMR, $CDCl_3$, 300 MHz

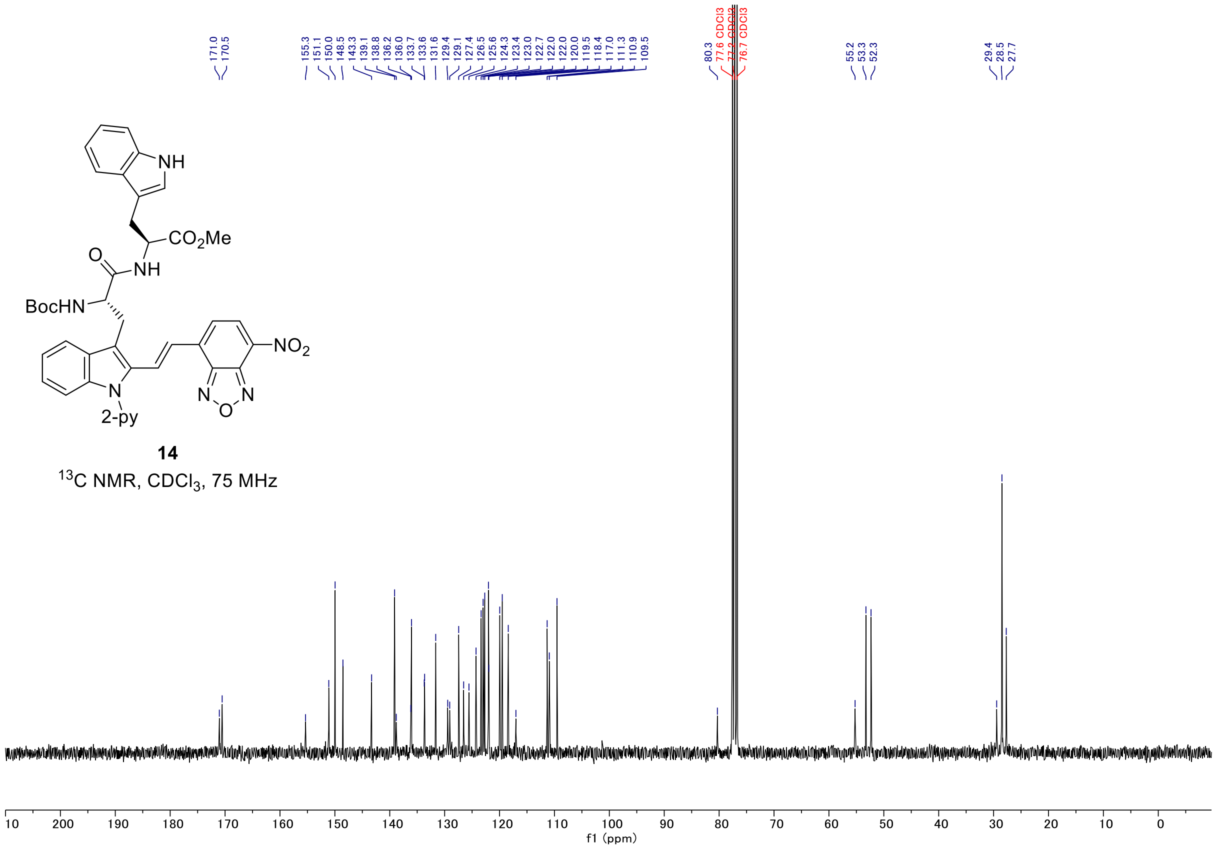

14

^{13}C NMR, $CDCl_3$, 75 MHz

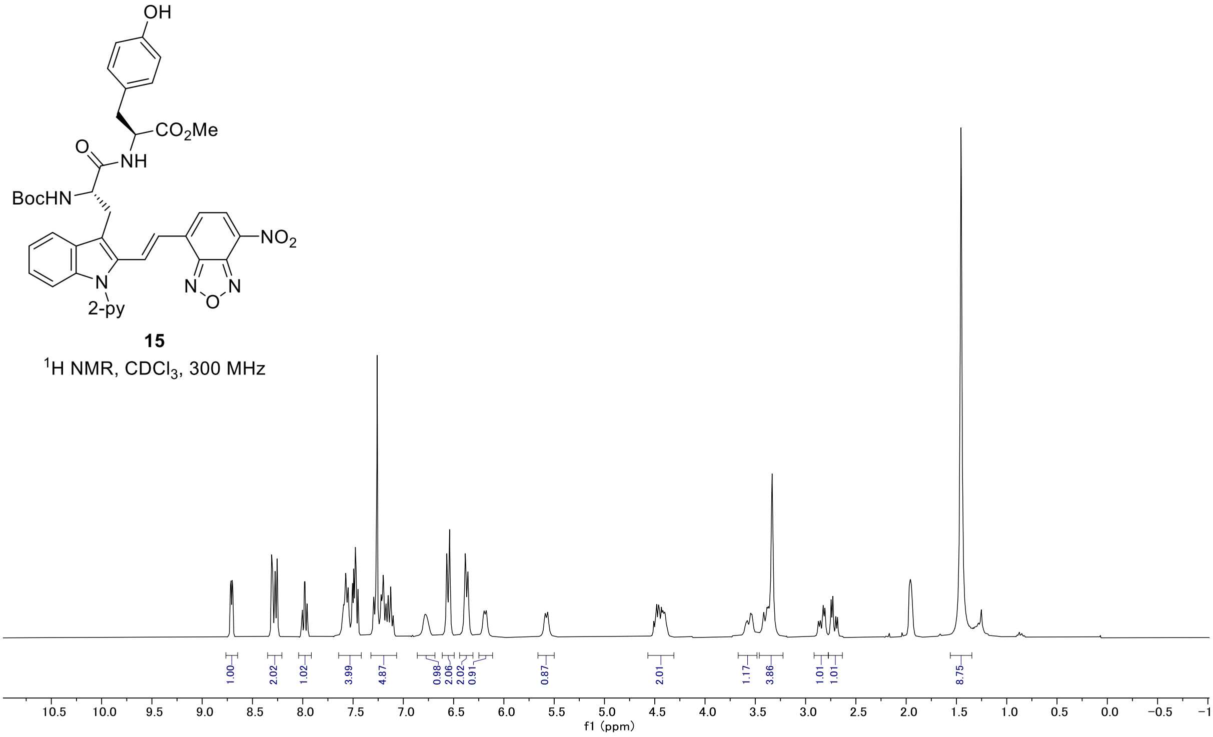

15

^{1}H NMR, $CDCl_3$, 300 MHz

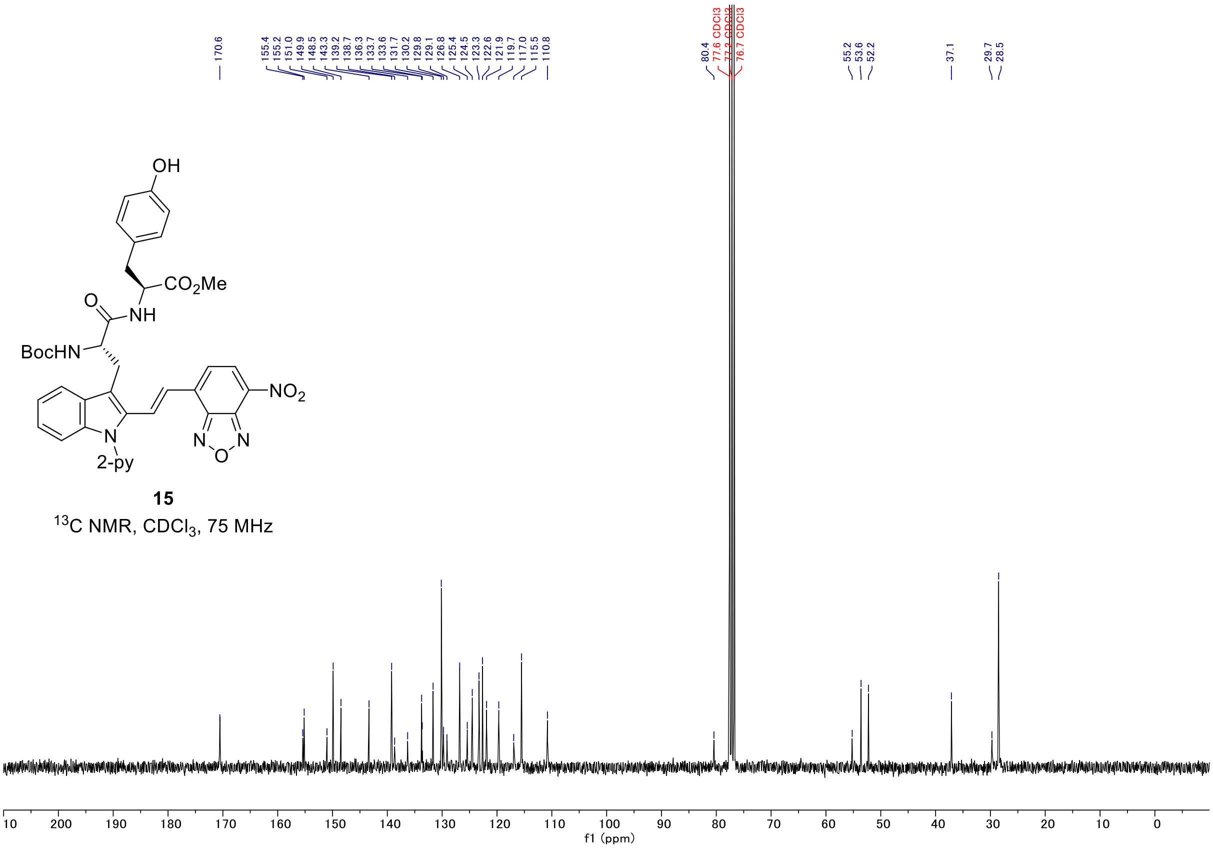

15

^{13}C NMR, $CDCl_3$, 75 MHz

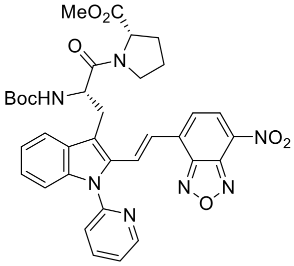

16

^{1}H NMR, $CDCl_3$, 300 MHz

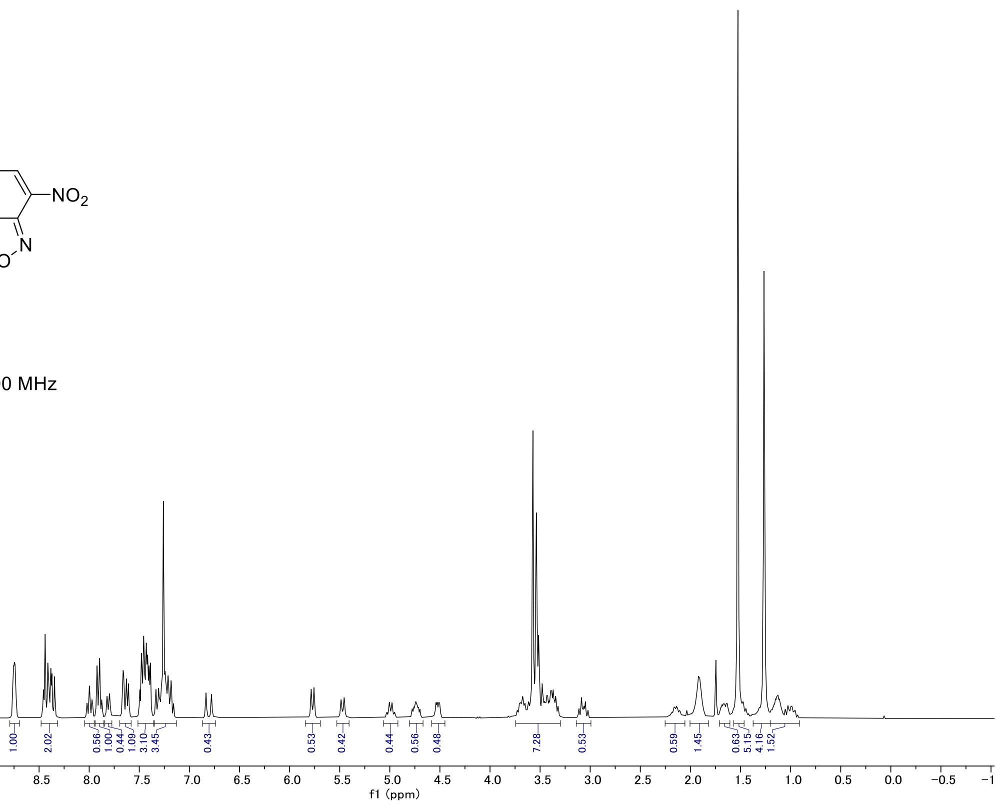

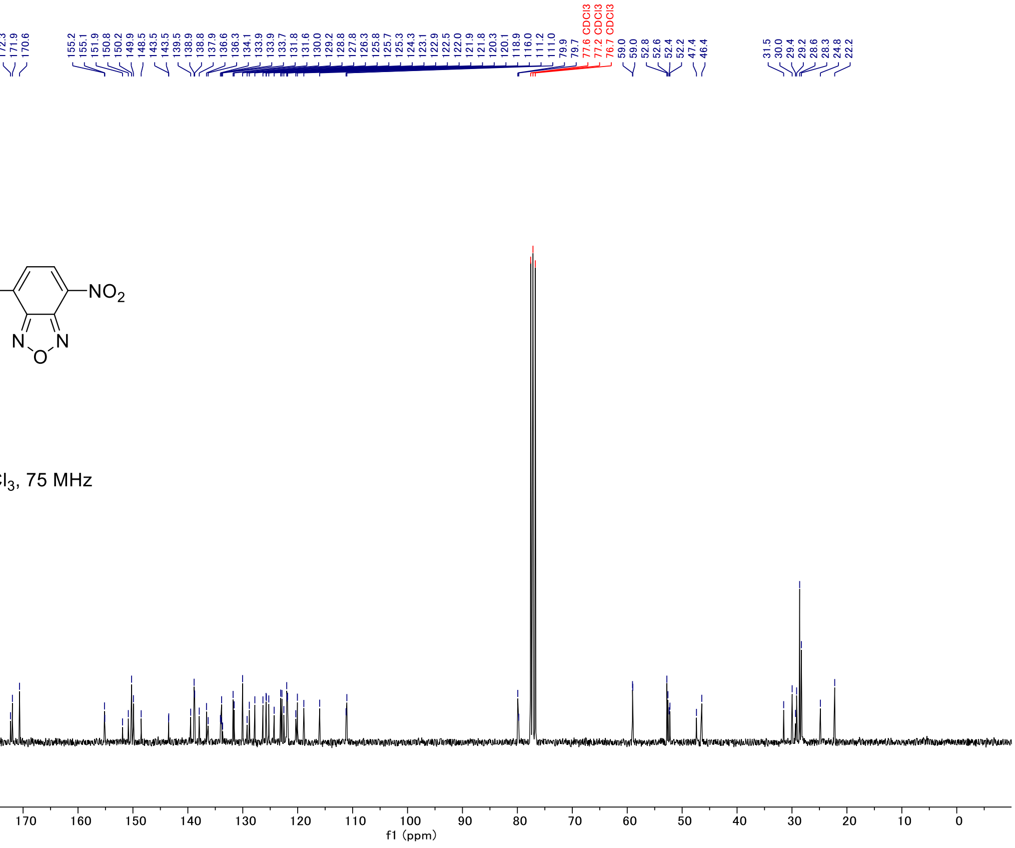

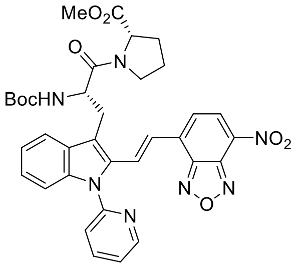

16

^{13}C NMR, $CDCl_3$, 75 MHz

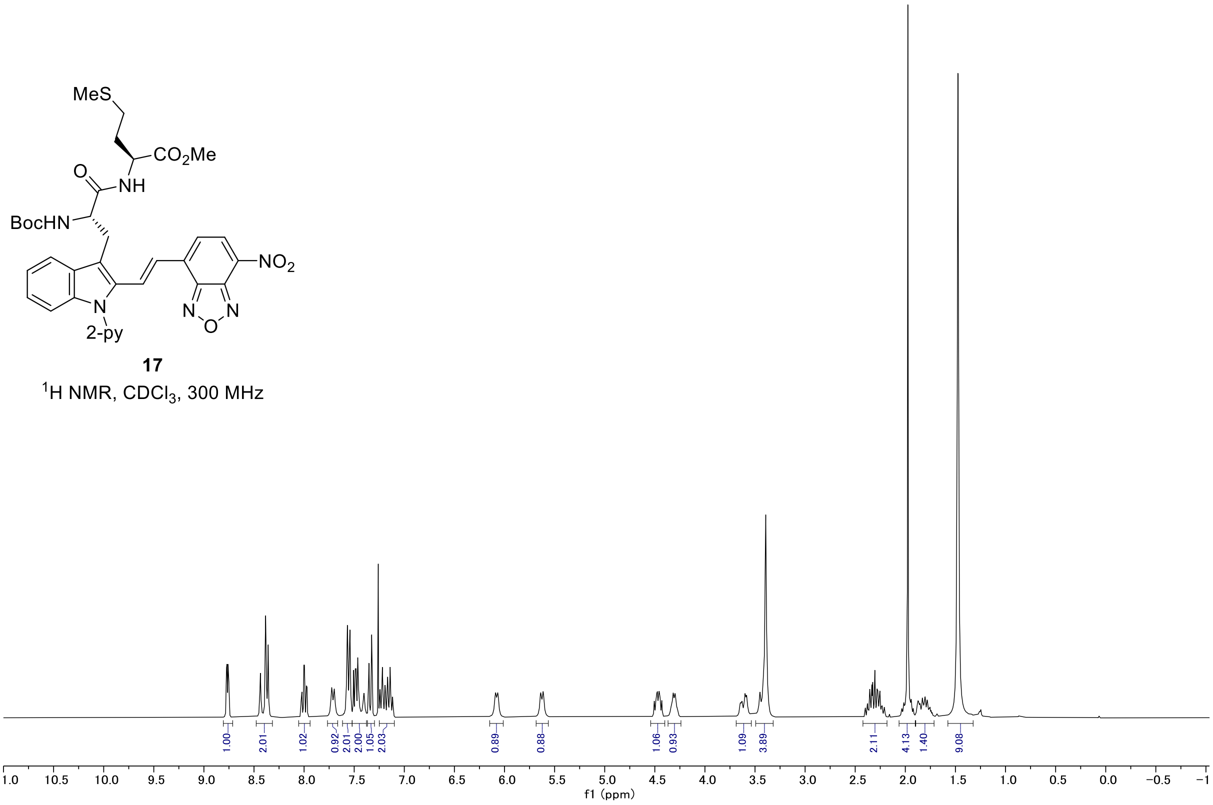

17

^{1}H NMR, $CDCl_3$, 300 MHz

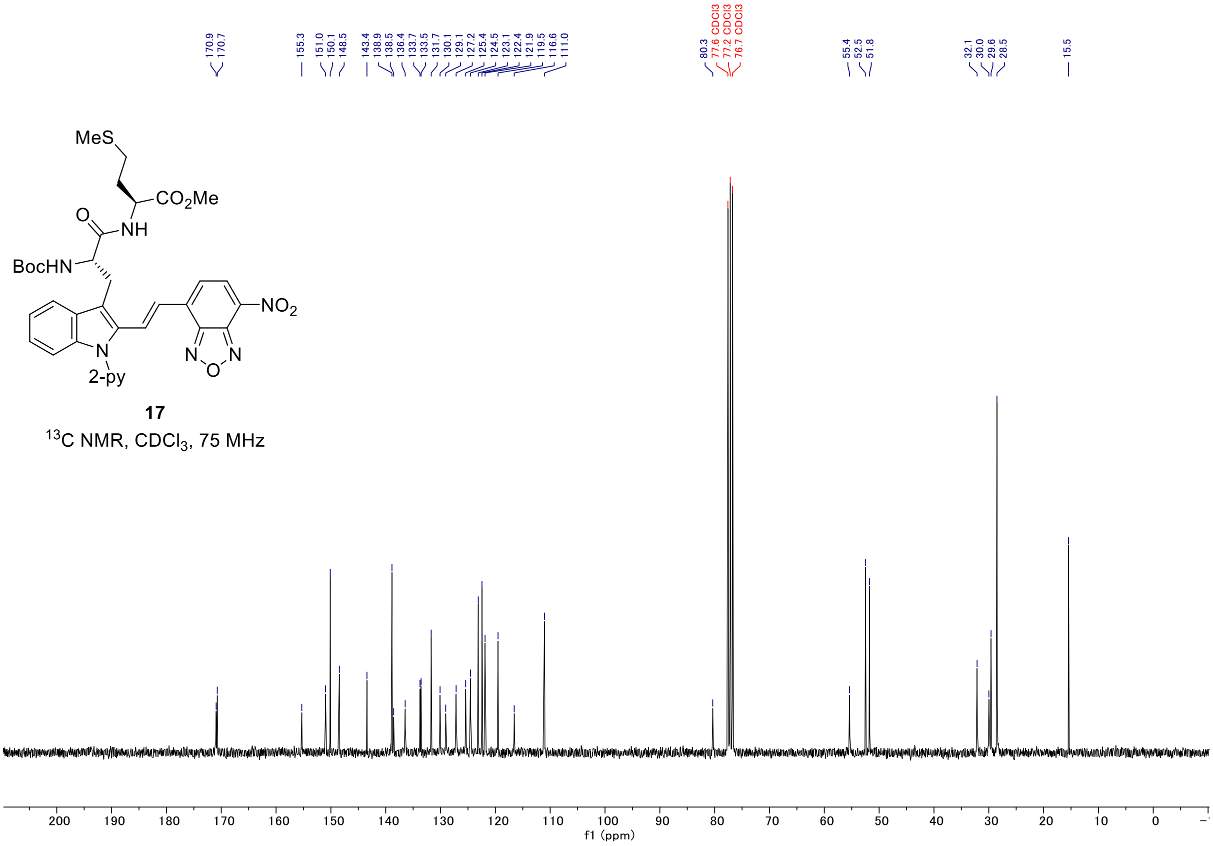

17

^{13}C NMR, $CDCl_3$, 75 MHz

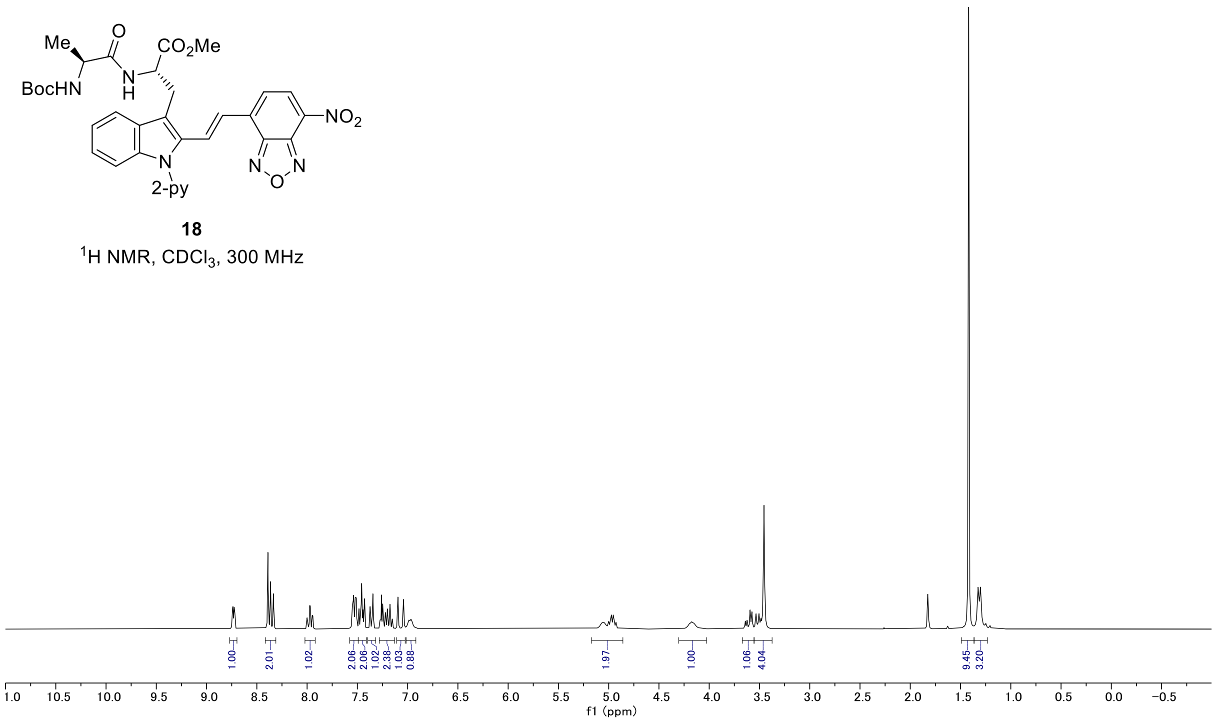

18

^{1}H NMR, $CDCl_3$, 300 MHz

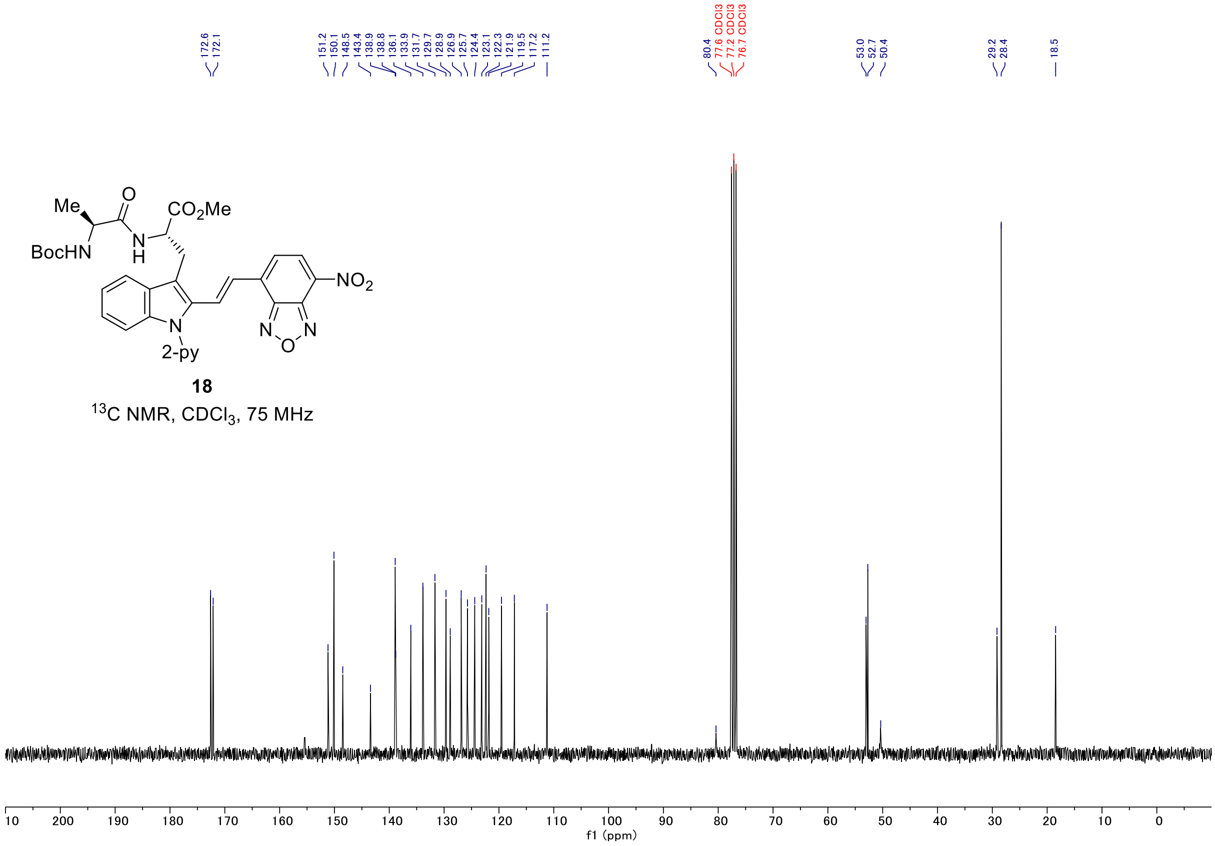

18

^{13}C NMR, $CDCl_3$, 75 MHz

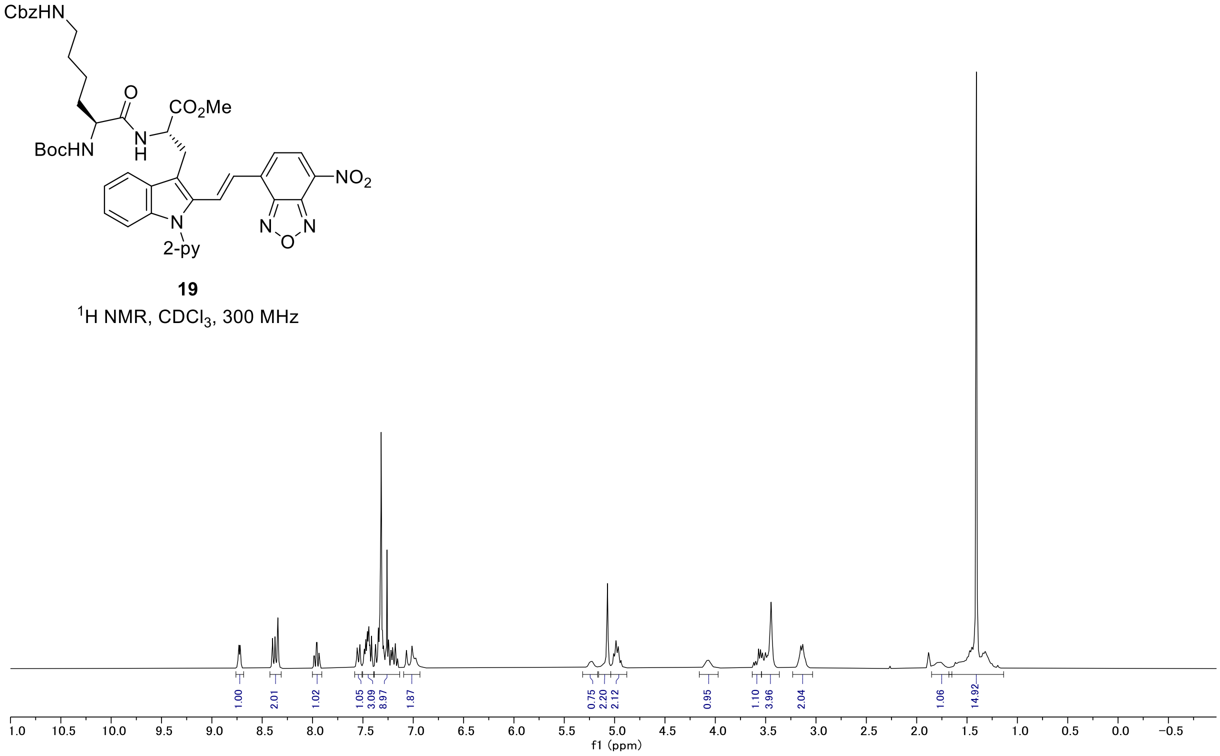

CbzHN
O
CO$_2$Me
N
H
BocHN
NO$_2$
N
2-py
N
O
N
19
^{1}H NMR, $CDCl_3$, 300 MHz
1.00
2.01
1.02
1.05
3.09
8.97
1.87
0.75
2.20
2.12
0.95
1.10
3.96
2.04
1.06
14.92
1.0 10.5 10.0 9.5 9.0 8.5 8.0 7.5 7.0 6.5 6.0 5.5 5.0 4.5 4.0 3.5 3.0 2.5 2.0 1.5 1.0 0.5 0.0 −0.5
f1 (ppm)

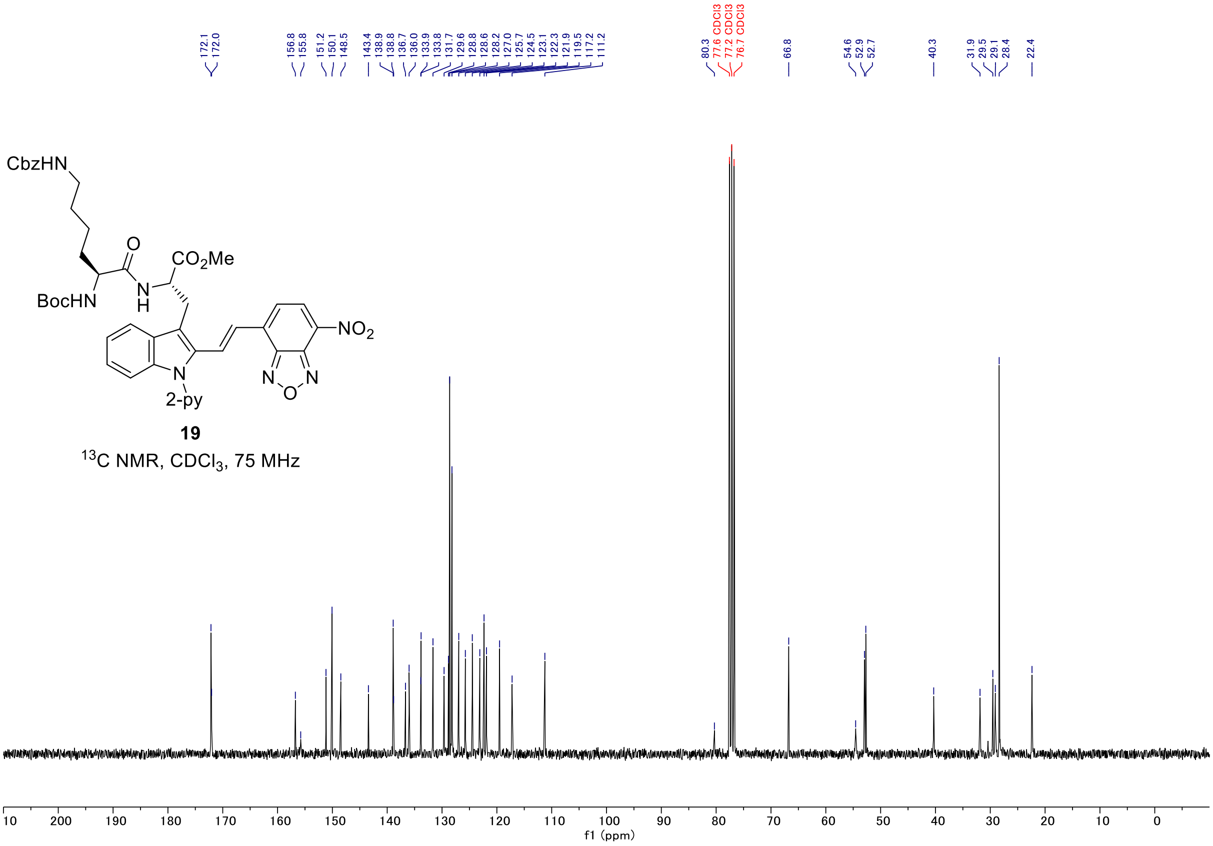

172.1
172.0
156.8
155.8
151.2
150.1
148.5
143.4
138.9
138.8
136.7
136.0
133.9
133.8
131.7
129.6
128.8
128.6
128.2
127.0
125.7
124.5
123.1
122.3
121.9
119.5
117.2
111.2
80.3
77.6 CDCl3
77.2 CDCl3
76.7 CDCl3
66.8
54.6
52.9
52.7
40.3
31.9
29.5
29.1
28.4
22.4
CbzHN
O
CO$_2$Me
N
H
BocHN
NO$_2$
N
2-py
N
O
N
19
^{13}C NMR, $CDCl_3$, 75 MHz
10 200 190 180 170 160 150 140 130 120 110 100 90 80 70 60 50 40 30 20 10 0
f1 (ppm)

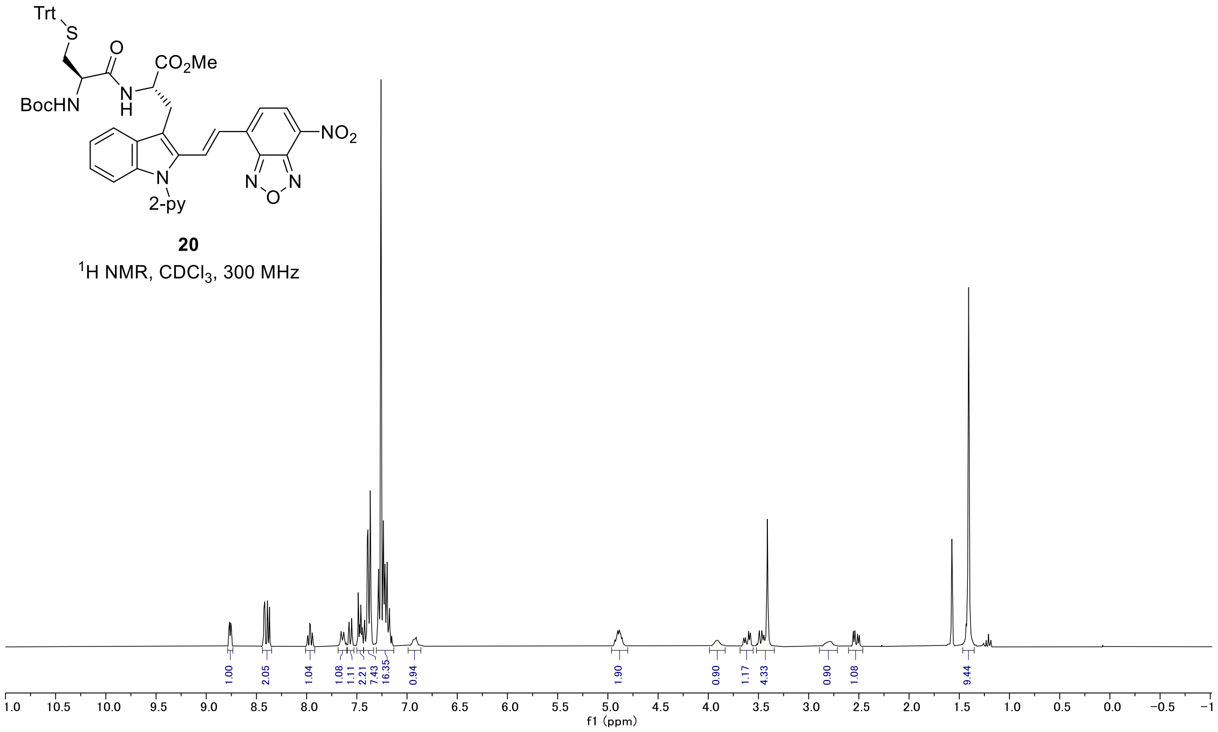

20

^{1}H NMR, $CDCl_3$, 300 MHz

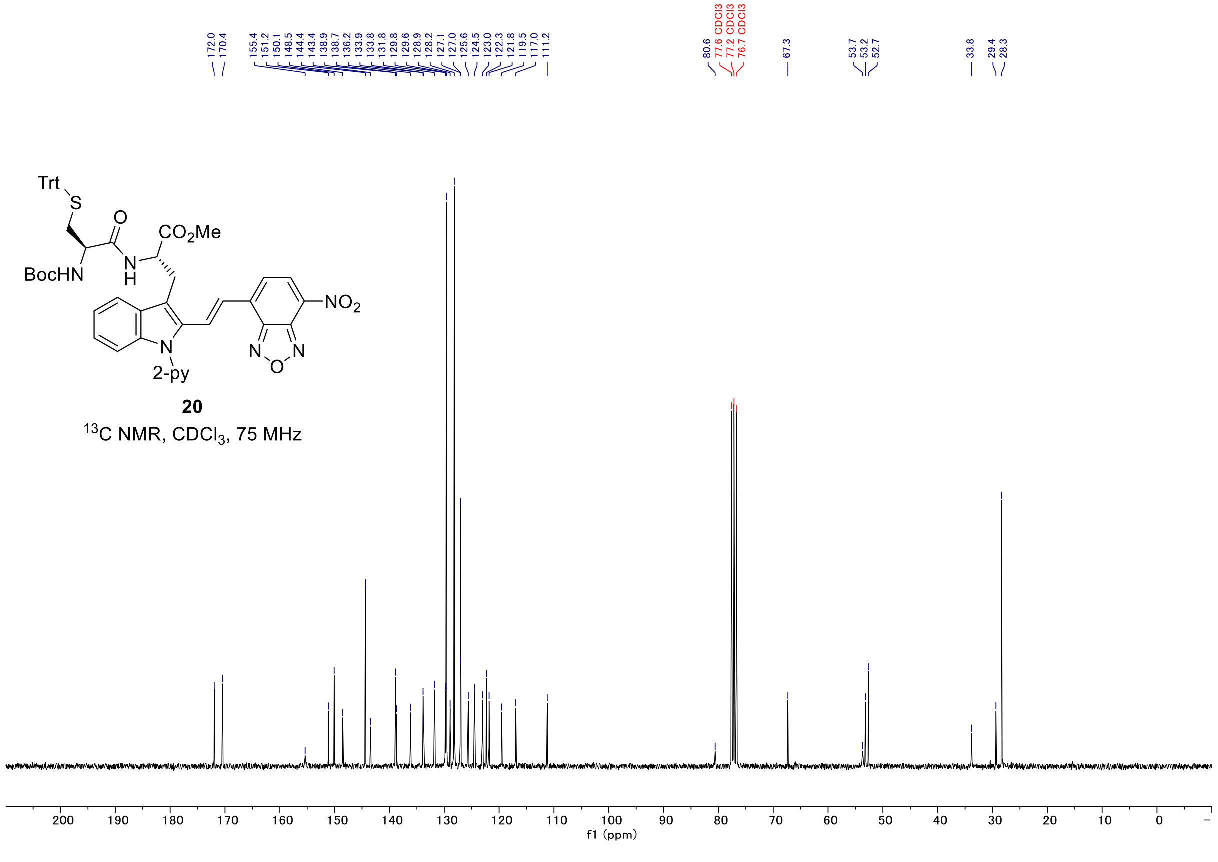

20

^{13}C NMR, $CDCl_3$, 75 MHz

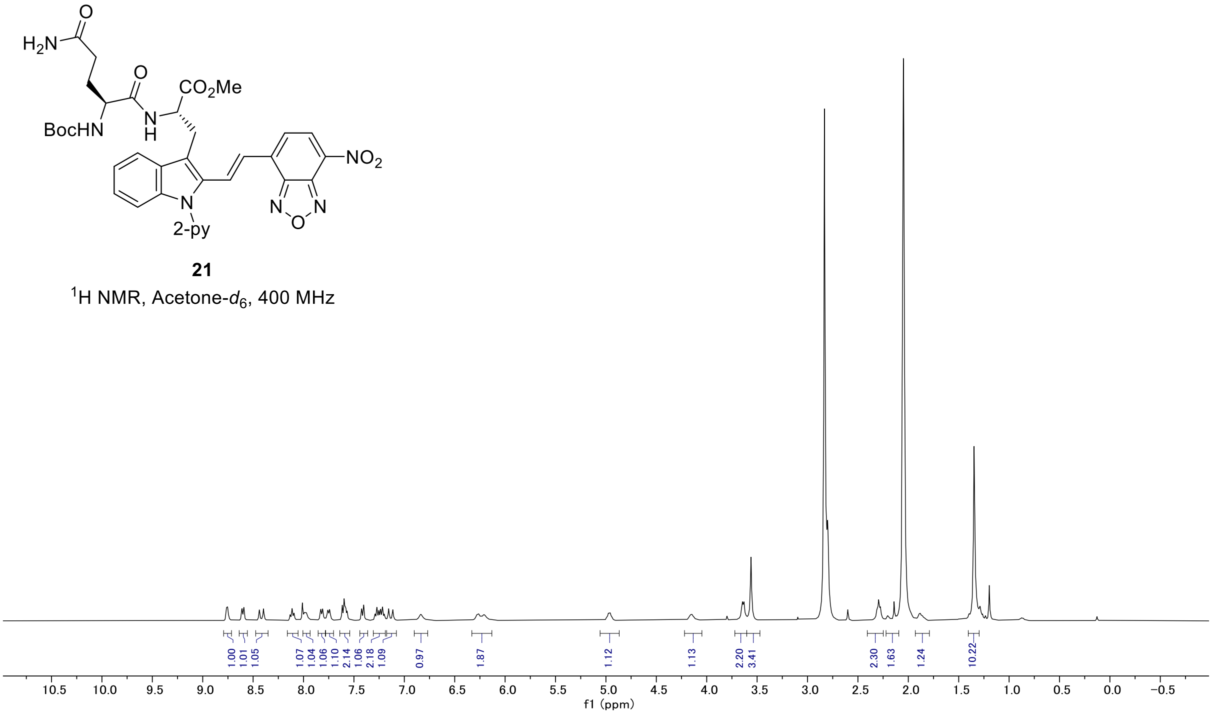

21

^{1}H NMR, Acetone-d_6, 400 MHz

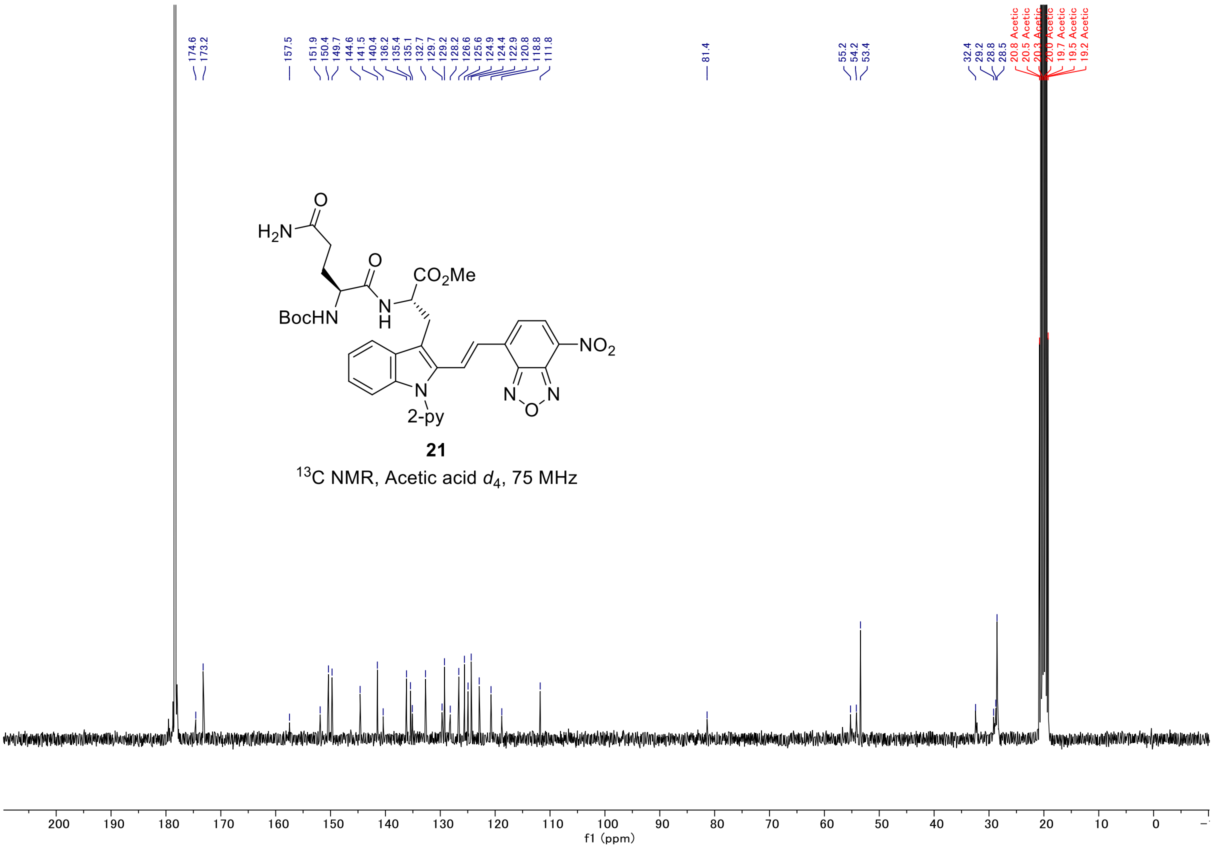

21

^{13}C NMR, Acetic acid d_4, 75 MHz

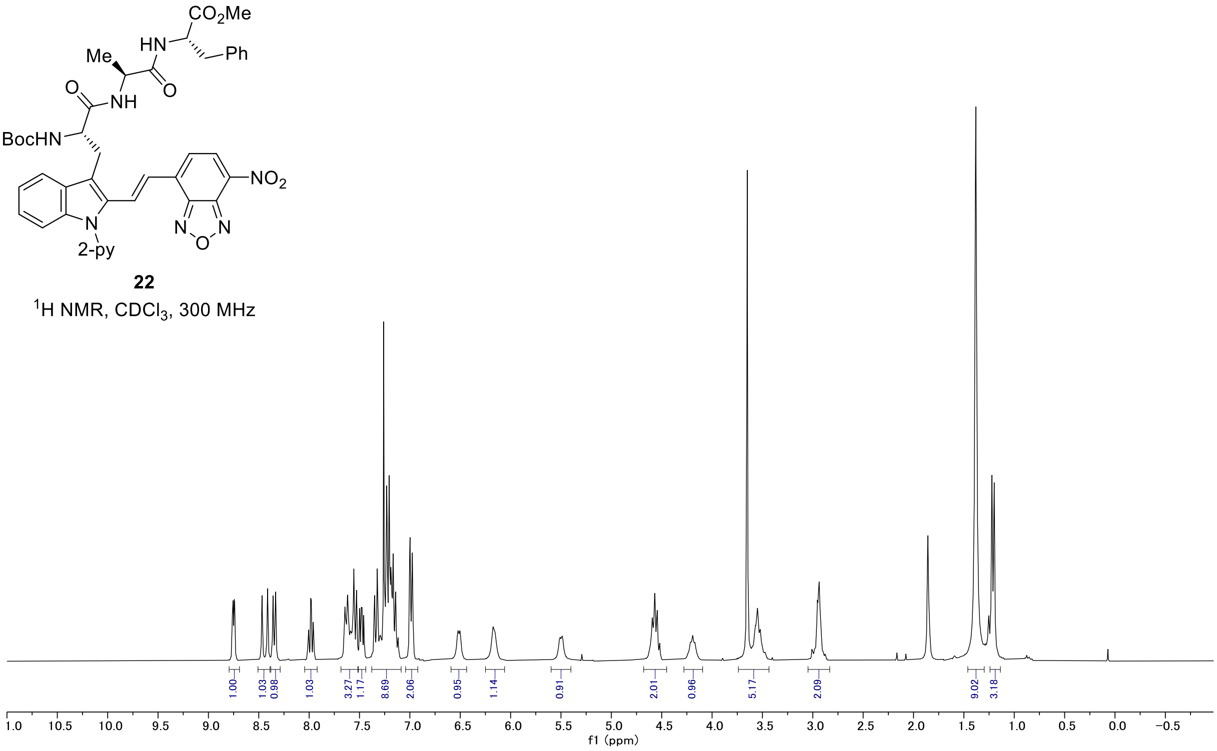

22

^{1}H NMR, $CDCl_3$, 300 MHz

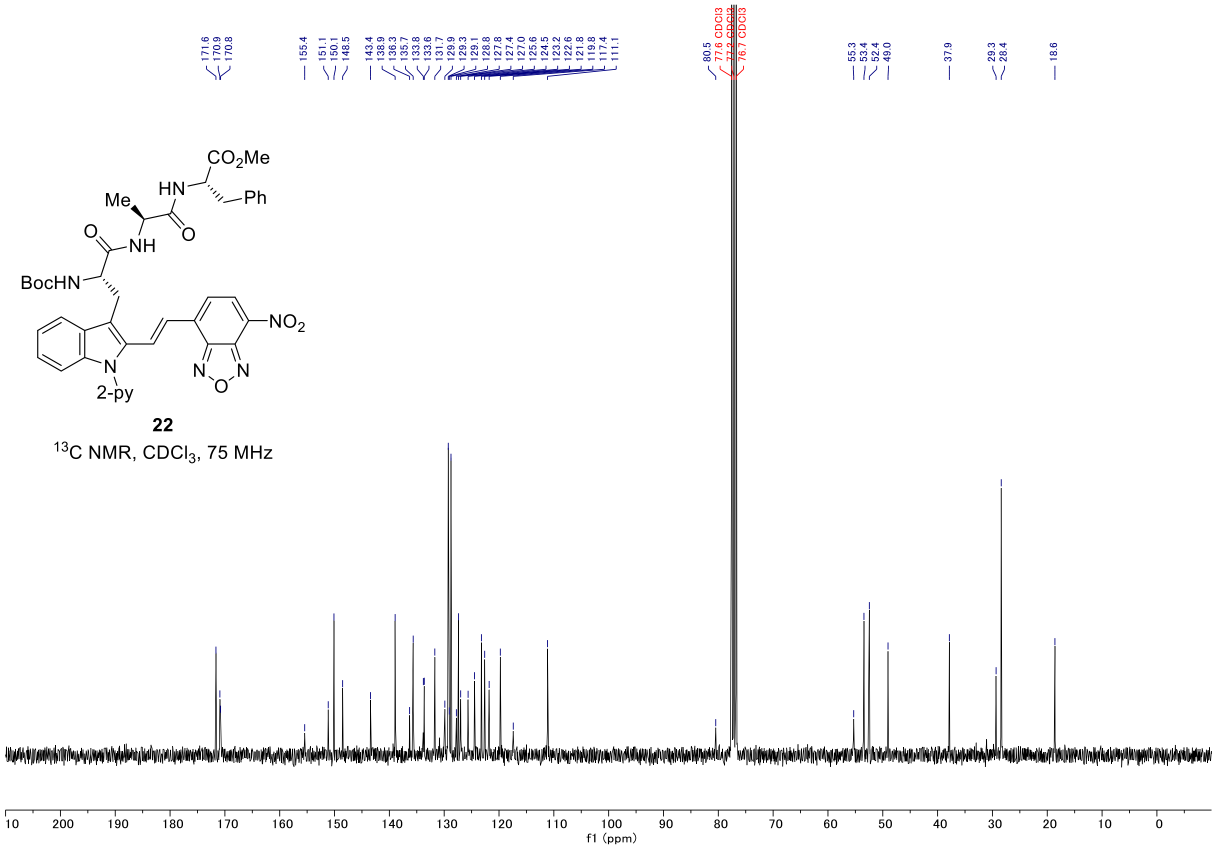

22

^{13}C NMR, $CDCl_3$, 75 MHz

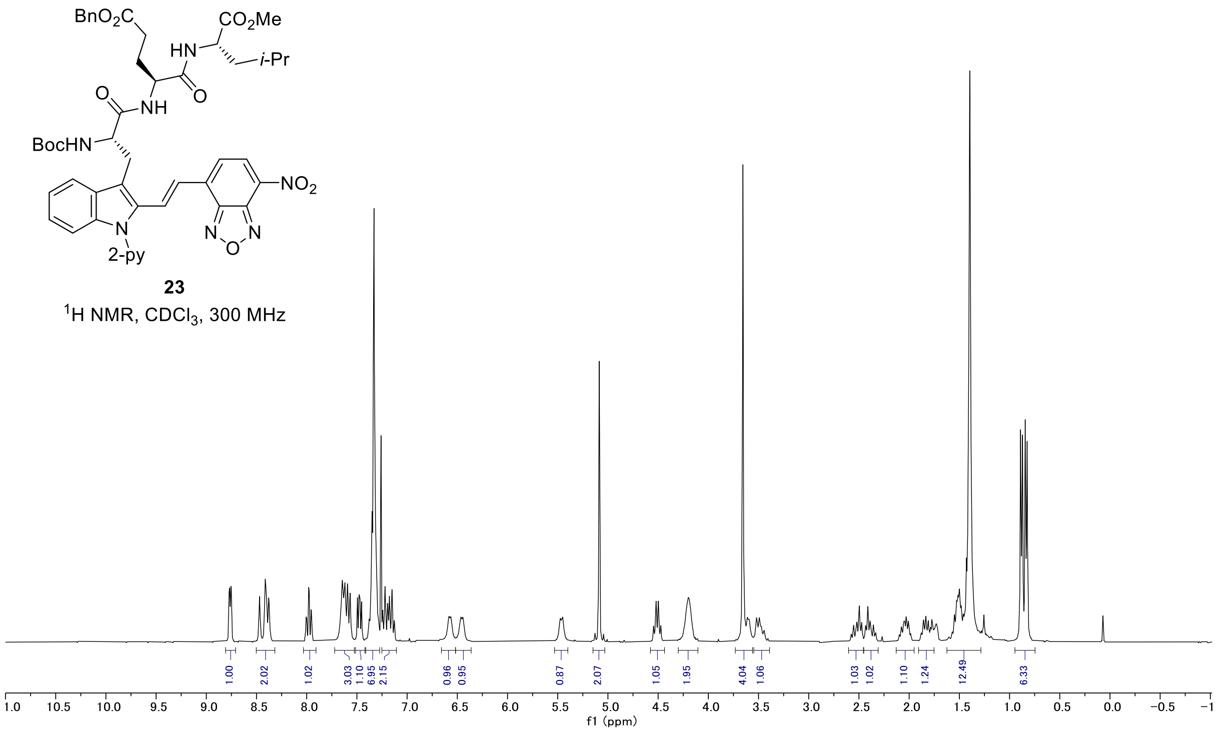
BnO2C
CO2Me
HN
i-Pr
O
NH
O
BocHN
NO2
N
N
O
N
2-py
23
1H NMR, CDCl3, 300 MHz
1.00
2.02
1.02
3.03
1.10
6.95
2.15
0.96
0.95
0.87
2.07
1.05
1.95
4.04
1.06
1.03
1.02
1.10
1.24
12.49
6.33
f1 (ppm)

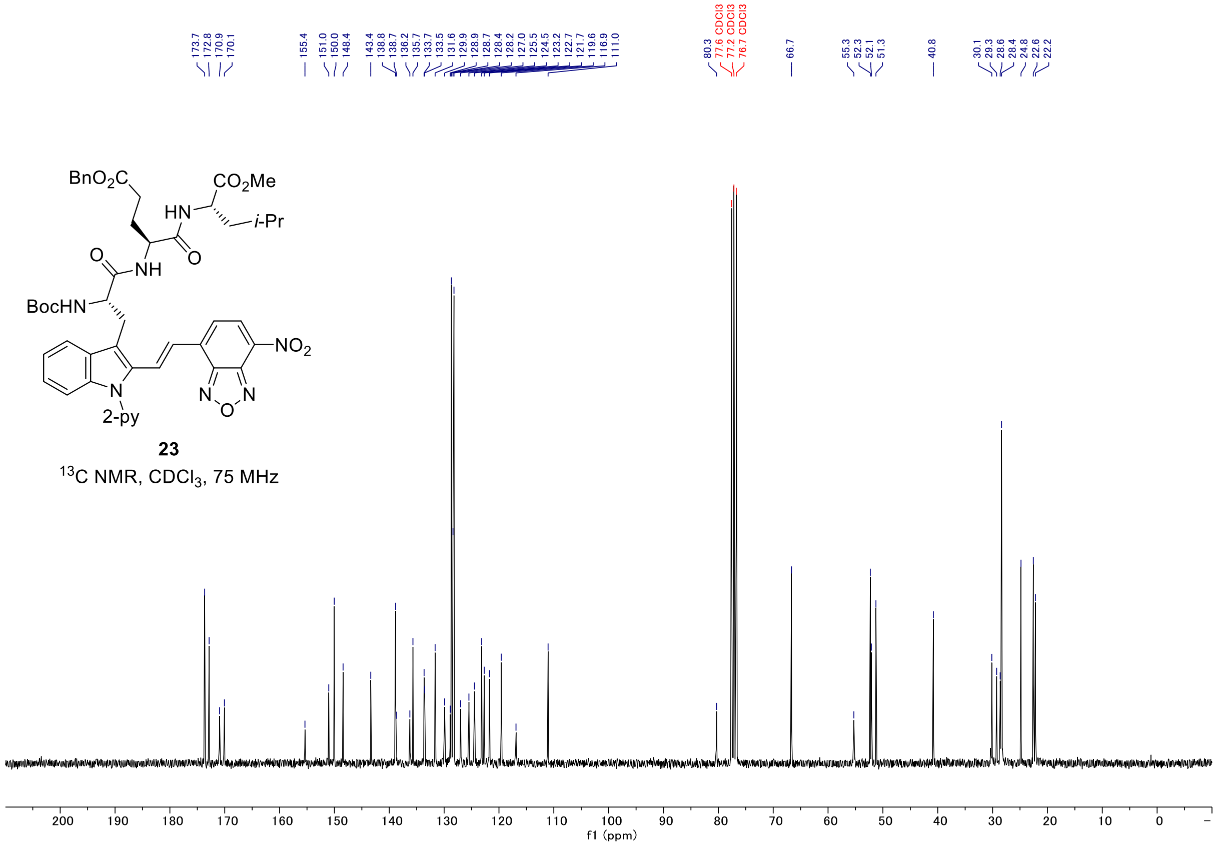
173.7
172.8
170.9
170.1
155.4
151.0
150.0
148.4
143.4
138.8
138.7
136.2
135.7
133.7
133.5
131.6
129.9
128.9
128.7
128.4
128.2
127.0
125.5
124.5
123.2
122.7
121.7
119.6
116.9
111.0
80.3
77.6 CDCl3
77.2 CDCl3
76.7 CDCl3
66.7
55.3
52.3
52.1
51.3
40.8
30.1
29.3
28.6
28.4
24.8
22.6
22.2
BnO2C
CO2Me
HN
i-Pr
O
NH
O
BocHN
NO2
N
N
O
N
2-py
23
13C NMR, CDCl3, 75 MHz
f1 (ppm)

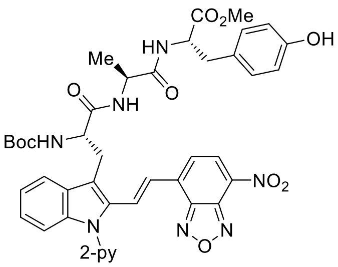

24

^{1}H NMR, $CDCl_3$, 400 MHz

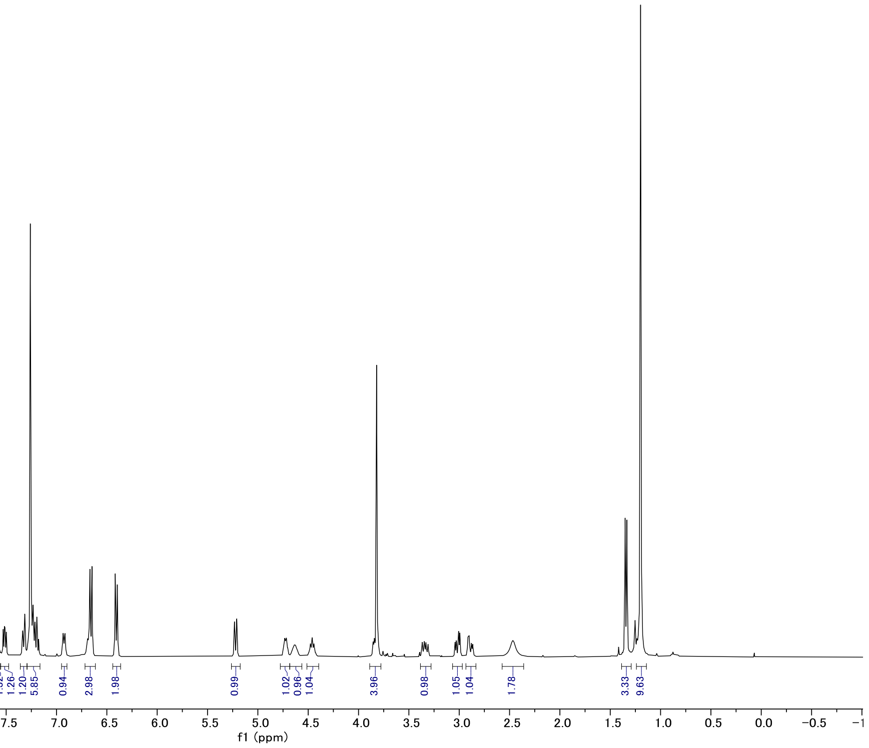

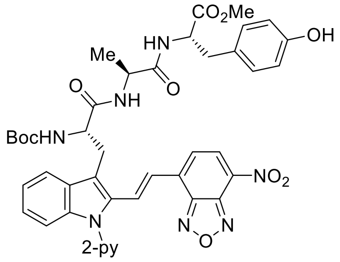

24

^{13}C NMR, $CDCl_3$, 101 MHz

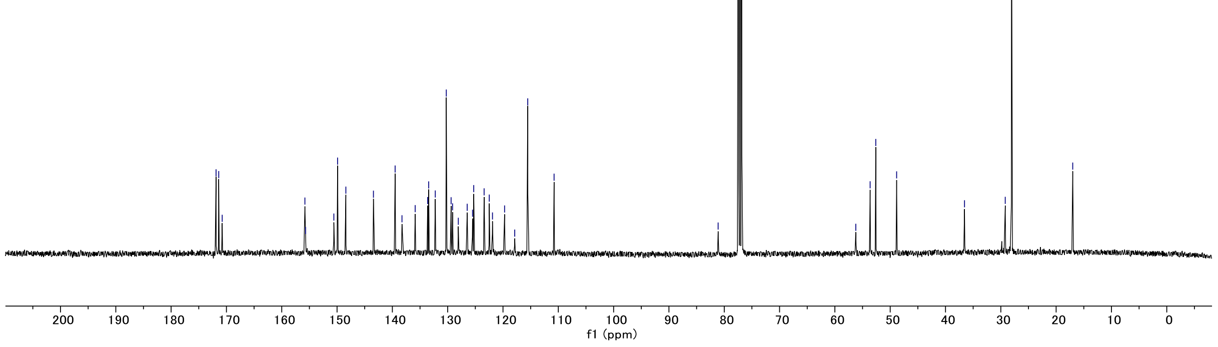

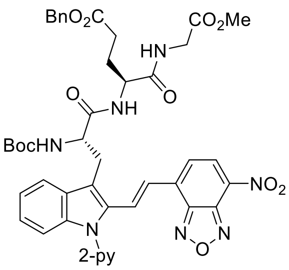

25

^{1}H NMR, $CDCl_3$, 300 MHz

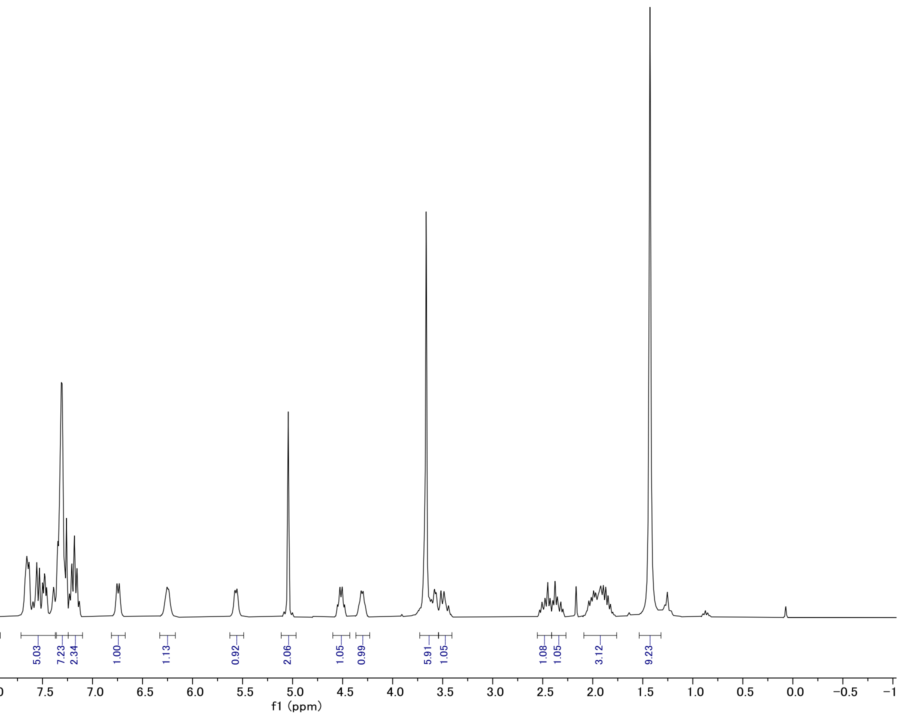

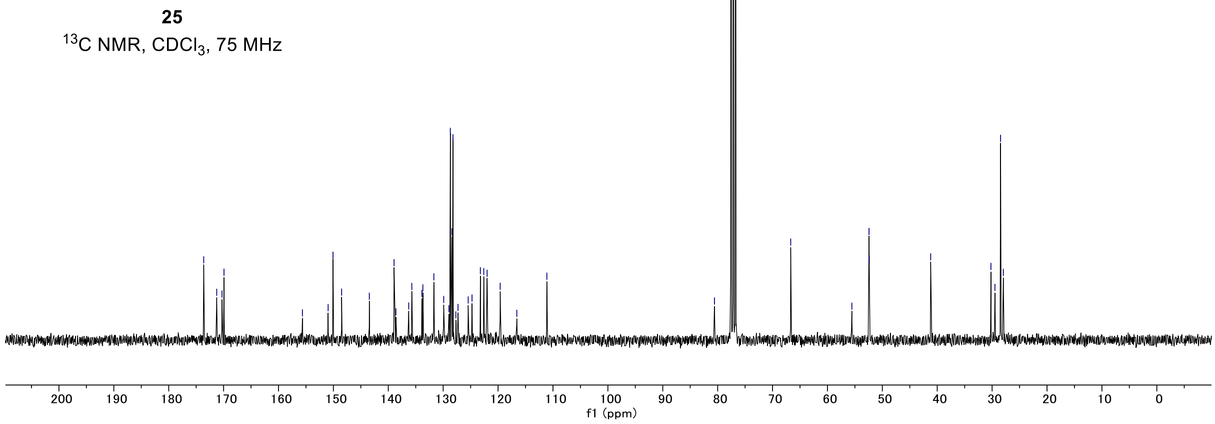

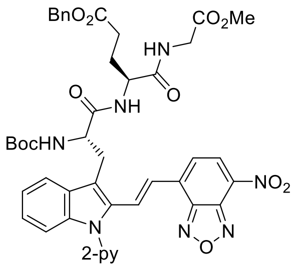

25

^{13}C NMR, $CDCl_3$, 75 MHz

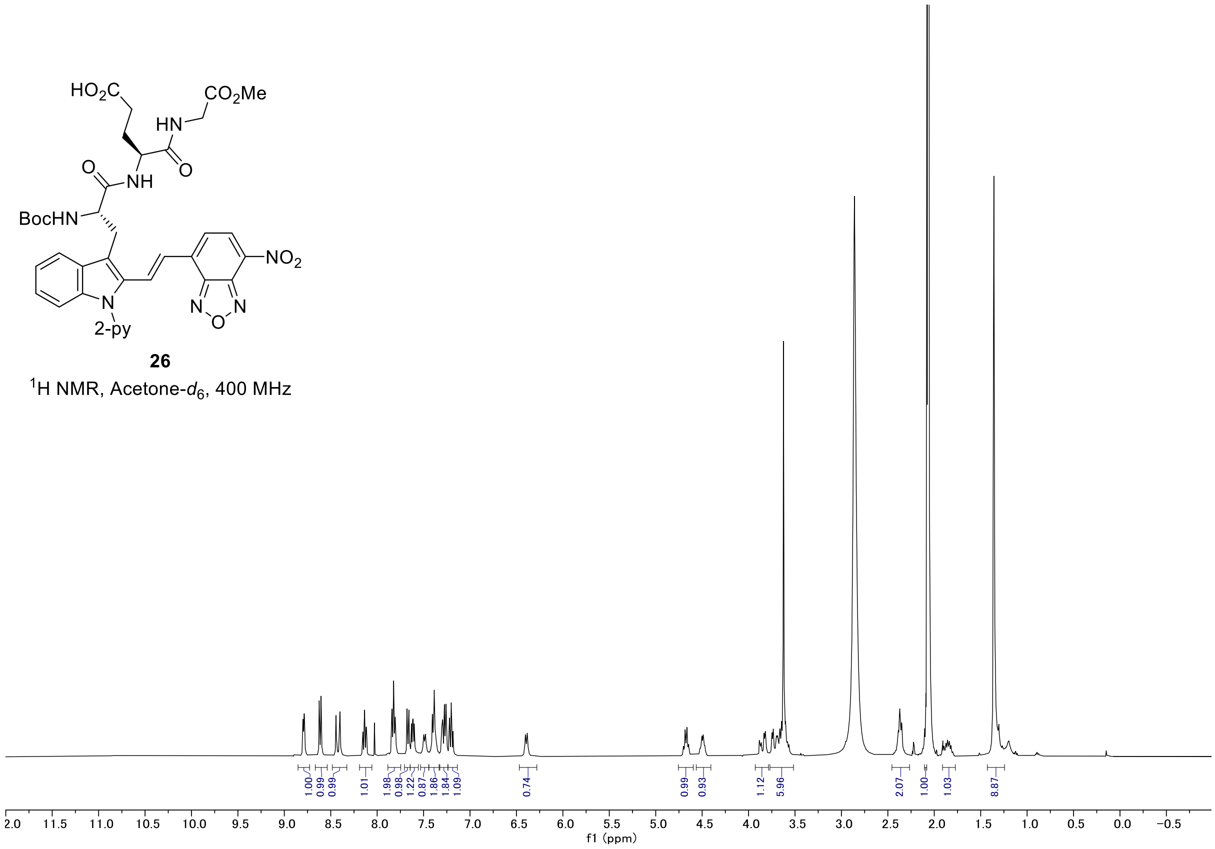
HO2C
CO2Me
HN
O
NH
O
BocHN
NO2
N
N
O
N
2-py
26
1H NMR, Acetone-d6, 400 MHz
1.00
0.99
0.99
1.01
1.98
0.98
1.22
0.87
1.86
1.84
1.09
0.74
0.99
0.93
1.12
5.96
2.07
1.00
1.03
8.87
f1 (ppm)

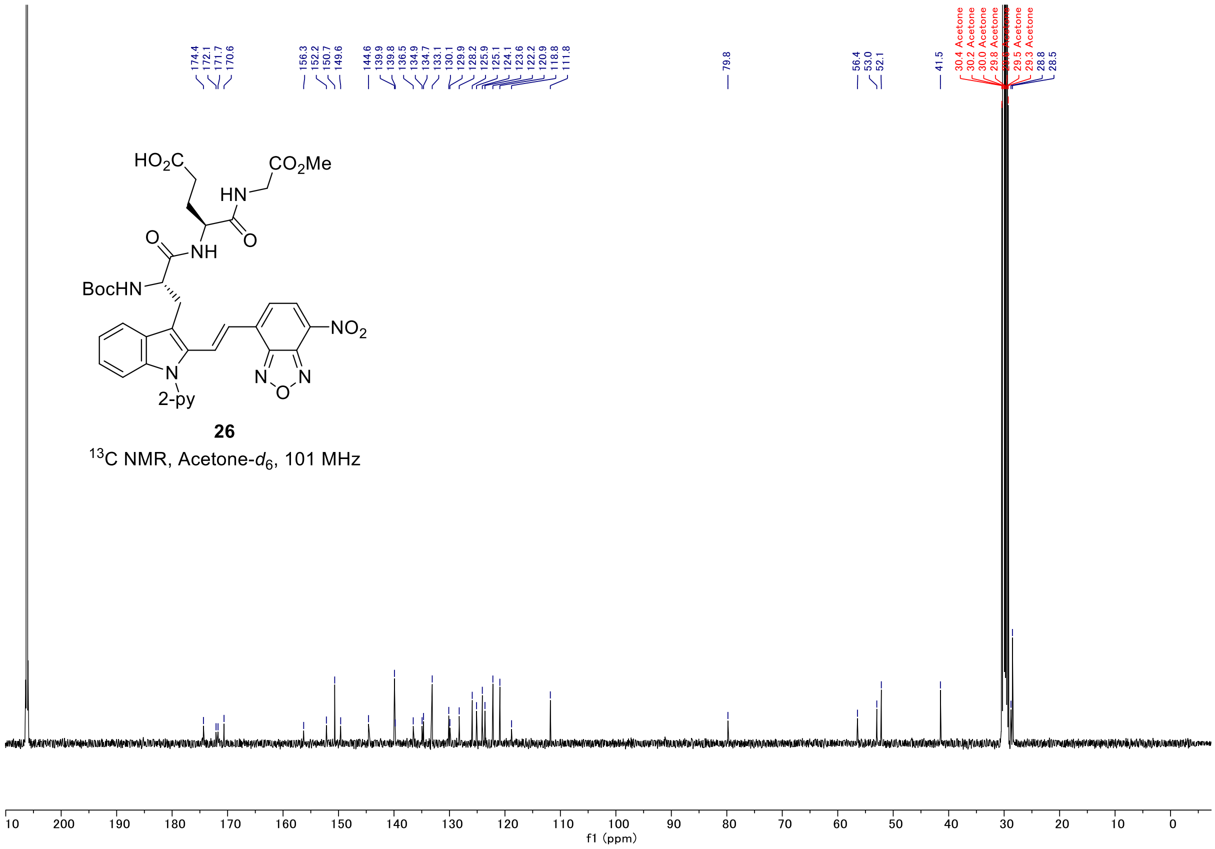
174.4
172.1
171.7
170.6
156.3
152.2
150.7
149.6
144.6
139.9
139.8
136.5
134.9
134.7
133.1
130.1
129.9
128.2
125.9
125.1
124.1
123.6
122.2
120.9
118.8
111.8
79.8
56.4
53.0
52.1
41.5
30.4 Acetone
30.2 Acetone
30.0 Acetone
29.8 Acetone
29.5 Acetone
29.3 Acetone
28.8
28.5
HO2C
CO2Me
HN
O
NH
O
BocHN
NO2
N
N
O
N
2-py
26
13C NMR, Acetone-d6, 101 MHz
f1 (ppm)

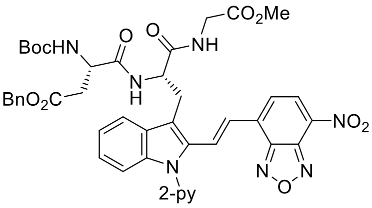

27

^{1}H NMR, $CDCl_3$, 400 MHz

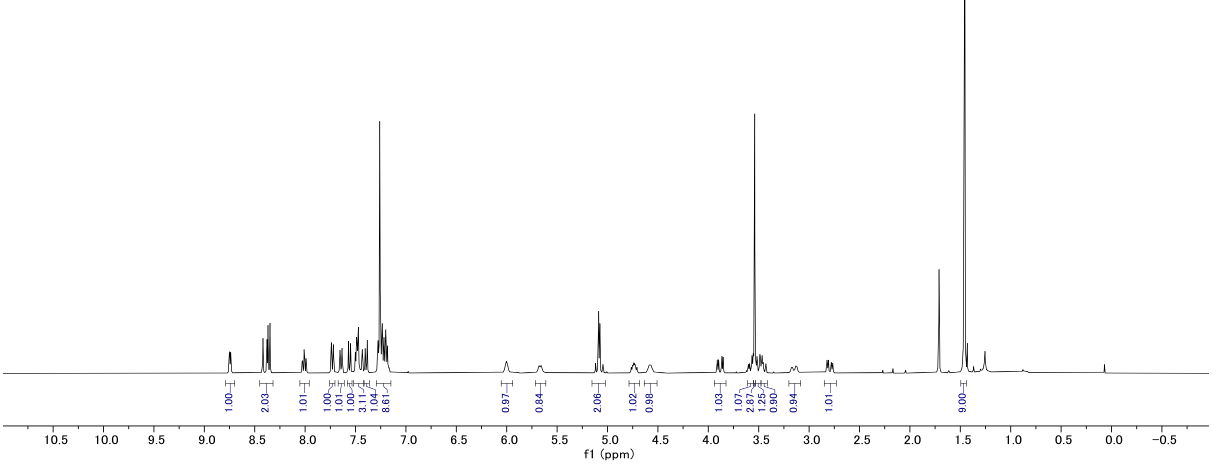

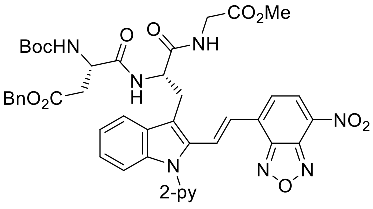

27

^{13}C NMR, $CDCl_3$, 101 MHz

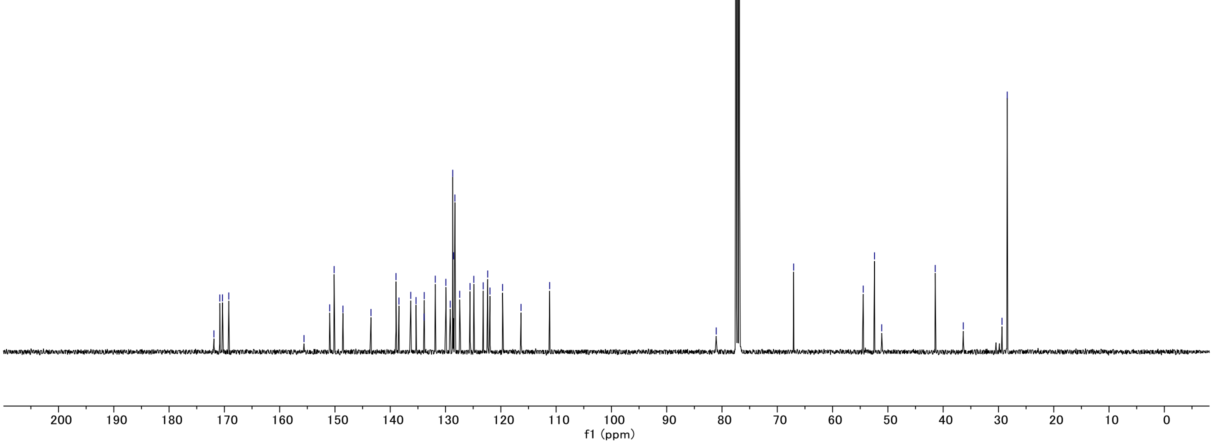

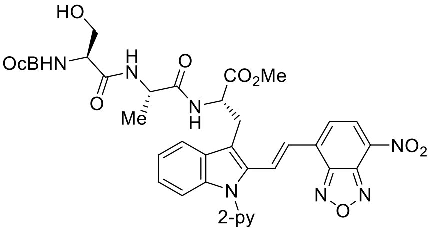

28

^{1}H NMR, $CDCl_3$, 400 MHz

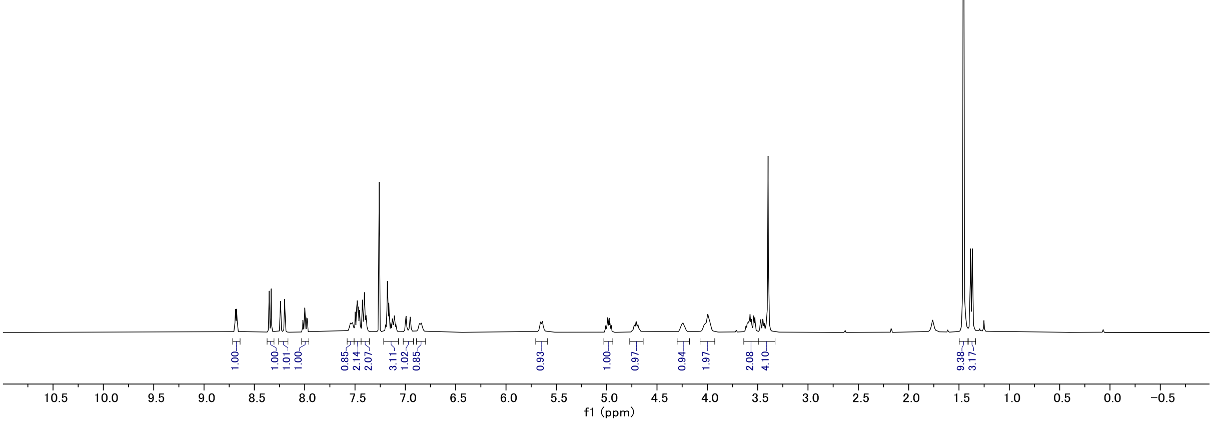

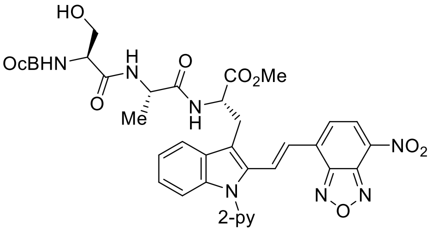

28

^{13}C NMR, $CDCl_3$, 101 MHz

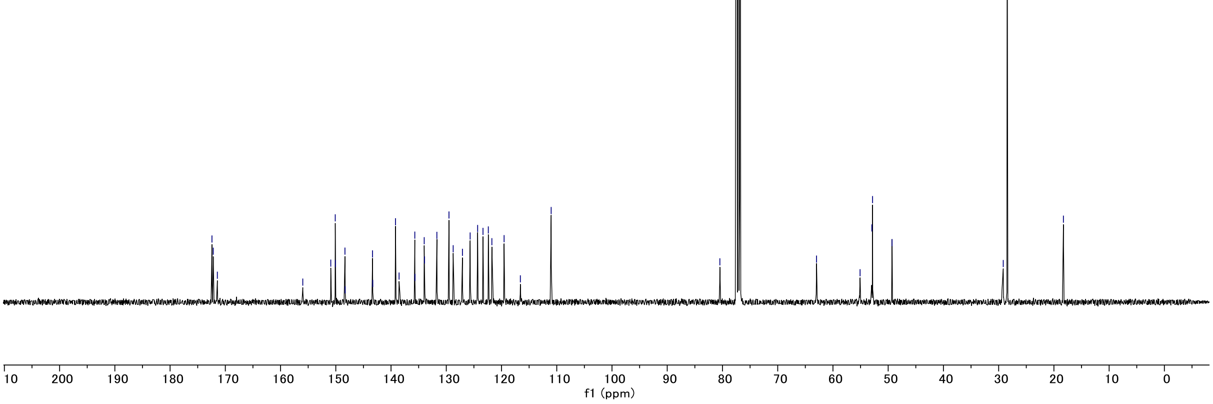

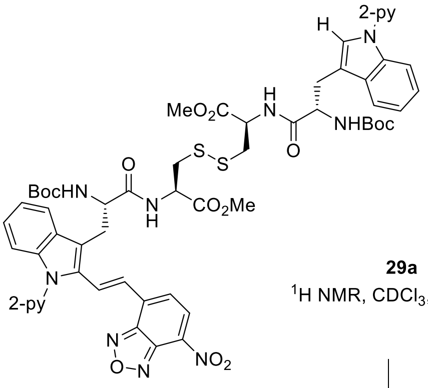

29a

^{1}H NMR, $CDCl_3$, 300 MHz

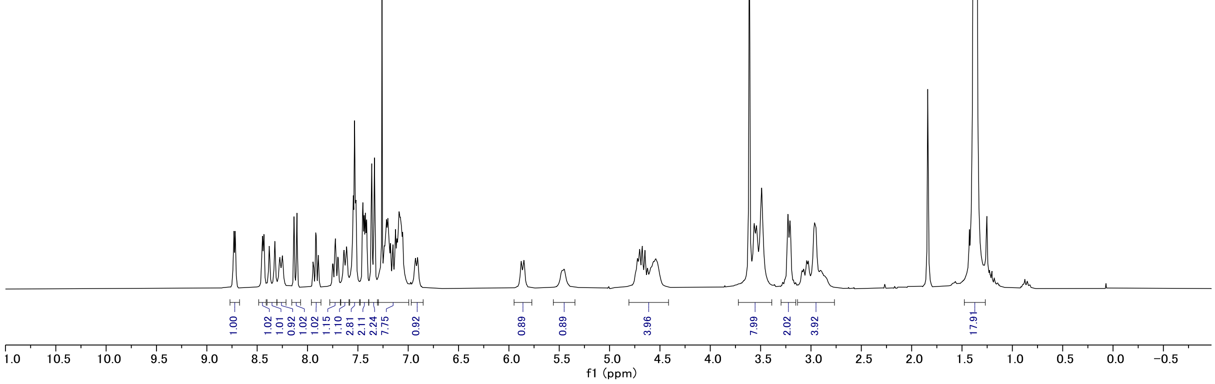

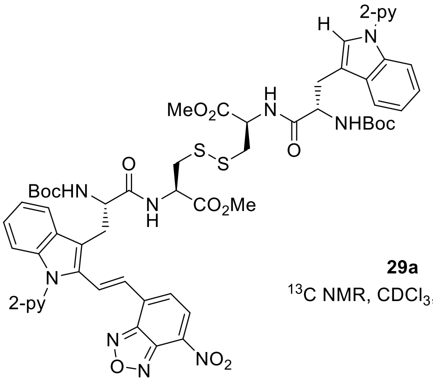

29a

^{13}C NMR, $CDCl_3$, 101 MHz

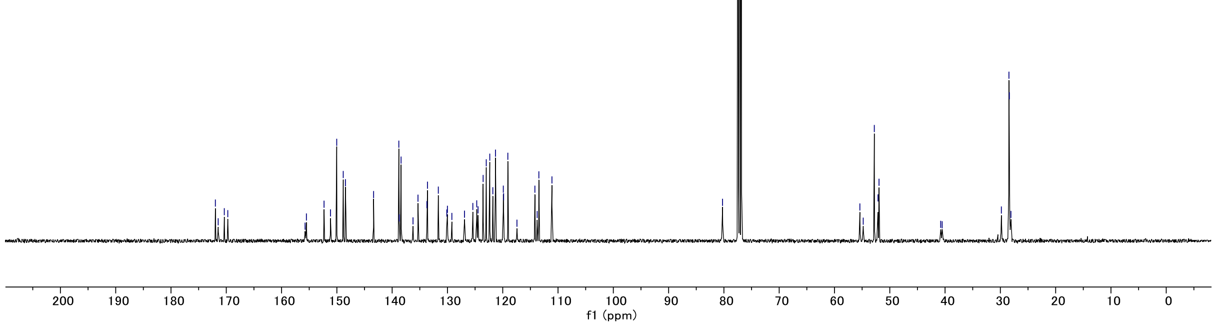

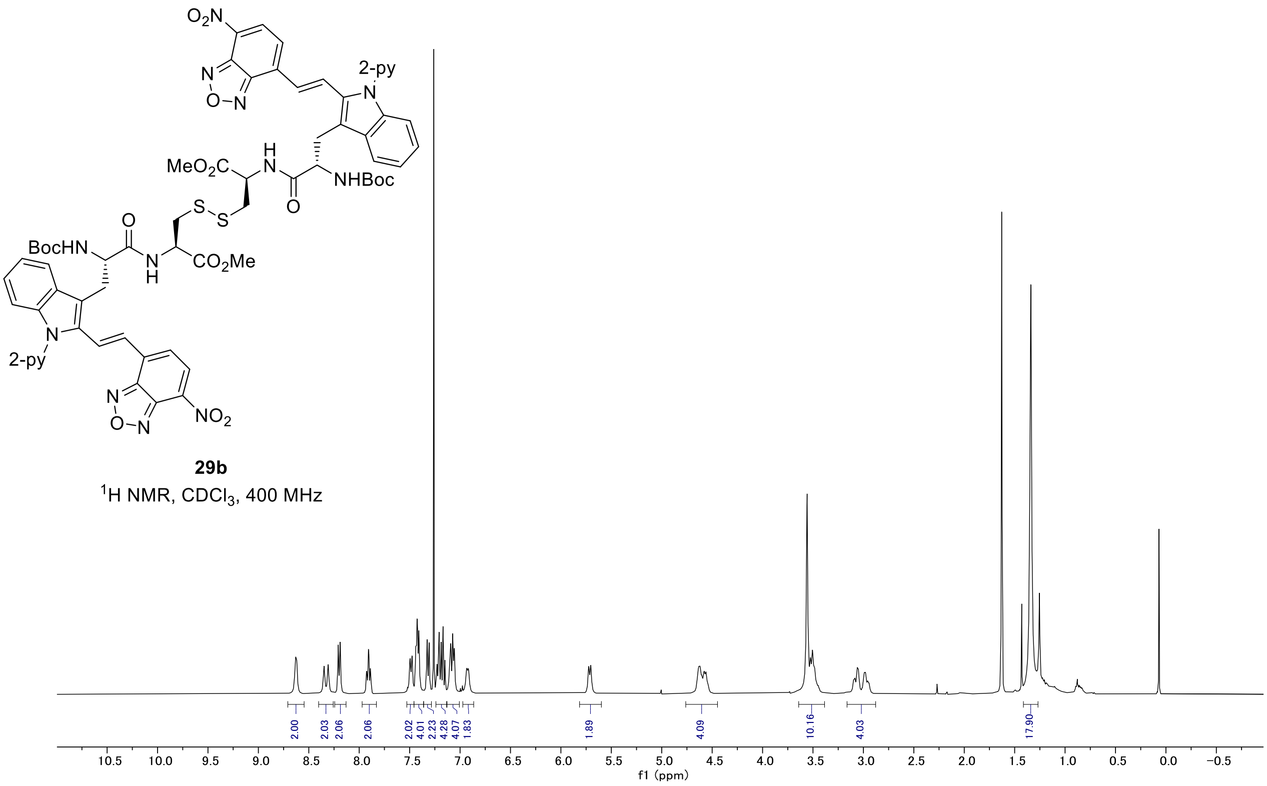

29b

^{1}H NMR, $CDCl_3$, 400 MHz

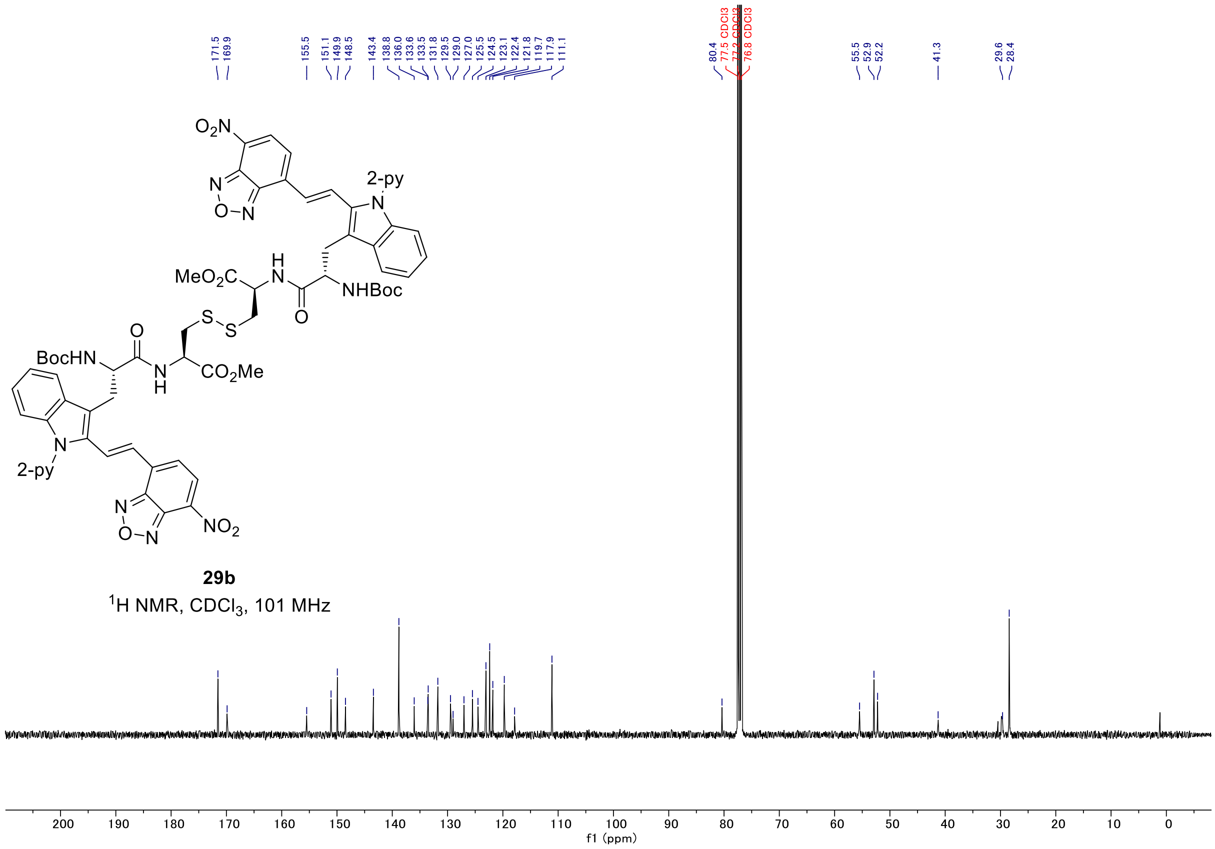

29b

^{1}H NMR, $CDCl_3$, 101 MHz

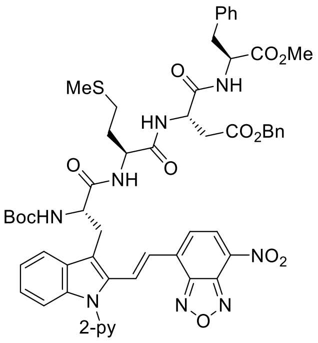

30

^{1}H NMR, $CDCl_3$, 400 MHz

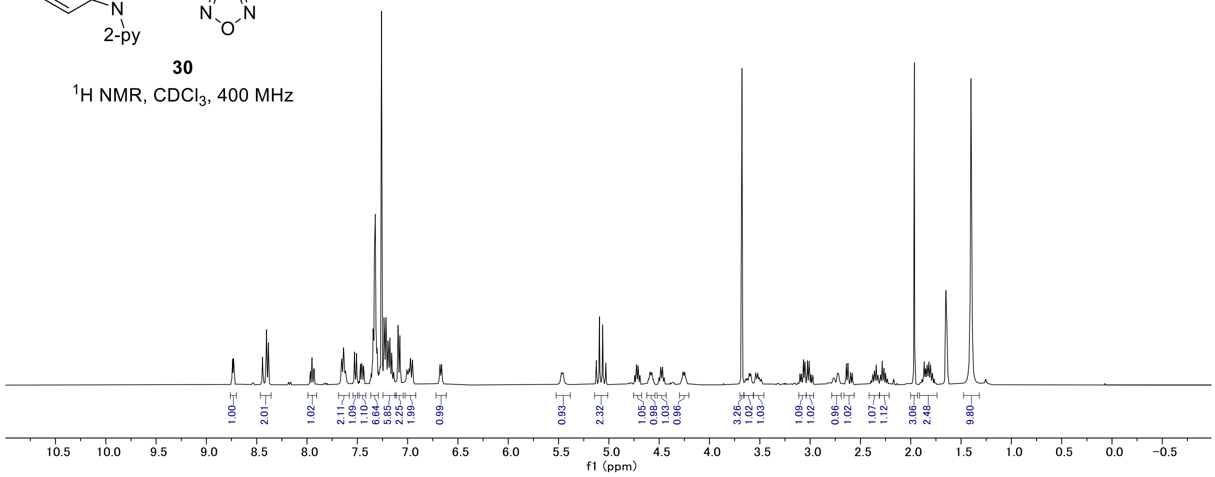

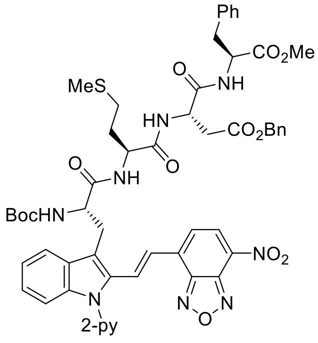

30

^{13}C NMR, $CDCl_3$, 101 MHz

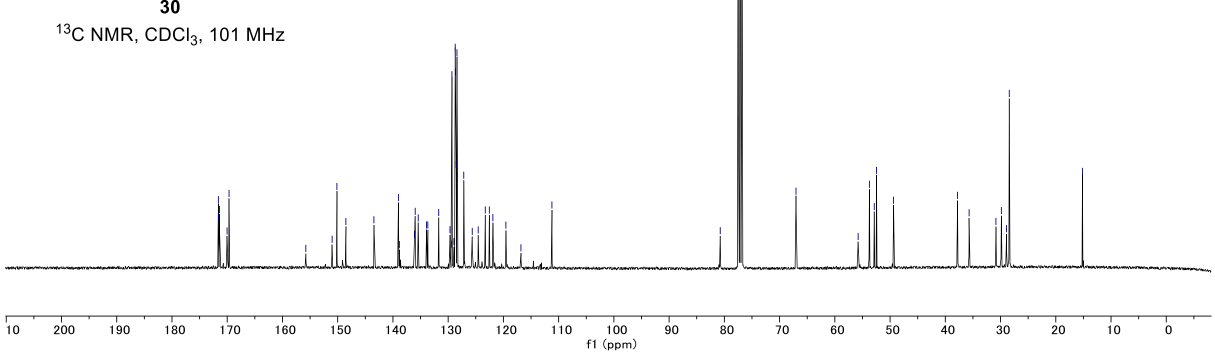

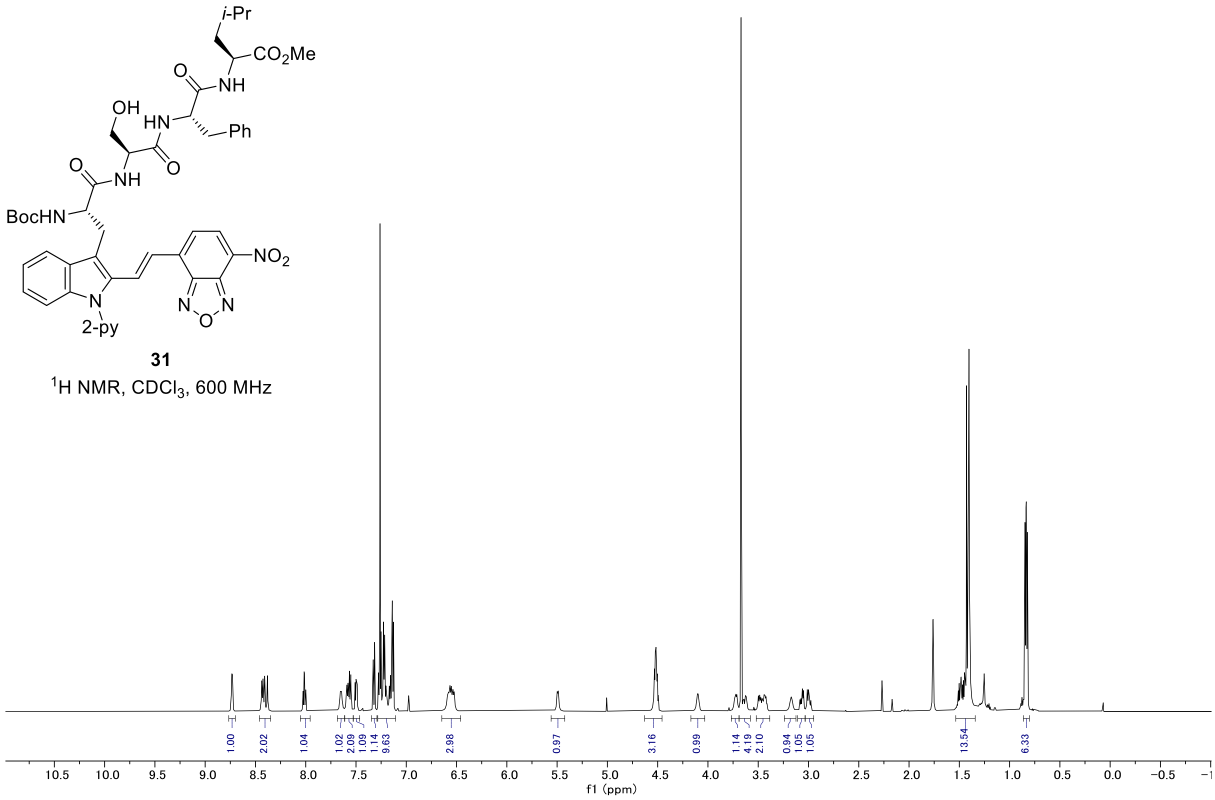
31
^{1}H NMR, $CDCl_3$, 600 MHz
f1 (ppm)

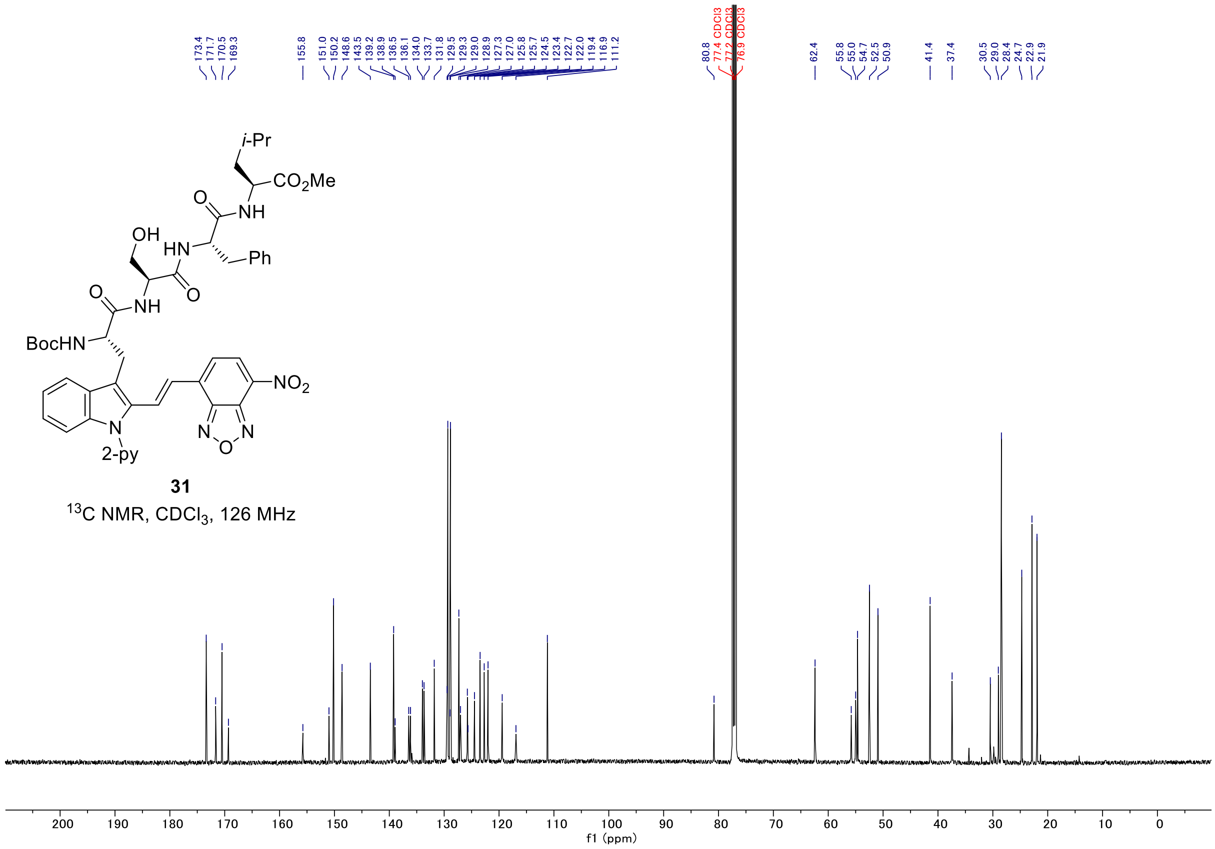
31
^{13}C NMR, $CDCl_3$, 126 MHz
f1 (ppm)
